# The role of early synthetic materials degradation in the downfall of the Ansaldo A.1, an Italian World War I biplane fighter

**DOI:** 10.1038/s41598-023-39164-9

**Published:** 2023-07-27

**Authors:** Jacopo La Nasa, Alessio Ceccarini, Riccardo Ducoli, Antonella Manariti, Jeanette J. Lucejko, Ilaria Degano, Neva Capra, Lucia Giovannini, Maria Luisa Tomasi, Francesca Modugno, Maria Perla Colombini, Ilaria Bonaduce

**Affiliations:** 1 Department of Chemistry and Industrial Chemistry, Via Giuseppe Moruzzi 13, 56124 Pisa, Italy; 2 CISUP Centre for Instrument Sharing, University of Pisa, Pisa, Italy; 3 Soprintendenza per i beni culturali della provincia autonoma di Trento, Via San Marco 27, 38122 Trento, Italy

**Keywords:** Analytical chemistry, History of chemistry

## Abstract

From the Pioneer Era of the aviation to World War I the evolution of aircraft technology and chemical synthesis enabled a unique coexistence of traditional craftsmanship, artistic decoration practices, and technological advancements. The study of the materials used in these early years of aviation is still an uncharted territory: a vast portion of remaining planes has been partially or completely repaired and restored, usually by total replacement of the fabric. The Italian biplane Ansaldo A.1 (1918) is a fighter aircraft and is one of the few planes in the world that still preserves its own original materials. In the last years, the fabric sections of the airplane have started to become brittle and loose cohesion, severely compromising the integrity of the aircraft, and resulting in a general alteration of the pictorial layers of the painted sections. A chemical investigation was undertaken to unveil the materials, and to elucidate the causes of the degradation. This study presents one of the first steps into the study of early historical aircrafts, defining the background for the conservation plans to preserve these objects for future generations.

## Introduction

An increasing number of museums has recently introduced historical airplanes in their collections and exhibitions, making them part of our Cultural Heritage. From the first Wright Brothers’ flights to the latest air- and space-crafts, a wide variety of new synthetic organic and inorganic materials has been introduced and tested to push forward the potential of aviation technology and to improve aeronautical performances. In the period from the Pioneer Era of the aviation until the end of World War I (1906–1918), advancements in chemical science^1–3^ and the fast evolution of aircraft technology led to the harmonious coexistence of traditional craftsmanship with scientific innovation. The latest technical and mechanical discoveries were treated as actual works of art, thus making the aesthetic component as important as the technological advancement. Aviation materials were chosen to be suitable for the planes to take flight, but also to act as artistic decorations. These materials were generally tested and developed to be suitable and stable for the limited lifetime of the aircraft, while their long-term durability as heritage materials to be preserved and displayed in museums for future generations is at present extremely difficult to assess. Nowadays, the limited knowledge of the composition and behavior of early aeronautical materials drastically challenges the conservation of historical aircrafts.

**Figure 1:**
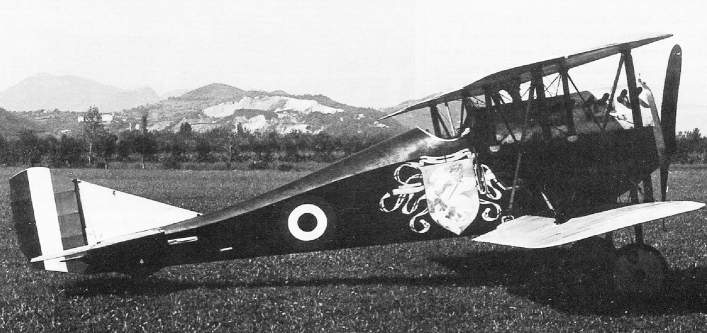
Early photography of the Ansaldo A.1 Balilla.

**Figure 2:**
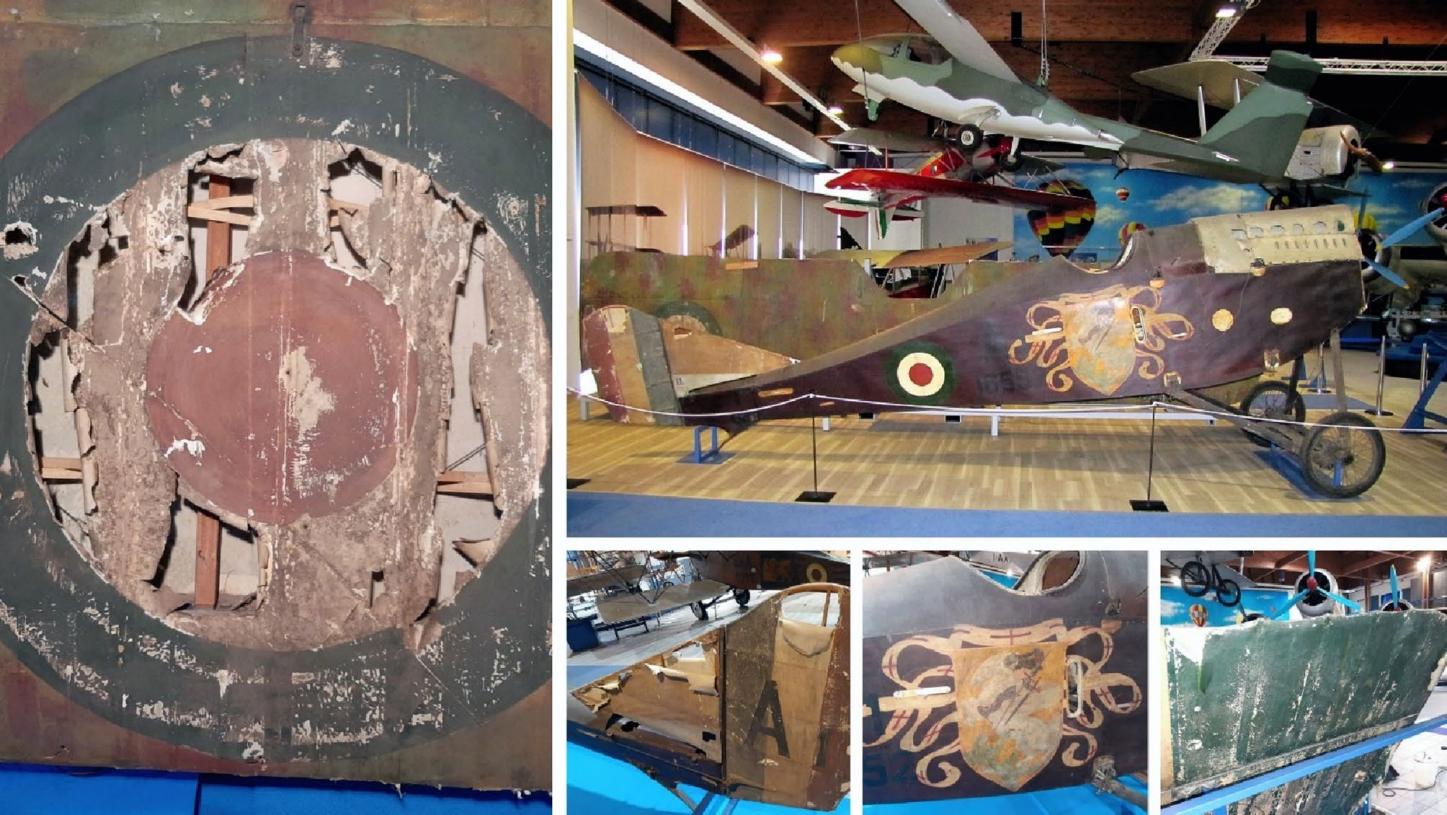
State of conservation of the Ansaldo A.1 Balilla in 2020 before the conservation campaign.

The degradation of the materials constituting the biplane Ansaldo A.1 (1918) is the result of critical concurrent chemical alteration processes, representing a “perfect storm” in this complex state of the art (Fig. 1). This model of biplane was produced during the last two years of World War I, and it is still constituted by its own original materials, making it a unique case study^4^. The unicity of this biplane also lies in the decoration of the fuselage, which makes the aircraft an actual flying painting. The plane consists of a wooden airframe covered with fabric. A lacquer (dope) was applied to tighten and stiffen the fabric stretched over the airframe. On the fuselage, “Saint George killing a dragon” was painted by the artist Amos Nattini (1892–1985). The fuselage and the upper wing of the airplane are currently on display at the Caproni Aviation Museum in Trento, Italy. In the last years, the fabric sections of the airplane have started to become brittle and loose cohesion, severely compromising the integrity of the aircraft and resulting in a general alteration of the pictorial layers (Fig. 2).

This study describes the chemical investigation undertaken to elucidate the causes of degradation and to inform the conservation of the aircraft. Optical and scanning electron microscopy, infrared spectroscopy, liquid chromatography, mass spectrometry, and analytical pyrolysis coupled with gas chromatography and mass spectrometry were used to characterize both the organic and inorganic materials. In addition to the study of a set of micro-samples from different sections of the plane, the scientific campaign entailed also the characterization of the volatile organic compounds (VOCs) in the museum environment, linked to the emissions produced by the plane.

## Results

The cross-sections of the fabric samples showed under the optical microscope only two layers besides the textile support: a dope and a paint layer (Supporting Information –SI–S2).

Analytical pyrolysis coupled with gas chromatography and mass spectrometry (Py-GC–MS) was used to characterize both the origin of the fabric and the dope (SI–S3). Pyrolysis markers characteristic of silk were detected, with diketopiperazines and phenol derivatives as the most abundant species^5^. Cotton was the material used in one patch of the fabric covering, based on the detection of anhydro sugars, with 1,4:3,6-dianhydro-α-D-glucopyranose and levoglucosan as characteristic markers in the pyrogram^5^. Although the use of silk is consistent with the technology of the period^6^, the choice of this material is remarkable, since it was more expensive and valuable than cotton or linen, which were commonly used to cover the body of aircrafts.

The lacquer used as dope for the fabric was cellulose acetate: the material is identified by the detection, among the pyrolysis products, of acetic acid, acetoxymethyl furfural and several acetylated products deriving from the thermal degradation of carbohydrates^7–9^. The presence of cellulose acetate was further confirmed by infrared spectroscopy analysis (SI–S4). In the dope, relatively high amounts of camphor were also detected, one of the first known and common plasticizer of cellulose acetate^10,11^. The first aircraft models were generally produced using dopes based on cellulose nitrate, characterized by a higher inflammability and toxicity with respect to cellulose acetate^12,13^. The latter was more expensive at the time, but it was known to have better mechanical properties compared to cellulose nitrate^14,15^, and thus preferred by the Italian Air Force during WWI^16^.

The analyses performed by Py-GC–MS on the painted portion of fabric highlighted the presence of the pyrolytic molecular profile of a drying oil: short chain saturated and unsaturated aliphatic acids, fatty acids (palmitic and stearic acids) and dicarboxylic acids (sebacic, suberic and azelaic acids)^17^. Retene, dehydroabietic acid and other diterpene derivatives were also detected, which are characteristic of the pyrolysis of pine pitch^18^. The identification of only one paint layer under the optical microscope (SI–S2) suggested that drying oil and pine pitch were mixed prior to the application, probably to improve the resistance of the plane to atmospheric events. Liquid chromatography coupled with high resolution mass spectrometry was applied to further characterize the nature of the drying oil: a series of specific unoxidized and oxidized acylglycerols deriving from the curing of triacylglycerols containing linoleic and linolenic acids as acyls substituents (SI–S5) were identified, which are consistent with the use of linseed oil as paint binder^19^.

The resin-embedded cross-sections of the samples were analyzed using a field emission scanning electron microscope (FEG-SEM) to characterize the inorganic materials by X-ray microanalysis (SI—S9). The elemental analysis allowed us to hypothesize the presence of barium sulfate used as a filler in the cellulose acetate layer. This white pigment is well known to have excellent reflective properties and was thus used in the dope layer to improve its resistance to degradation induced by sunlight^20,21^.

The elemental distribution pointed at the presence of chromium oxide and lead carbonate as pigments in the camouflage pattern on the aircraft body, and Sienna and lead white as pigments in the red and white sections of the Italian flags.

**Figure 3:**
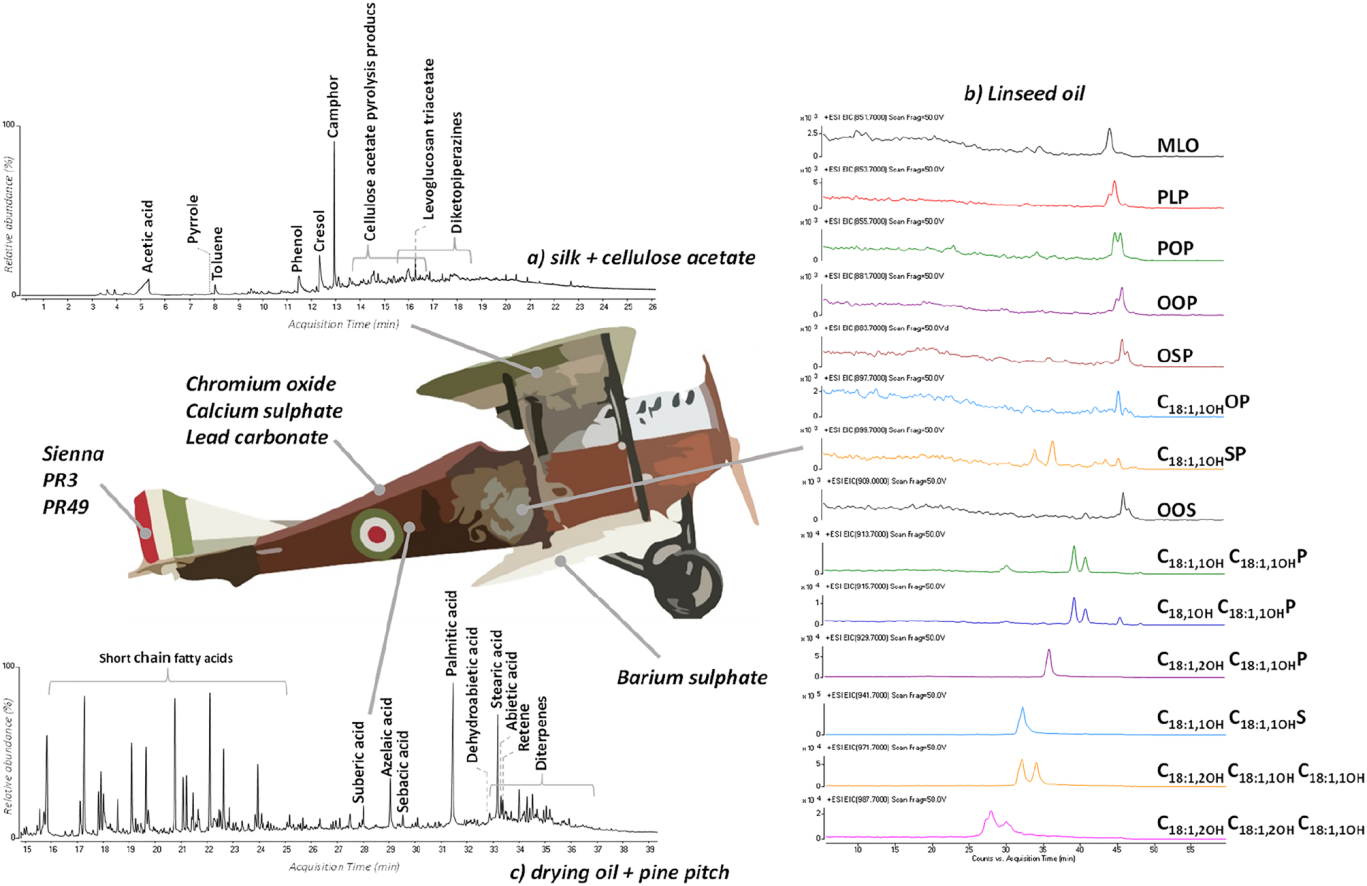
Materials identified in the different sections of the Ansaldo A.1 Balilla; (**a**): Py-GC–MS chromatogram obtained for a sample containing both silk and the dope layer; (**b**) Py-GC–MS chromatogram obtained for a paint sample with hexamethyldisilazane as derivatizing agent; (**c**) Extracted ion chromatograms obtained for the dichloromethane extract of a paint sample from the “Saint George and the dragon” painting; the triacylglycerols were detected as sodium adducts ([M + Na]^+^); Acyl substituents abbreviation list: M: myristyl (C_14_); P: palmityl (C_16_); L: linoleyl (C_18:2_); O: oleyl (C_18:1_); S: stearyl (C_18_); For the oxidized acyl substituents: C_x:y,zOH_ with x: n° of carbon atoms, y n° of unsaturation, and z n° of hydroxy groups.

The samples containing red and green pigments were also subjected to solvent extraction and analysis by liquid chromatography and mass spectrometry (SI–S6) to detect the possible occurrence of dyes or organic pigments. In the red samples, PR3 and PR49 were detected, which are synthetic organic pigments introduced in 1904 and 1899, respectively^22^. The analysis on the green samples were inconclusive. The absence of specific elements in the black sections of the plane except for carbon suggested the use of a carbon-based black pigment. The materials detected in all the analyzed samples are detailed in Table 1 and a summary is depicted in Fig. 3.

**Table 1:** Sample position, description (in bold), and summary of the materials detected in each of the samples.

Sample code	Description	Background	Paint layer
1	Upper Wing (upper surface), left Italian cockade flag, **red paint sample**	–	Linseed oil, PR49
2	Upper Wing (upper surface), left Italian cockade flag, **green sample with textile**	Silk, cellulose acetate, barium sulphate	Linseed oil, pine pitch, lead carbonate, chromium oxide, sodium carbonate
3	Upper Wing (upper surface), left Italian cockade flag, **white paint sample with textile**	Silk, cellulose acetate, barium sulphate	Linseed oil
6	**White pant sample** from the horse of the “Saint George killing a dragon” painting on the fuselage	Barium sulphate	Linseed oil, pine pitch, lead carbonate
7	Tail horizontal stabilizer, **red paint sample with textile**	Silk, cellulose acetate, barium sulphate	Linseed oil, pine pitch, Sienna
8	Tail horizontal stabilizer, **black paint sample with textile**	–	Linseed oil, pine pitch
9	Tail horizontal stabilizer, **green paint sample with textile**	Silk, cellulose acetate, barium sulphate	Linseed oil, pine pitch, chromium oxide, sodium carbonate
10	Upper wing (lower surface), Italian red flag area, **red paint sample**	–	Linseed oil, pine pitch, Sienna, PR3, PR49
11	Upper wing (lower surface), Italian green flag area, **green paint sample with textile**	Silk, cellulose acetate, barium sulphate	Linseed oil, pine pitch chromium oxide, calcium sulphate, lead carbonate
12	Upper wing (lower surface), Italian white flag area, **yellow sample with textile**	Silk, cellulose acetate	–
13	Lower wing (lower surface), **green sample with textile**	Silk, cellulose acetate, barium Sulphate	Linseed oil, pine pitch, lead carbonate, chromium oxide, silica
14	Lower wing (lower surface), **green paint sample**	Barium Sulphate	Linseed oil, pine pitch, pine resin, calcium carbonate, chromium oxide
15	Lower wing (lower surface), **yellow sample** (dope sample)	Cellulose acetate	–
16	Lower wing, **cotton patch**	Cotton, paraffine	–

## Discussion

What is happening to the Ansaldo A.1 today? The chemical characterization of the materials showed that the biplane was built using a combination of a heterogeneous variety of natural and early synthetic materials. Among these, cellulose acetate is the first suspected cause of the dramatic deterioration of the conditions of the aircraft observed in the latest years.

It is well known that high levels of humidity can trigger the hydrolysis of acetic acid moieties producing acetic acid, with consequent auto-catalytic hydrolysis of cellulose acetate. The phenomenon is called *vinegar syndrome*, a degradation issue that is well documented for cellulose triacetate and has been encountered in several case studies related to the conservation of cinematographic films^23–28^.

Consequently, the volatile organic compounds emitted by the aircraft in the museum environment (SI–S7) were analyzed, showing mainly the presence of aldehydes, hydrocarbons, and acetic acid. Moreover, the VOCs profiles were also all characterized by the presence of camphor, indicating the coexistence of two different degradation phenomena, namely the hydrolysis of cellulose acetate and the loss of plasticizers. These resulted in the embrittlement and shrinkage of the dope layer, compromising its structural integrity and mechanical properties^29^.

pH was measured on a series of reference silk samples—which did not contain the dope layer—showing an average value of 6.1. This value is in agreement with those reported in the literature^30^. The analyses performed on the samples from the aircraft containing the dope showed an average pH of 4.5. Silk is a natural protein fiber, and the formation of acetic acid from the dope, with the consequent decrease in the pH value, had important consequences on silk structure. Exposure to an acidic environment has indeed been proven to be an important factor that could affect the ageing of any organic material^31^. In particular, it may cause the hydrolysis of the proteins in silk, compromising the mechanical properties of the fibers^32^.

The chemical changes undergone by the silk substrate and the dope, with consequent loss of structural integrity, must be considered responsible for the compromised cohesion of the paint layers, leading to paint losses in several areas. Fortunately, the degradation did not extend to the wood used for the frame: the Py-GC–MS analyses performed on two samples from the wooden structure highlighted holocellulose/lignin ratios consistent with well-preserved wood^33^ (SI–S8).

Unfortunately, the destiny of the Ansaldo A.1 was doomed since the day of its fabrication, due to the combined use of cellulose acetate and silk. The *vinegar syndrome* occurs naturally upon ageing, and it is an autocatalytic process, which can only be slowed down, but neither interrupted nor reversed^23–28^. And then COVID-19 happened. The lockdown enacted by the Italian government during the first stages of the pandemic meant that the Caproni Aviation Museum was closed for several months. In that period, the air conditioning and recycling system of the museum were turned off, resulting in an increase of the relative humidity of the museum environment, and in the stagnation of acetic acid emitted by the hydrolyzing cellulose acetate. Humidity and the increased acetic acid content in the vapor phase are the two most important factors that could have triggered and catalyzed the *vinegar syndrome*. In these conditions the degradation accelerated and the plane started to show structural and visual damages. As a consequence, a diagnostic and conservation campaign has been undertaken to consolidate the structure of Ansaldo A.1.

## Conclusion

In the last years an increasing number of studies on the degradation of materials produced since the beginning of the 1900s has been published, but we still know very little on their use in aerospace technology^2,3^. The study of the materials used in the Pioneer Era of aviation is still an uncharted territory, also because a vast portion of surviving planes has been partially or completely restored, usually by total replacement of the fabric. This makes the study of the early aircrafts extremely difficult and their conservation and preservation precarious.

The chemical characterization carried out on the Ansaldo A.1 (1918) showed a pioneering combination of traditional natural organic materials—wood, silk, linseed oil, pine pitch, camphor—together with synthetic organic pigments—PR3 (1904) and PR49 (1899) —and an early synthetic polymer—cellulose acetate—whose use as a dope started in 1914 in the UK. This plane can be considered the state of the art of aerospace technology at the beginning of the 1900s, produced with the best end most expensive materials available at the time. Unfortunately, this choice of material had an unfavorable effect on the aircraft's stability over time. The combined characteristics of acetate polymers and silk caused a series of unfortunate degradation processes, resulting in a slow decay of the structural integrity of the biplane, which severely worsened during the lockdown enforced by the Italian government during the COVID-19 pandemic.

This study represents one of the first steps into the material study of early historical aircrafts and highlighted the potential of *vinegar syndrome* as a threat not only to acetate-based materials, such as films and photographs, but also to larger objects, such as the Ansaldo A1 aircraft. Furthermore, it confirmed the significance of museum conditions, including humidity, temperature, and ventilation, in relation to this conservation issue and its potential impact on large-scale objects.

The analytical evidence obtained on the silk and dope layer has proved fundamental in determining the most suitable conservation approach for the aircraft and is assisting the ongoing conservation campaign. The conservation entails, besides a series of interventions focused on improving the overall appearance of the aircraft, the adoption of measures to mitigate the acidic pH conditions of the silk to control and slow down the *vinegar syndrome*, i.e., the autocatalytic hydrolysis of cellulose acetate. A detailed report of the conservation campaign will be published upon the completion of the work.

The characterization of aircrafts as multi-material objects is a new and compelling avenue of research in the field of conservation, which is needed to extend the lifetime of these objects and ensure that these silent witnesses of the history of chemistry and aircraft technology can pass down their testimony to future generations.

## Materials and methods

### Infrared spectroscopy analysis (ATR-FTIR)

The ATR-FTIR spectroscopy were performed using a Nicolet™ iN10 MX system (Thermo Scientific, USA) with a germanium crystal (penetration 066 µm at 1000 cm^−1^). The spectra were acquired in the range 650–400 cm^−1^ with a resolution of 4 cm^−1^ on an area of 200 × 200 µm.

### Field emission gun scanning electron microscope (FEG-SEM)

FEG-SEM analysis were performed using a Quanta 450 FEG field emission gun—scanning electron microscope (FEI Company, USA) equipped with a QUANTAX XFlash Detector 6|10 for microanalyses (Bruker, USA) and X-ray compositional mapping and a QUANTAX EBSD/EDS analysis system for phase identification and textural mapping (Bruker).

### Analytical pyrolysis coupled with gas chromatography and mass spectrometry (Py-GC–MS)

Py-GC–MS analyses were performed using a multi-shot pyrolyzer EGA/PY-3030D (Frontier Lab, Japan) coupled with an 8890 gas chromatograph, combined with a 5977B mass selective single quadrupole mass spectrometer detector (Agilent Technologies, USA). The complete instrumental conditions used to perform the analysis of the samples are reported in the Supporting information (S1).

### Analysis of the volatile organic compounds

The collection of the volatile organic compounds was performed using a Magic Chemisorber® device with a polydimethylsiloxane stationary phase (500 μm thickness). The device was used for 6 h of passive exposure in the museum environment. The analyses were performed using a multi-shot pyrolyzer EGA/PY-3030D (Frontier Lab, Japan) coupled with an 8890 gas chromatograph, combined with a 5977B mass selective single quadrupole mass spectrometer detector (Agilent Technologies, USA). The complete instrumental conditions used to perform the analysis of the samples are reported in the Supporting information (S1).

### Analysis of lipid materials

The analyses of the lipid materials in the samples were carried out on a 1200 Infinity HPLC coupled by a Jet Stream ESI interface with a Quadrupole-Time of Flight tandem mass spectrometer 6530 Infinity Q-ToF (Agilent Technologies). The sample pretreatment of the samples was performed using an Ethos One microwaves oven system (Milestone Srl, Italy). The complete instrumental conditions used to perform the analysis of the samples are reported in the Supporting information (S1).

### Analysis of organic dyes and pigments

For the HPLC–DAD analyses, the chromatographic system consisted of a PU-2089 quaternary pump equipped with a degasser, an AS-950 autosampler, and an MD-2010 spectrophotometric diode array detector (Jasco International Co., Japan). For the HPLC–MS analysis the system consisted of HPLC 1200 Infinity, coupled with a quadrupole-time of flight mass spectrometer Infinity Q-ToF 6530 detector by a Jet Stream ESI interface (Agilent Technologies). The complete instrumental conditions used to perform the analysis of the samples are reported in the Supporting information (S1).

## Supplementary Information

Supplementary Information.

## Data Availability

The datasets used and/or analyzed during the current study are available from the corresponding author on reasonable request.
