# Supplementary material for: The role of early synthetic materials degradation in the downfall of the Ansaldo A.1, an Italian World War I biplane fighter
Source: Sci Rep. 2023 Jul 27;13:12170. doi: 10.1038/s41598-023-39164-9 (PMC10374885; doi:10.1038/s41598-023-39164-9)
Supplement: Supplementary file 1 — Supplementary Information. [file 41598_2023_39164_MOESM1_ESM.pdf]

# The emergency landing of Ansaldo A.1 Balilla (1918) fighter aircraft

Jacopo La Nasa[1,2], Alessio Ceccarini[1,2], Riccardo Ducoli[1], Antonella Manariti[1,2], Jeanette J. Lucejko[1,2], Ilaria Degano[1,2], Neva Capra[3], Lucia Giovannini[3], Maria Luisa Tomasi[3], Francesca Modugno[1,2], Maria Perla Colombini[1], Ilaria Bonaduce[1,2]

[1] Department of Chemistry and Industrial Chemistry, Via Giuseppe Moruzzi 13, 56124, Pisa, Italy

[2] CISUP Centre for Instrument Sharing, University of Pisa, Pisa, Italy

[3] Soprintendenza per i beni culturali della provincia autonoma di Trento, Via San Marco 27, 38122 Trento, Italy

## Supporting material

S1. Methods

S2. Cross sections

S3. Py-GC-MS chromatograms

S4. ATR-FTIR spectra

S5. HPLC-ESI-Q-ToF extracted ion chromatograms for the lipid materials

S6. HPLC-ESI-Q-ToF and HPLC-DAD results for the dyes and organic pigments

S7. VOCs analysis sampling positions and results

S8. Py-GC-MS chromatograms and sampling positions of the wood samples

S9. FEG-SEM data

## S1. Methods

### Infrared spectroscopy analysis (ATR-FTIR)

The ATR-FTIR spectroscopy were performed using a Nicolet™ iN10 MX system (Thermo Scientific, US A) with a germanium crystal (penetration 0,66 µm at 1000 cm-1). The spectra were acquired in the range 650-400 cm-1 with a resolution of 4 cm-1 on an area of 200 x 200 µm.

### Field emission gun scanning electron microscope (FEG-SEM)

FEG-SEM analysis were performed using a Quanta 450 FEG field emission gun – scanning electron microscope (FEI Company, USA) equipped with a QUANTAX XFlash Detector 6|10 for microanalyses (Bruker, USA) and X-ray compositional mapping and a QUANTAX EBSD/EDS analysis system for phase identification and textural mapping (Bruker).

### Analytica pyrolysis coupled with gas chromatography and mass spectrometry (Py-GC-MS)

The analyses were performed using a multi-shot pyrolyzer EGA/PY-3030D (Frontier Lab, Japan) coupled with an 8890 gas chromatograph, combined with a 5977B mass selective single quadrupole mass spectrometer detector (Agilent Technologies, US).

The pyrolysis furnace used in the method to characterize the synthetic polymers was set at 600 °C while the Py-GC interface was set at 280 °C (Py time 0.2 min) (*J. La Nasa et al., Historical aircraft paints: Analytical pyrolysis for the identification of paint binders used on two Messerschmitt Bf 109 planes. Journal of Analytical and Applied Pyrolysis 163, 105468 (2022).*

For the analysis of the natural organic materials performed using hexamethyldisilazane (HMDS) as derivatizing agents the furnace was set at 550 °C while the Py-GC interface was set at 280 °C (Py time 0.2 min)(*2, 3*). 2 µL of HMDS were added in the cup before the pyrolysis.

For both the approaches the pyrolysis products were separated with an HP-5MS capillary column (30 m x 0.25 mm, film thickness 0.25 µm, Agilent Technologies, USA), while the GC injector was operated in split mode at 280 °C and with a 20:1 ratio.

The GC oven temperature program for the synthetic polymers was as follows: 40 °C for 5 min, 10 °C/min to 310 °C for 20 min. The GC oven temperature program for the lipids was as follows: 32 °C for 10 min, 10 °C/min to 310 °C for 20 min. For both methods, the mass spectrometric acquisition was in the range m/z 35-600. The weights of sample introduced in the pyrolysis chamber were measured with an XS3DU microanalytical scale (Mettler-Toledo, USA).

## Analysis of triacylglycerols

For the HPLC analysis of TAGs, ~0.1 mg of sample was subjected to extraction assisted by microwaves in a Ethos One oven (Milestone, U.S.A.) ,600 W, with 300 µL of a chloroform-hexane mixture (3:2, 80°C, 25 min). The extracts were dried under a nitrogen stream, diluted with 600 µL of elution mixture, and filtered on a 0.45 µm PTFE filter (Grace Davison Discovery Sciences, U.S.A.) just before injection. Analyses were carried out on a 1200 Infinity HPLC coupled by a Jet Stream ESI interface with a Quadrupole-Time of Flight tandem mass spectrometer 6530 Infinity Q-ToF (Agilent Technologies, USA). The separation was performed on an Agilent Poroshell 120 EC-C18 column (3.0 mm × 50 mm, 2.7 µm) with a Zorbax eclipse plus C-18 guard column (4.6 mm×12.5 mm, 5 µm). Injection volume: 2 µL. Column temperature: 45°C. The instrumental parameters for the chromatographic separation and the mass spectrometric detection and identification of the TAGs are reported along with the figures of merit of the methods in (*J. La Nasa et al., Novel application of liquid chromatography/mass spectrometry for the characterization of drying oils in art: Elucidation on the composition of original paint materials used by Edvard Munch (1863–1944). Analytica Chimica Acta 896, 177-189 (2015)*).

## Analysis of organic dyes and pigments

The sample pretreatment for the analysis of organic dyes and pigments used prior HPLC-DAD and HPLC-ESI-Q-ToF analysis entailed the addition of 300 µL of dimethyl sulfoxide to 0.2 mg of sample, extraction at 60 °C for 10 min in an ultrasonic bath and filtration using 0.45 µm PTFE filter. For the HPLC-DAD analysis a PU-2089 Quaternary Pump with degasser, equipped with an autosampler AS-950 coupled to a spectrophotometric diode array detector MD-2010 (all modules are from Jasco International Co, Japan) was used. The data were processed with ChromNav software. The same HPLC-ESI-Q-ToF system, analytical column and guard column used for the analysis of triglycerides were used. The column temperature was 30 °C and the injection volume was 10 µL and 4 µL for HPLC-DAD and HPLC-ESI-Q-ToF, respectively. Separation gradient: FA 0.1% v/v in H2O (eluent A) and FA 0.1% v/v in $CH_3CN$ (eluent B). The detailed instrumental parameter for the chromatographic separation and the mass spectrometric detection and identification of the dyes are reported in (*J. La Nasa et al., Synthetic materials in art: a new comprehensive approach for the characterization of multi-material artworks by analytical pyrolysis. Heritage Science 7, 8 (2019)*).

## Analysis of the volatile organic compounds

The analyses were performed using a multi-shot pyrolyzer EGA/PY-3030D (Frontier Laboratories, Japan) coupled with an 8890 gas chromatograph, combined with a 5977B mass selective single quadrupole mass spectrometer detector (Agilent Technologies, US). The pyrolyzer system was equipped with a quick stabilizer pressure control QSP-1046E (Frontier Lab), and a Micro jet Cryo-Trap MJT-1035E (Frontier Lab). The cryo-focusing time was automatically controlled by the instrument software. The Chemisorbers were desorbed in the pyrolysis system at 280°C. The desorbed compounds were cryo-trapped with $N_2$ at -195 °C.

The chromatographic separation of the pyrolysis products was performed on a fused silica capillary column HP-5MS UI (5% diphenyl-95% dimethyl-polysiloxane, 30 m x 0.25 mm i.d., 0.25 µm film thickness, J&W Scientific, Agilent Technologies), preceded by 2 m of deactivated fused silica pre-column with an internal diameter of 0.32 mm. The chromatographic conditions for the analysis were 35 °C held for 6 min, 20 °C/min to 310 °C held for 40 min. The helium (99.9995% purity) gas flow was set in constant flow mode at 1.0 mL/min. The mass spectrometer was operated in EI positive mode (70 eV, scanning m/z 35-700).

## pH measurements

200 mg of virgin reference silk and two silk samples with dope from the plane were left immersed in 5 ml of a 100 mM NaCl solution for 12 hours. The pH measurement was carried out with a glass electrode after calibration with a standard solution at pH 4 and another at pH 10.

## S2. Cross sections

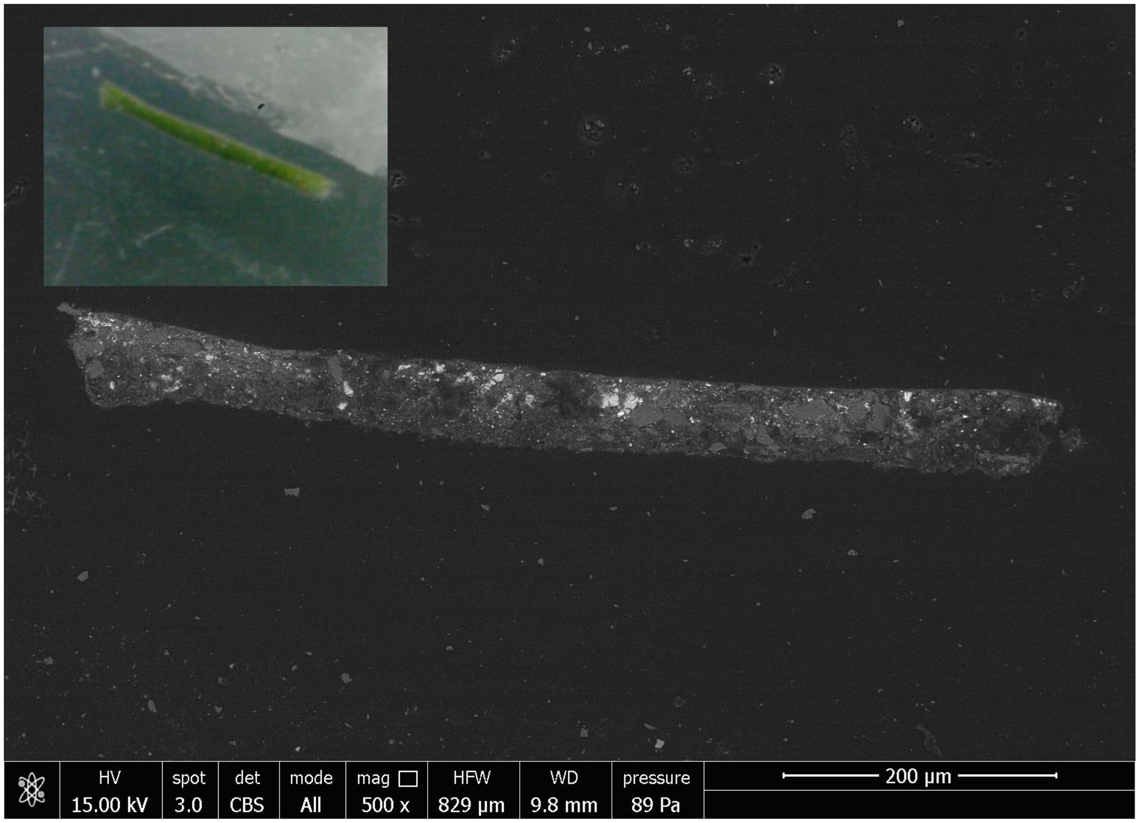


**Figure S.1** – Cross section obtained for sample 2

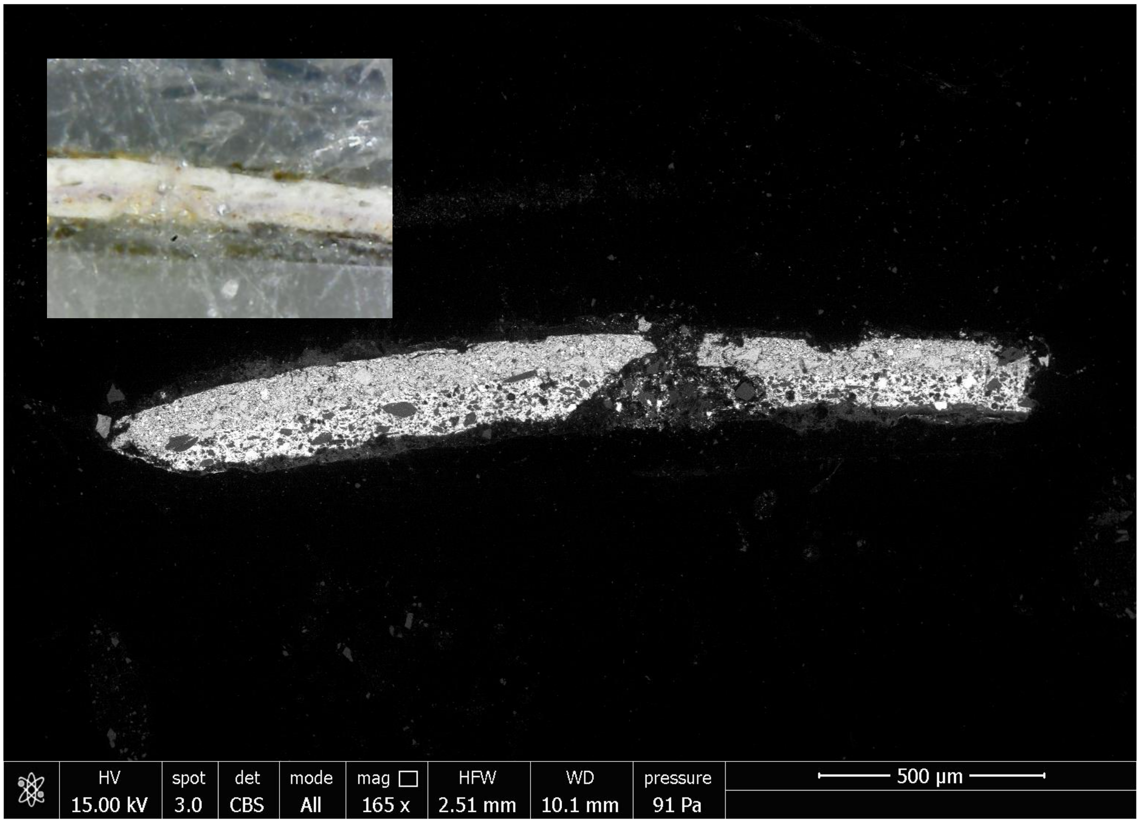


**Figure S.2** – Cross section obtained for sample 6

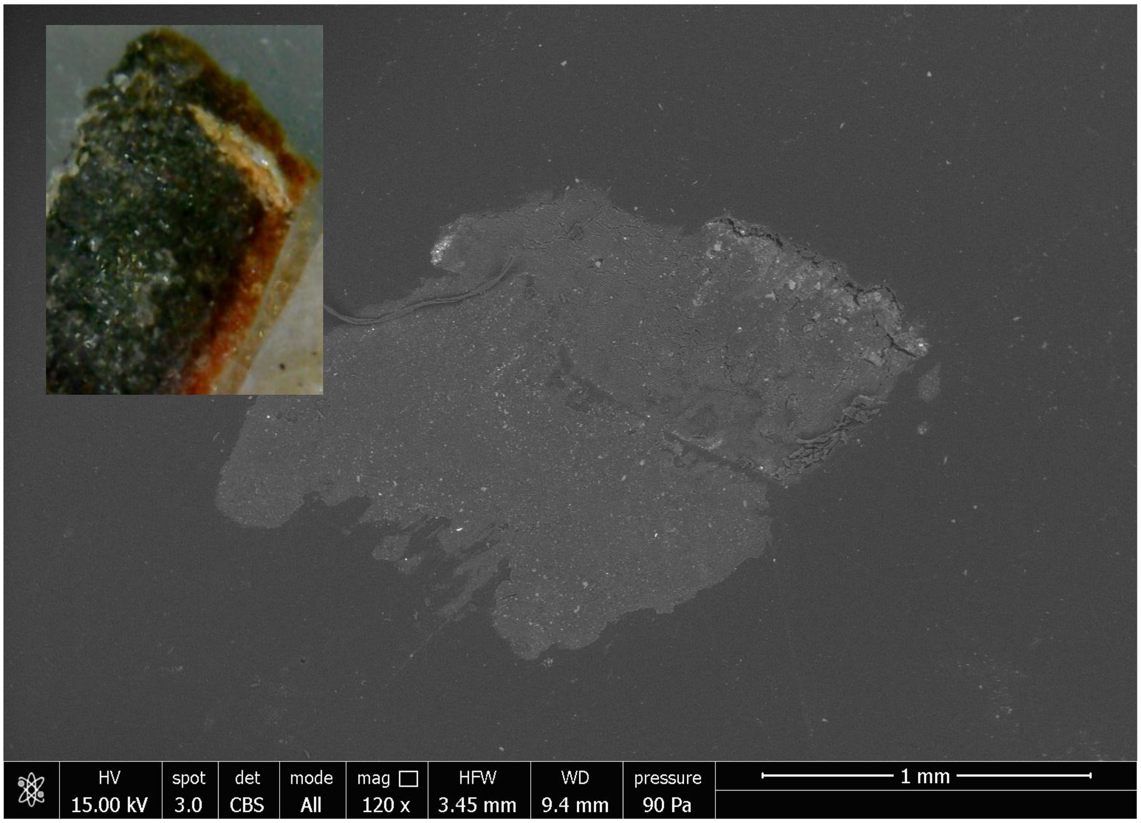

**Figure S.3** – Cross section obtained for sample 7

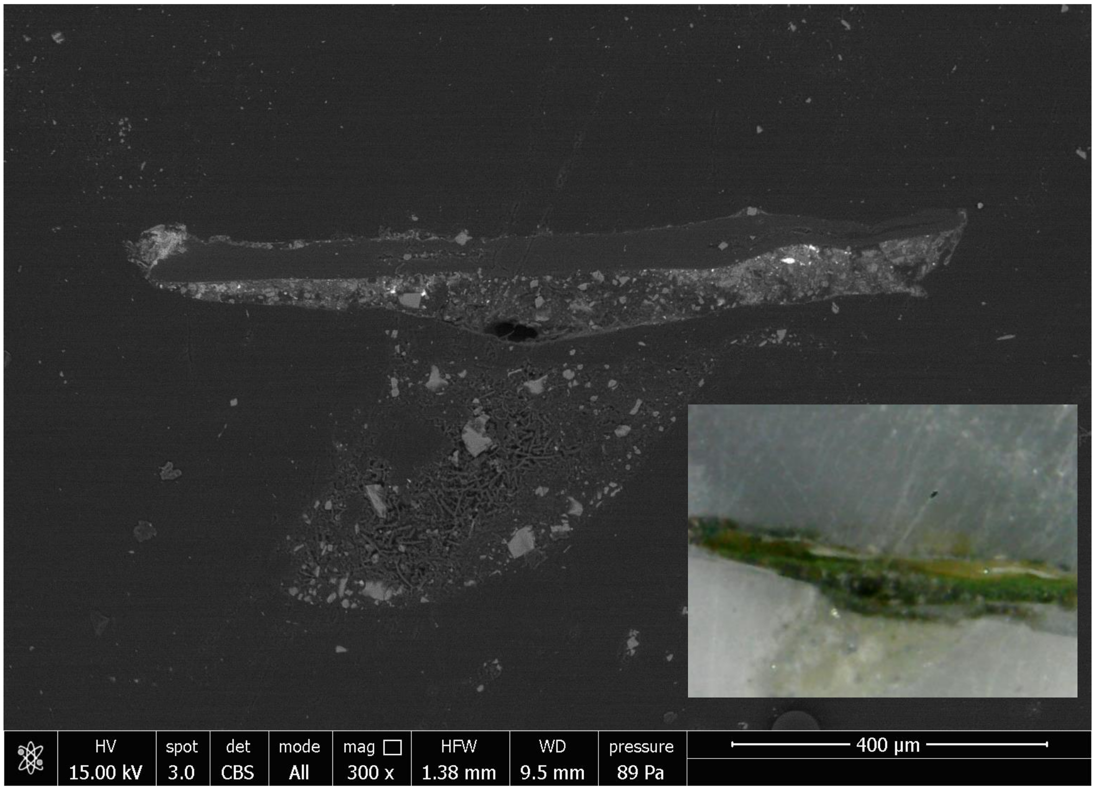


**Figure S.4** – Cross section obtained for sample 9

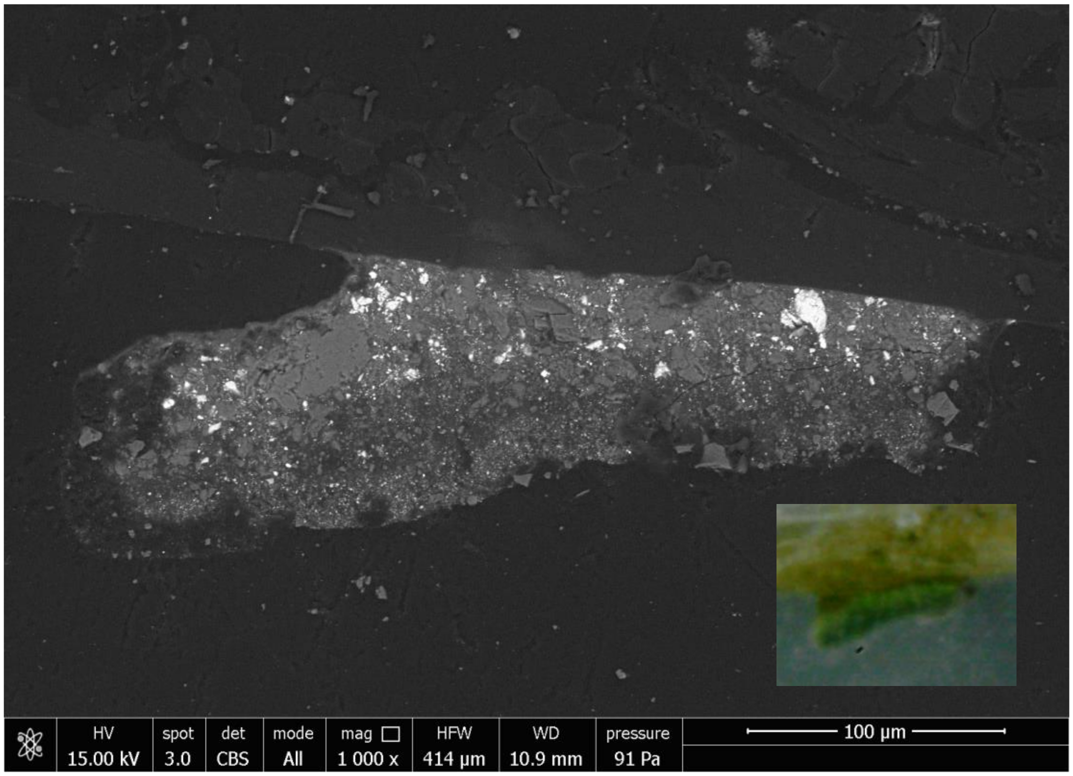


**Figure S.5** – Cross section obtained for sample 11

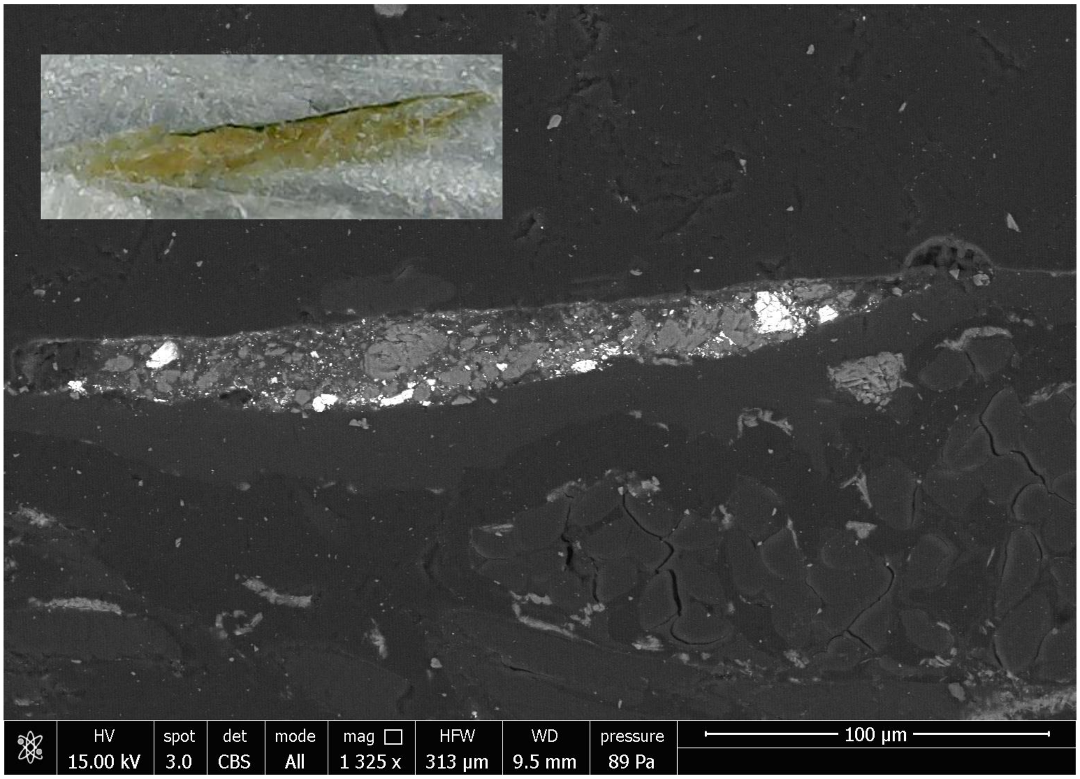


**Figure S.6** – Cross section obtained for sample 13

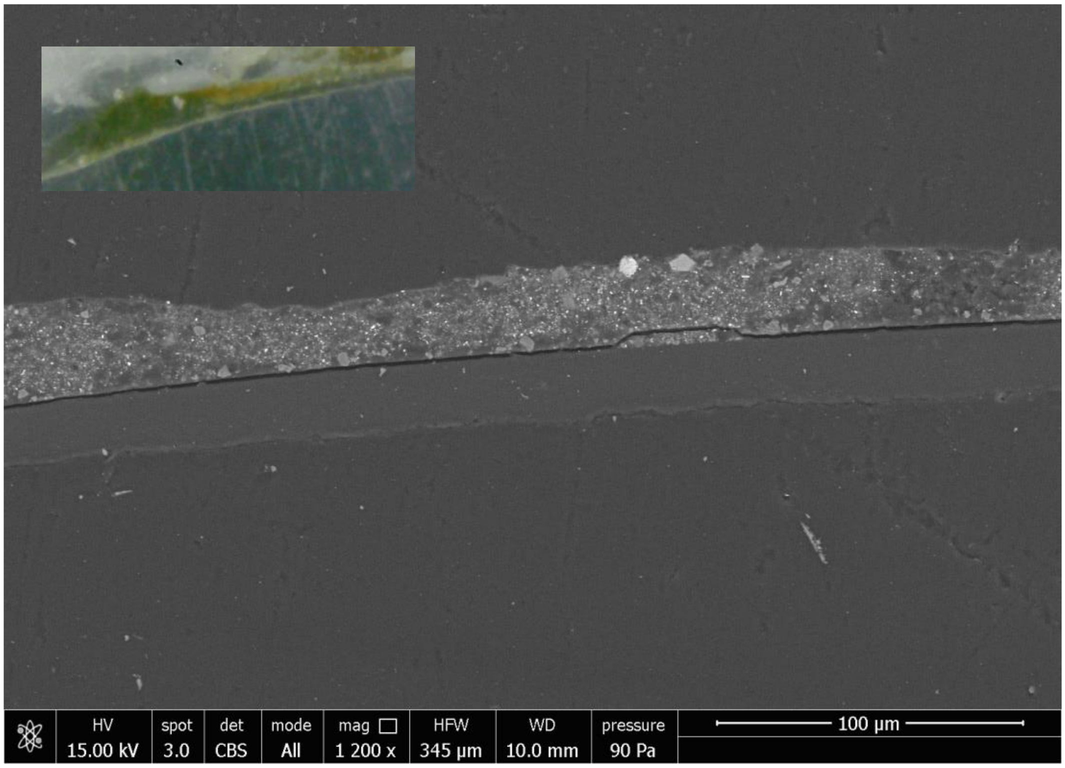


**Figure S.7** – Cross section obtained for sample 14

## S3. Py-GC-MS chromatograms

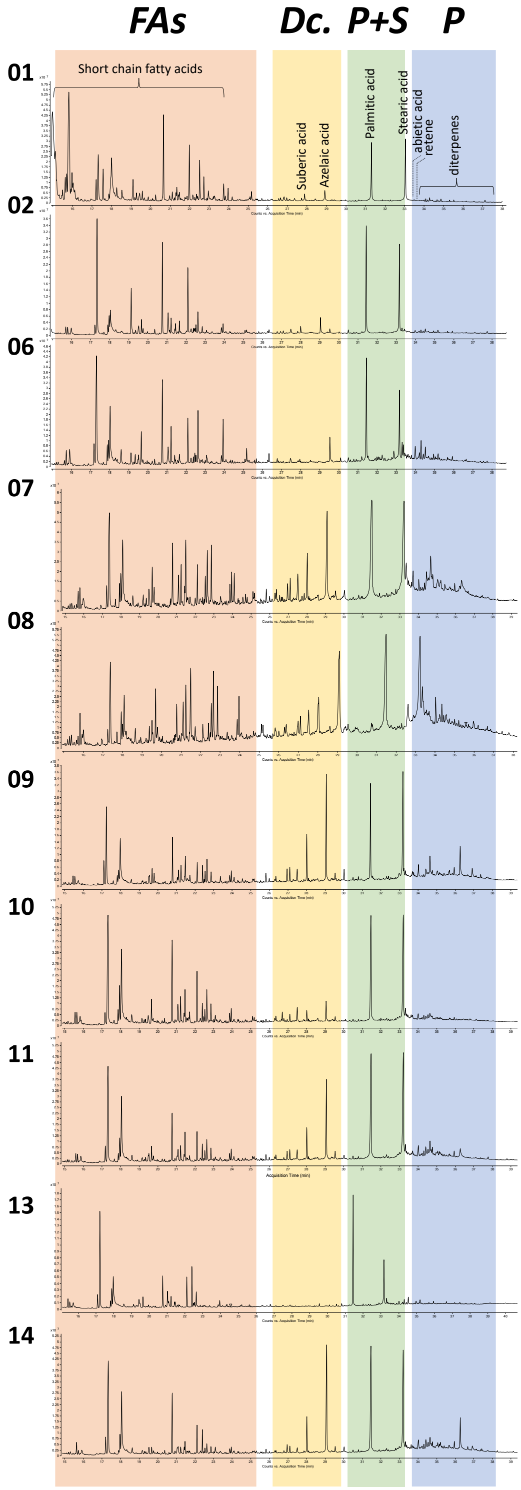


**Figure S.8**- Py-GC-MS chromatograms obtained for the different samples using on-line derivatization (HMDS); FAs: fatty acids from the polymeric network, Dc.: dicarboxylic acids, P+S: palmitic and stearic acids, P: diterpenes from the pine pitch

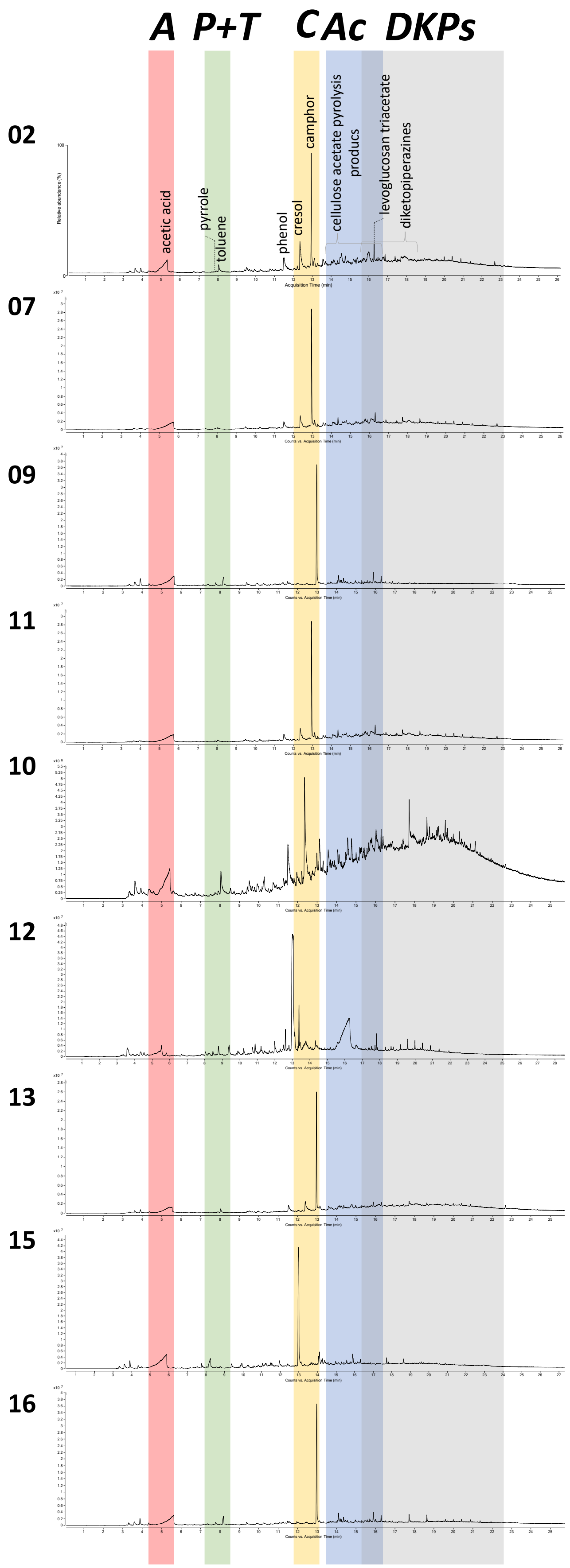
A
P+T
C
Ac
DKPs
02
07
09
11
10
12
13
15
16
acetic acid
pyrrole
toluene
phenol
cresol
camphor
cellulose acetate pyrolysis produсs
levoglucosan triacetate
diketopiperazines

**Figure S.9**- Py-GC-MS chromatograms obtained for the different samples without derivatization; A: acetic acid, P+T: pyrrole and toluene, Ac: cellulose acetate pyrolysis products, DKPs: diketopiperazines

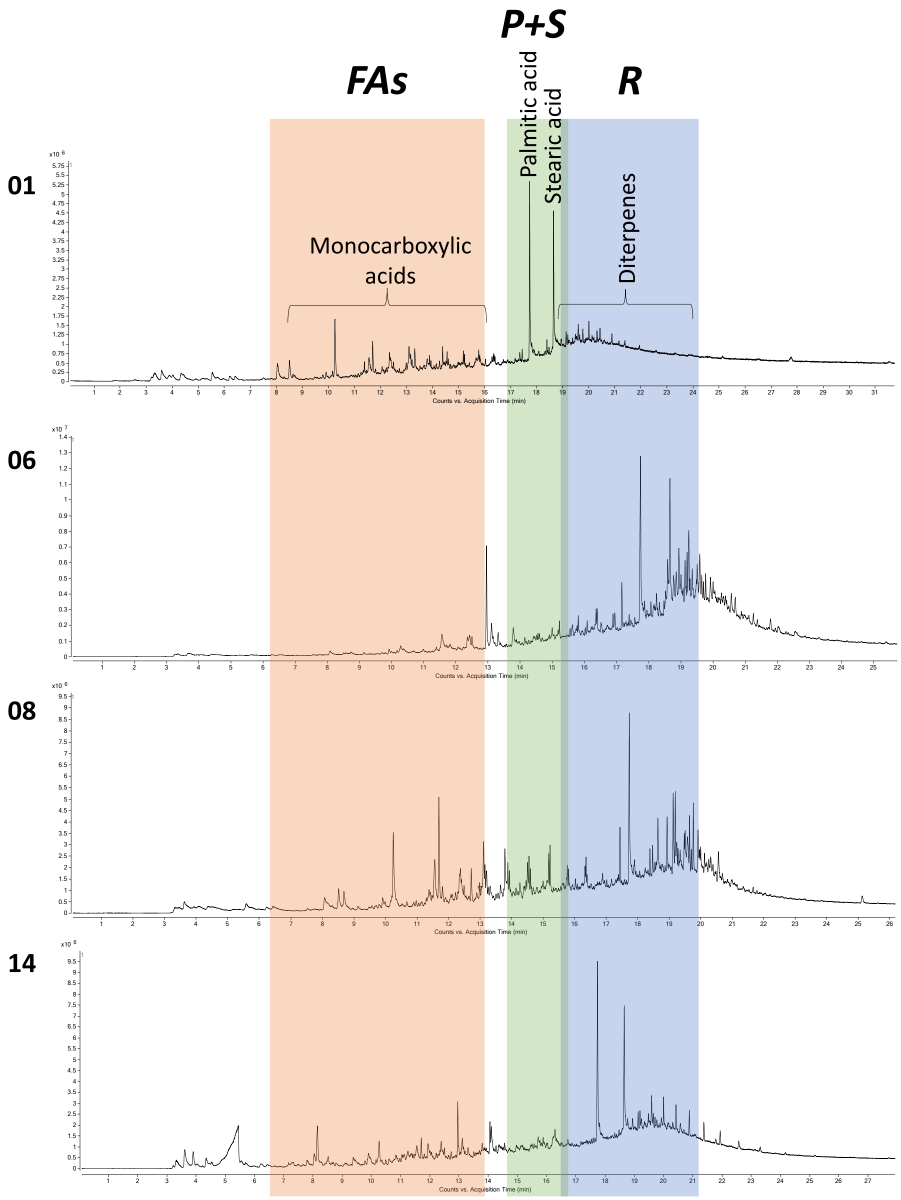


**Figure S.10**- Py-GC-MS chromatograms obtained for the different samples without derivatization; FAs: fatty acids from the polymeric network, P+S: palmitic and stearic acids, R: diterpenes from the pine pitch

## S4. ATR-FTIR spectra

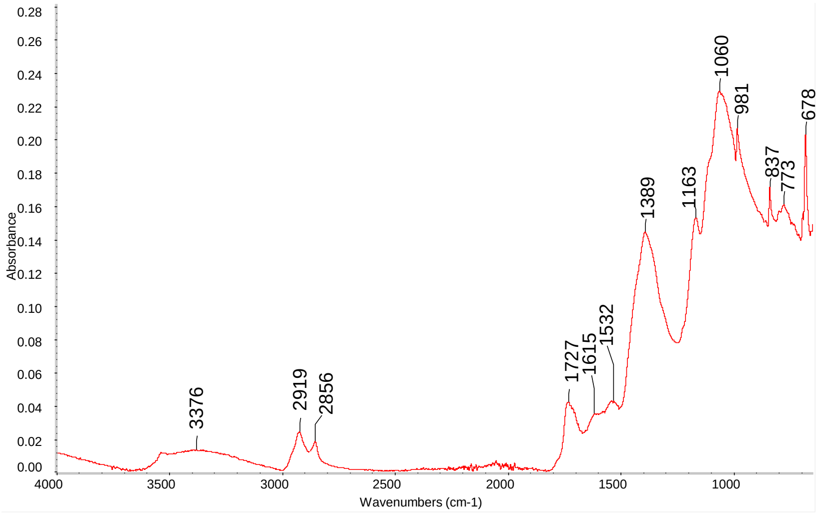


**Figure S.11**- ATR-FTIR spectrum acquired for samples 01

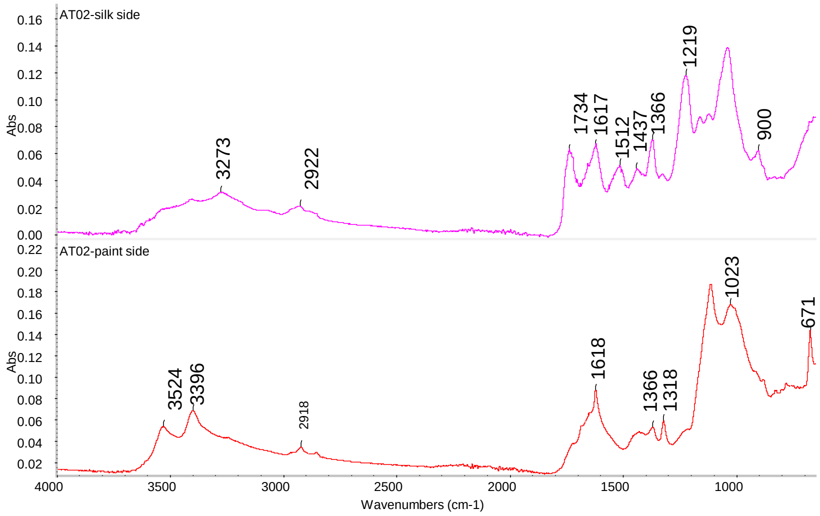


**Figure S.12**- ATR-FTIR spectrum acquired for samples 02 (silk side, above; paint side, below)

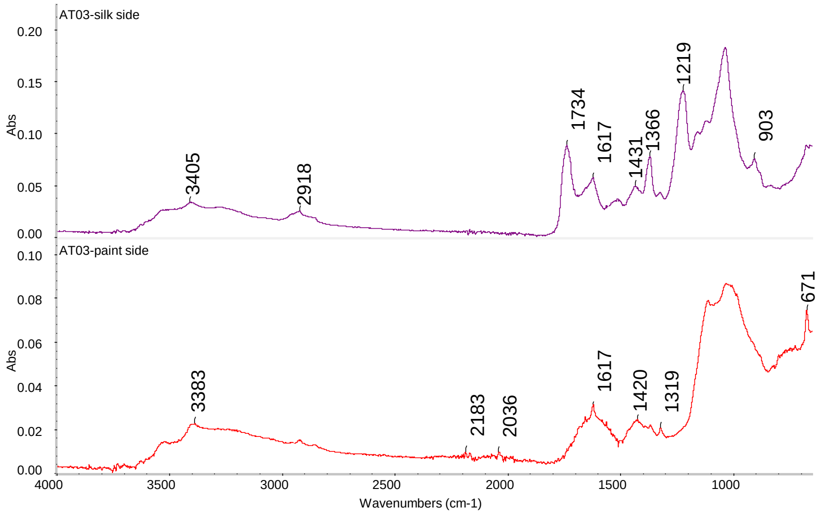


**Figure S.13**- ATR-FTIR spectrum acquired for samples 03 (silk side, above; paint side, below)

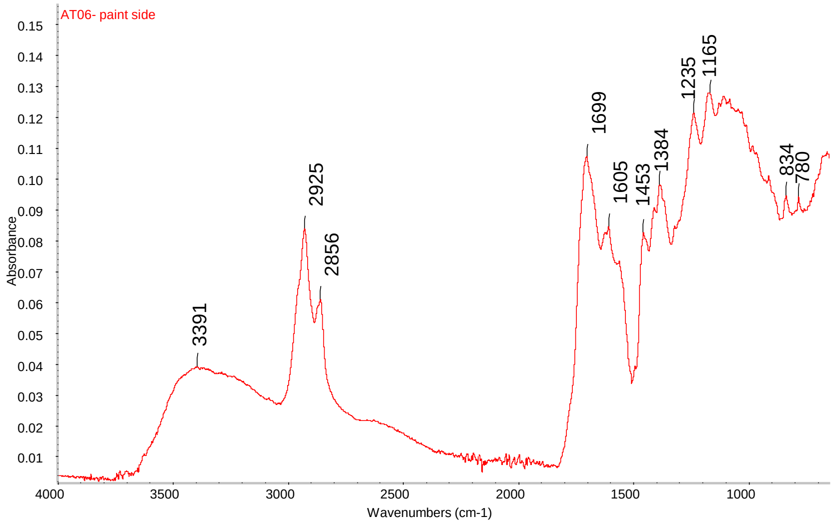


**Figure S.14**- ATR-FTIR spectrum acquired for samples 05

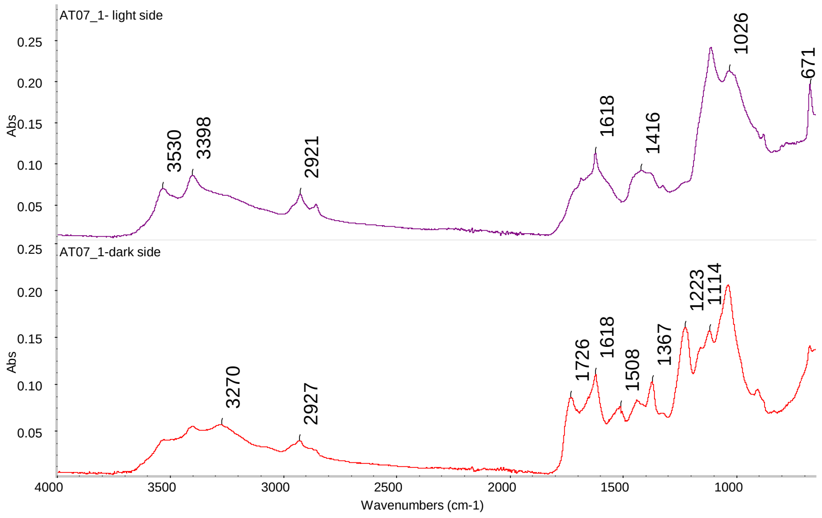


**Figure S.15**- ATR-FTIR spectrum acquired for samples 07 (light side, above; dark side, below)

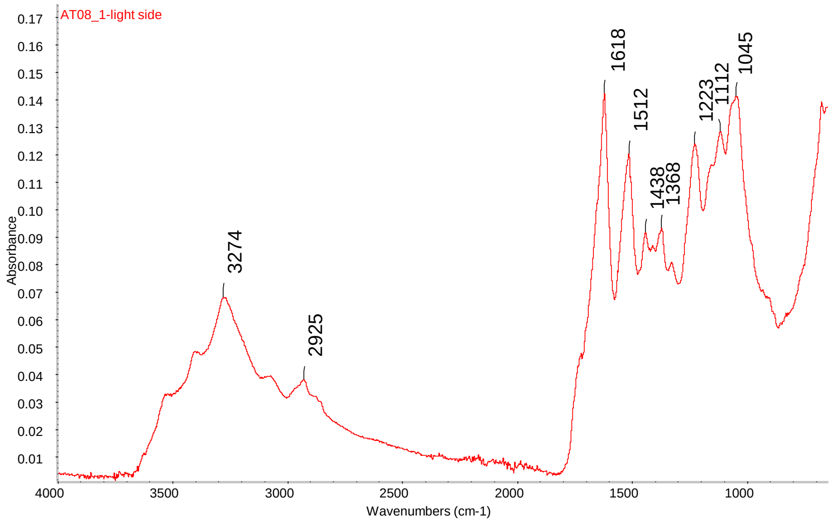


**Figure S.16**- ATR-FTIR spectrum acquired for samples 08

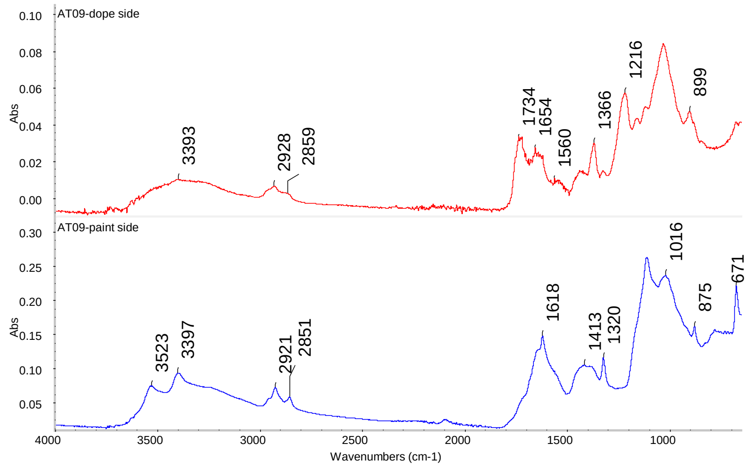


**Figure S.17**- ATR-FTIR spectrum acquired for samples 09 (dope side, above; paint side, below)

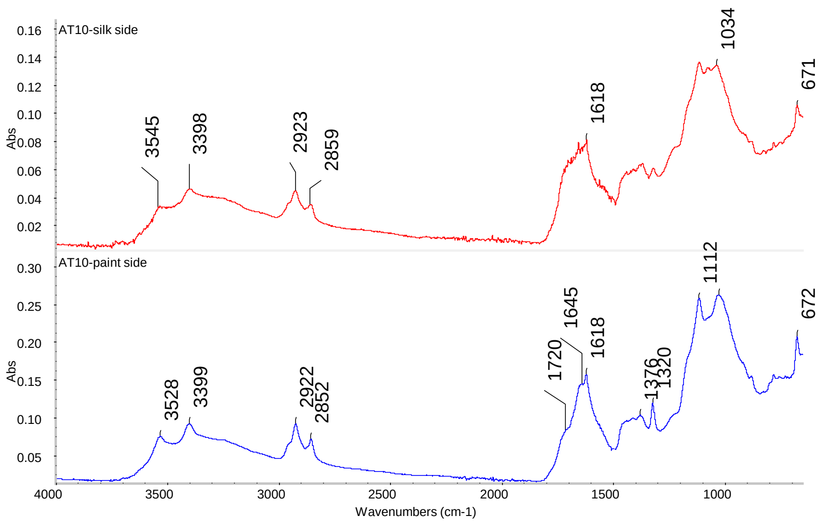


**Figure S.18**- ATR-FTIR spectrum acquired for samples 10 (silk side, above; paint side, below)

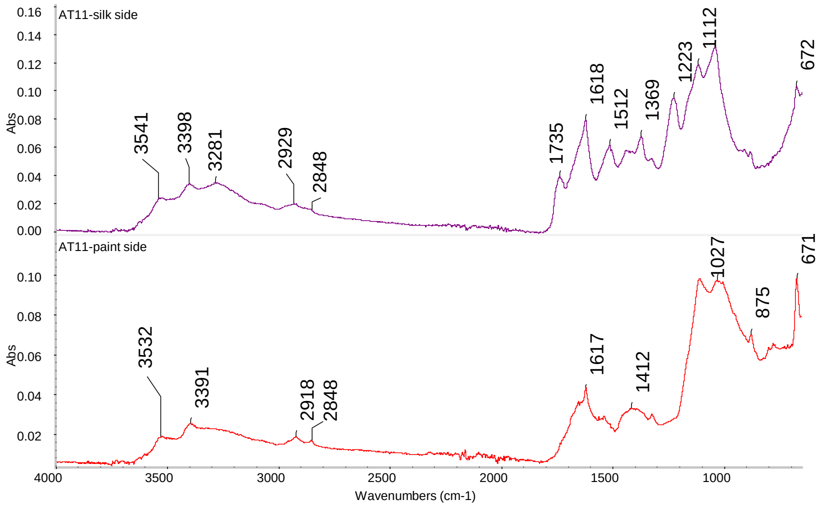


**Figure S.19**- ATR-FTIR spectrum acquired for samples 11 (silk side, above; paint side, below)

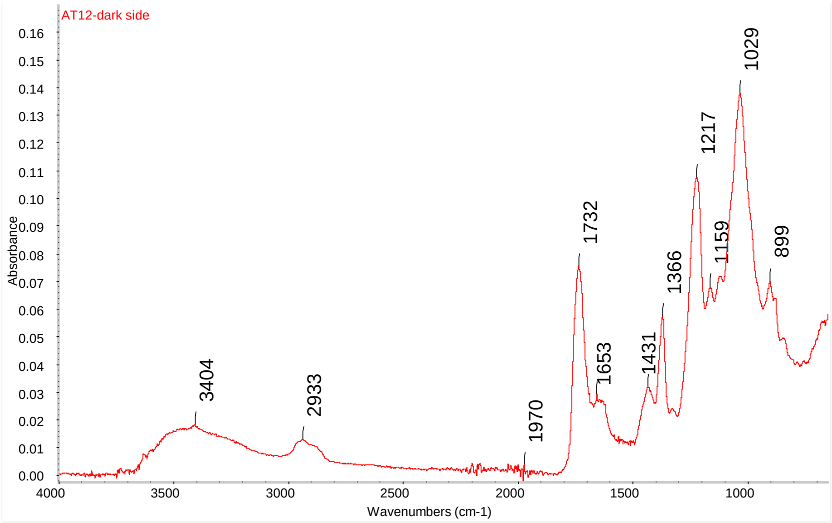


Figure S.20- ATR-FTIR spectrum acquired for samples 12

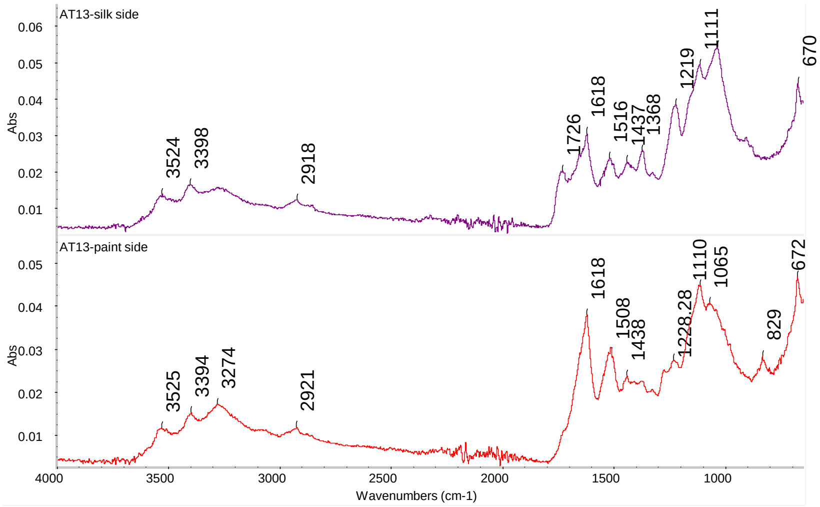


Figure S.20- ATR-FTIR spectrum acquired for samples 13

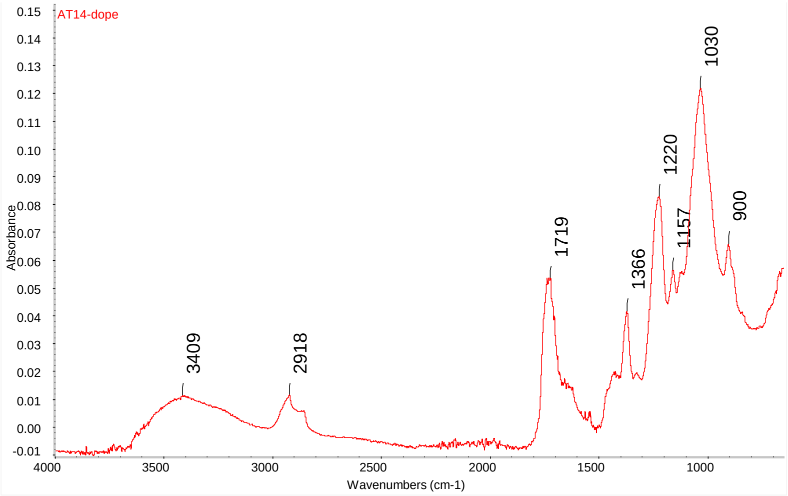


Figure S.21- ATR-FTIR spectrum acquired for samples 14

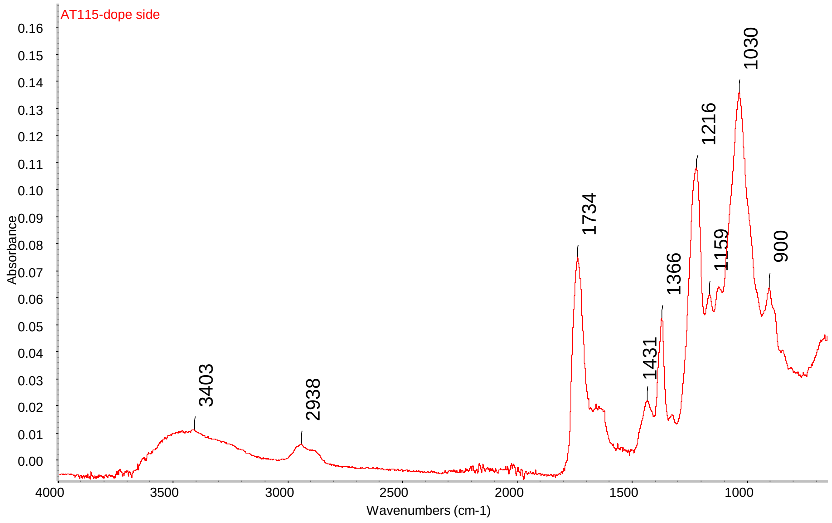


**Figure S.22**- ATR-FTIR spectrum acquired for samples 15

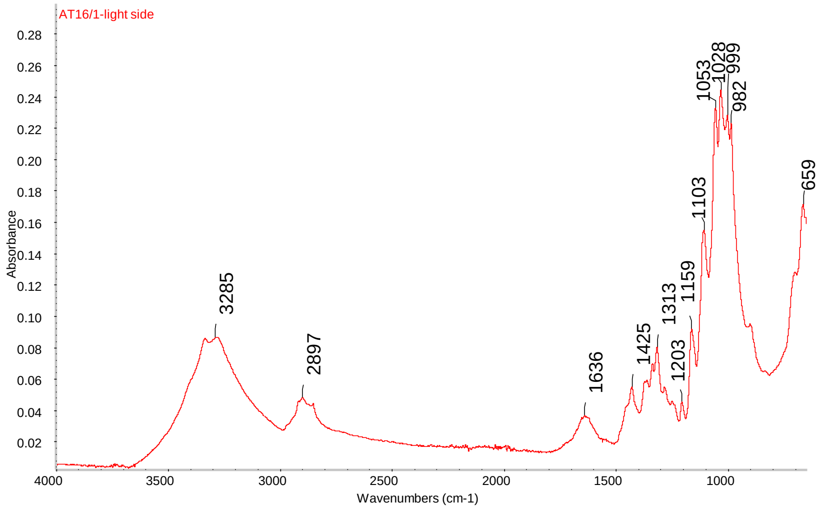


**Figure S.23**- ATR-FTIR spectrum acquired for samples 16

## S5. HPLC-ESI-Q-ToF extracted ion chromatograms for the lipid materials

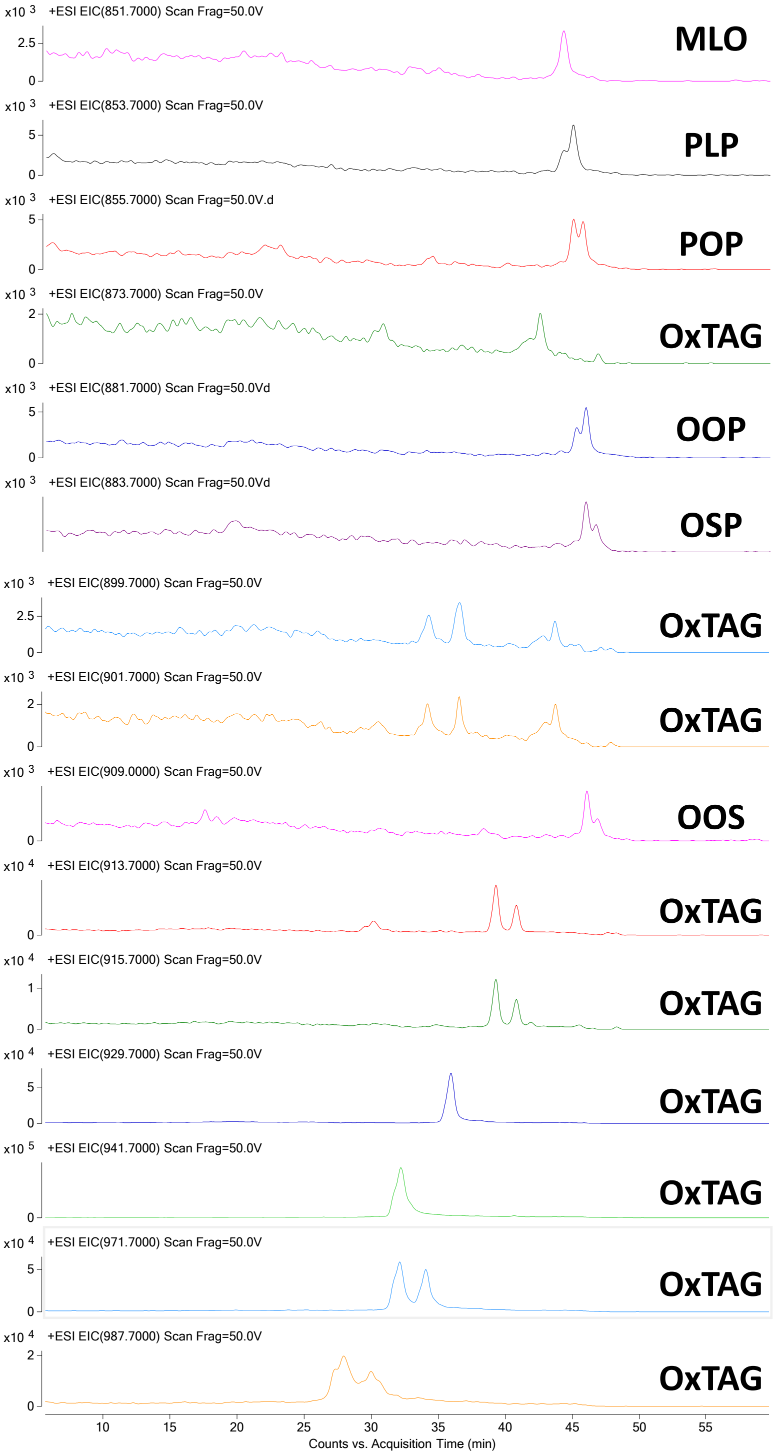


**Figure S.24** - HPLC-ESI-Q-ToF extract ion chromatograms of the of the extracts of sample 03. Abbreviation list: M: myristic acid, P: palmitic acid, L: linoleic acid; O: oleic acid, S:stearic acid; OxTAGs: m/z 897.7 ($C_{18:1,1OH}$OP, $[M+Na]^+$), m/z 899.7 ($C_{18:1,1OH}$SP, $[M+Na]^+$), m/z 901 ($C_{18,1OH}$SP, $[M+Na]^+$), m/z 913.7 ($C_{18:1,1OH}$ $C_{18:1,1OH}$P, $[M+Na]^+$), m/z 915.7 ($C_{18,1OH}$ $C_{18:1,1OH}$P, $[M+Na]^+$), m/z 929.7 ($C_{18:1,2OH}$ $C_{18:1,1OH}$P, $[M+Na]^+$), m/z 941.7 ($C_{18:1,1OH}$ $C_{18:1,1OH}$S, $[M+Na]^+$), m/z 971.7 ($C_{18:1,2OH}$ $C_{18:1,1OH}$ $C_{18:1,1OH}$, $[M+Na]^+$), m/z 987.7 ($C_{18:1,2OH}$ $C_{18:1,2OH}$ $C_{18:1,1OH}$, $[M+Na]^+$)

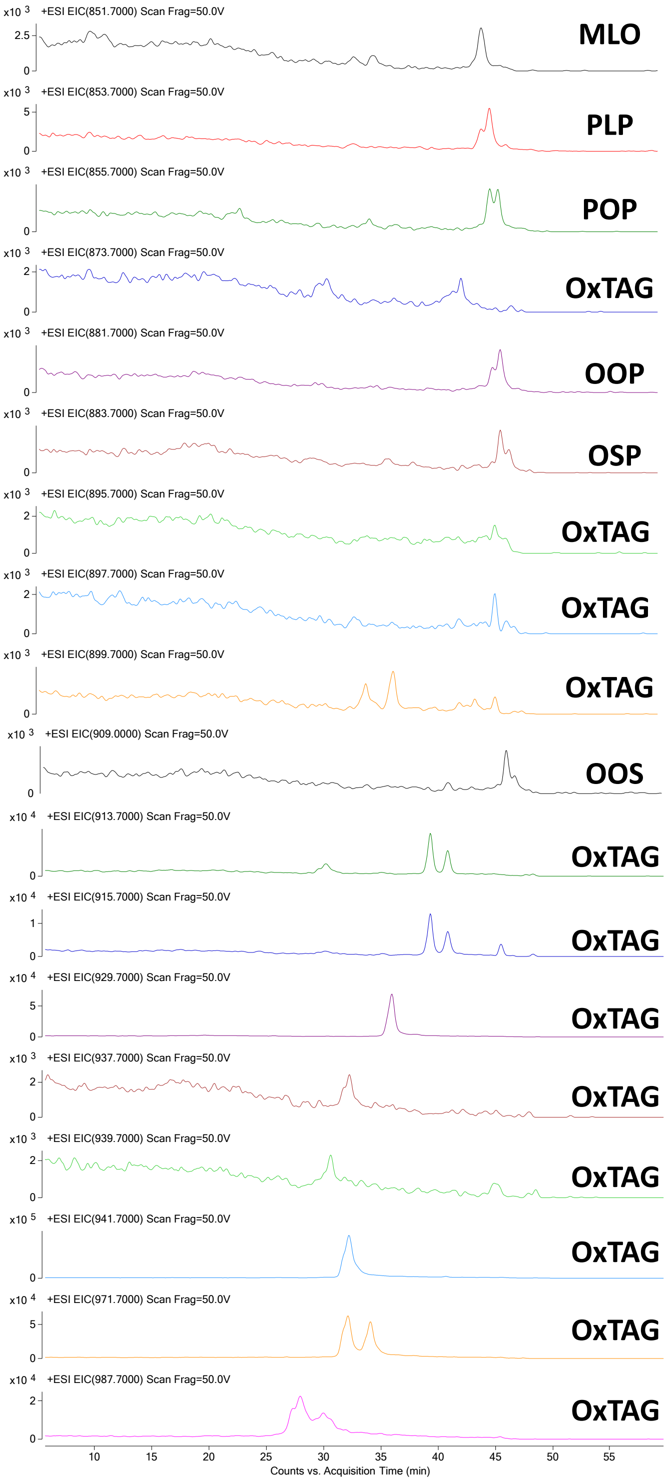


**Figure S.25** - HPLC-ESI-Q-ToF extract ion chromatograms of the of the extracts of sample 06. Abbreviation list: M: myristic acid, P: palmitic acid, L: linoleic acid; O: oleic acid, S:stearic acid; OxTAGs: m/z 897.7 ($C_{18:1,1OH}$OP, $[M+Na]^+$), m/z 899.7 ($C_{18:1,1OH}$SP, $[M+Na]^+$), m/z 901 ($C_{18,1OH}$SP, $[M+Na]^+$), m/z 913.7 ($C_{18:1,1OH}$ $C_{18:1,1OH}$P, $[M+Na]^+$), m/z 915.7 ($C_{18,1OH}$ $C_{18:1,1OH}$P, $[M+Na]^+$), m/z 929.7 ($C_{18:1,2OH}$ $C_{18:1,1OH}$P, $[M+Na]^+$), m/z 941.7 ($C_{18:1,1OH}$ $C_{18:1,1OH}$S, $[M+Na]^+$), m/z 971.7 ($C_{18:1,2OH}$ $C_{18:1,1OH}$ $C_{18:1,1OH}$, $[M+Na]^+$), m/z 987.7 ($C_{18:1,2OH}$ $C_{18:1,2OH}$ $C_{18:1,1OH}$, $[M+Na]^+$)

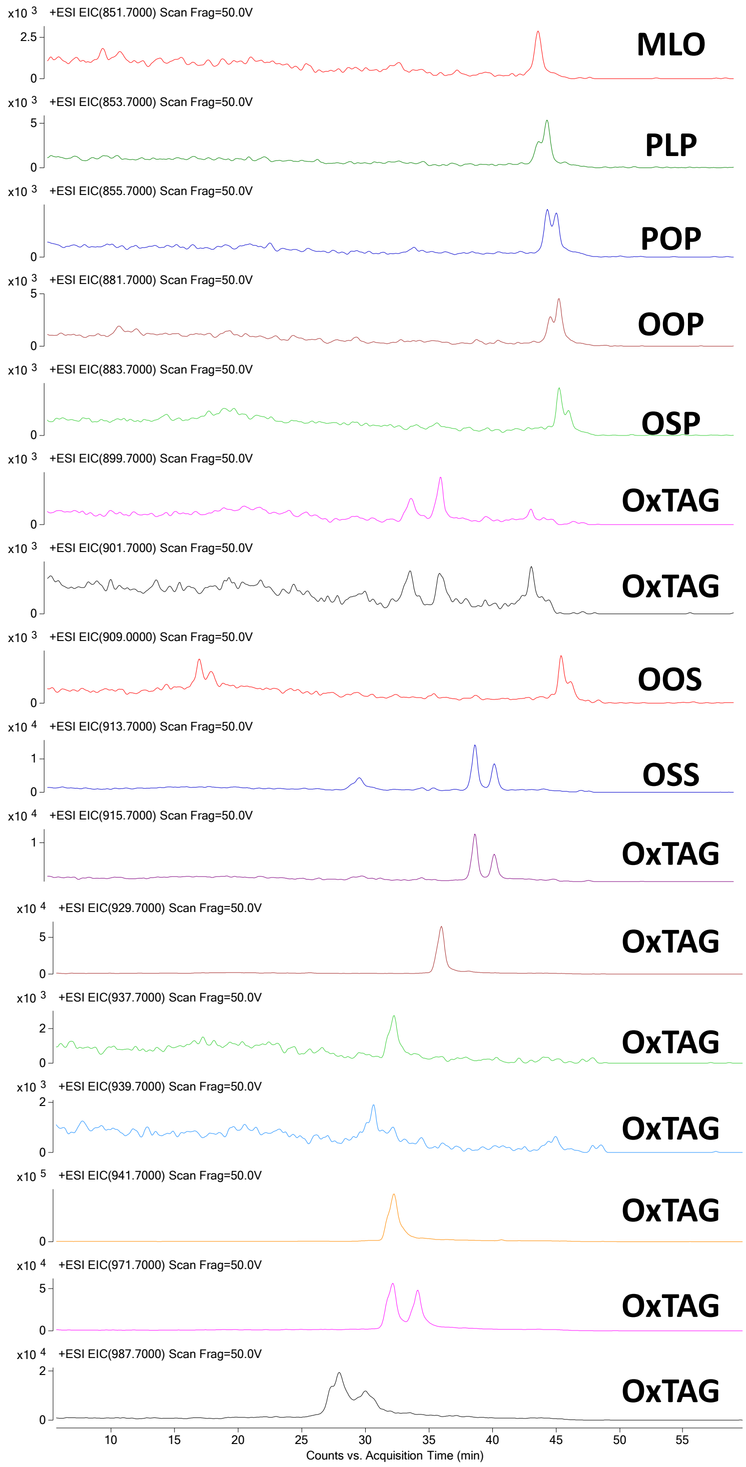


**Figure S.26** - HPLC-ESI-Q-ToF extract ion chromatograms of the of the extracts of sample 07. Abbreviation list: M: myristic acid, P: palmitic acid, L: linoleic acid; O: oleic acid, S:stearic acid; OxTAGs: m/z 897.7 ($C_{18:1,1OH}$OP, $[M+Na]^+$), m/z 899.7 ($C_{18:1,1OH}$SP, $[M+Na]^+$), m/z 901 ($C_{18,1OH}$SP, $[M+Na]^+$), m/z 915.7 ($C_{18,1OH}$ $C_{18:1,1OH}$P, $[M+Na]^+$), m/z 929.7 ($C_{18:1,2OH}$ $C_{18:1,1OH}$P, $[M+Na]^+$), m/z 941.7 ($C_{18:1,1OH}$ $C_{18:1,1OH}$S, $[M+Na]^+$), m/z 971.7 ($C_{18:1,2OH}$ $C_{18:1,1OH}$ $C_{18:1,1OH}$, $[M+Na]^+$), m/z 987.7 ($C_{18:1,2OH}$ $C_{18:1,2OH}$ $C_{18:1,1OH}$, $[M+Na]^+$)

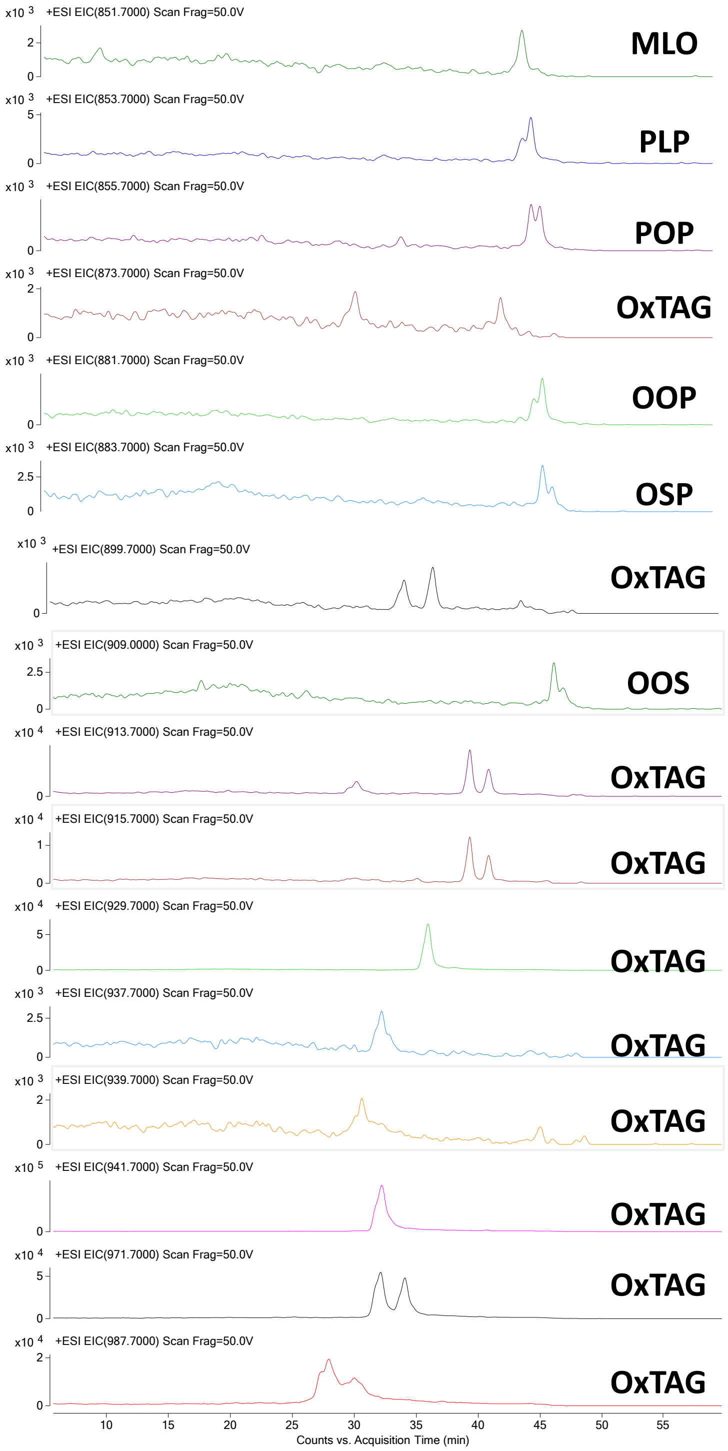


**Figure S.27** - HPLC-ESI-Q-ToF extract ion chromatograms of the of the extracts of sample 08. Abbreviation list: M: myristic acid, P: palmitic acid, L: linoleic acid; O: oleic acid, S:stearic acid; OxTAGs: m/z 897.7 ($C_{18:1,1OH}$OP, $[M+Na]^+$), m/z 899.7 ($C_{18:1,1OH}$SP, $[M+Na]^+$), m/z 901 ($C_{18,1OH}$SP, $[M+Na]^+$), m/z 913.7 ($C_{18:1,1OH}$ $C_{18:1,1OH}$P, $[M+Na]^+$), m/z 929.7 ($C_{18:1,2OH}$ $C_{18:1,1OH}$P, $[M+Na]^+$), m/z 941.7 ($C_{18:1,1OH}$ $C_{18:1,1OH}$S, $[M+Na]^+$), m/z 971.7 ($C_{18:1,2OH}$ $C_{18:1,1OH}$ $C_{18:1,1OH}$, $[M+Na]^+$), m/z 987.7 ($C_{18:1,2OH}$ $C_{18:1,2OH}$ $C_{18:1,1OH}$, $[M+Na]^+$)

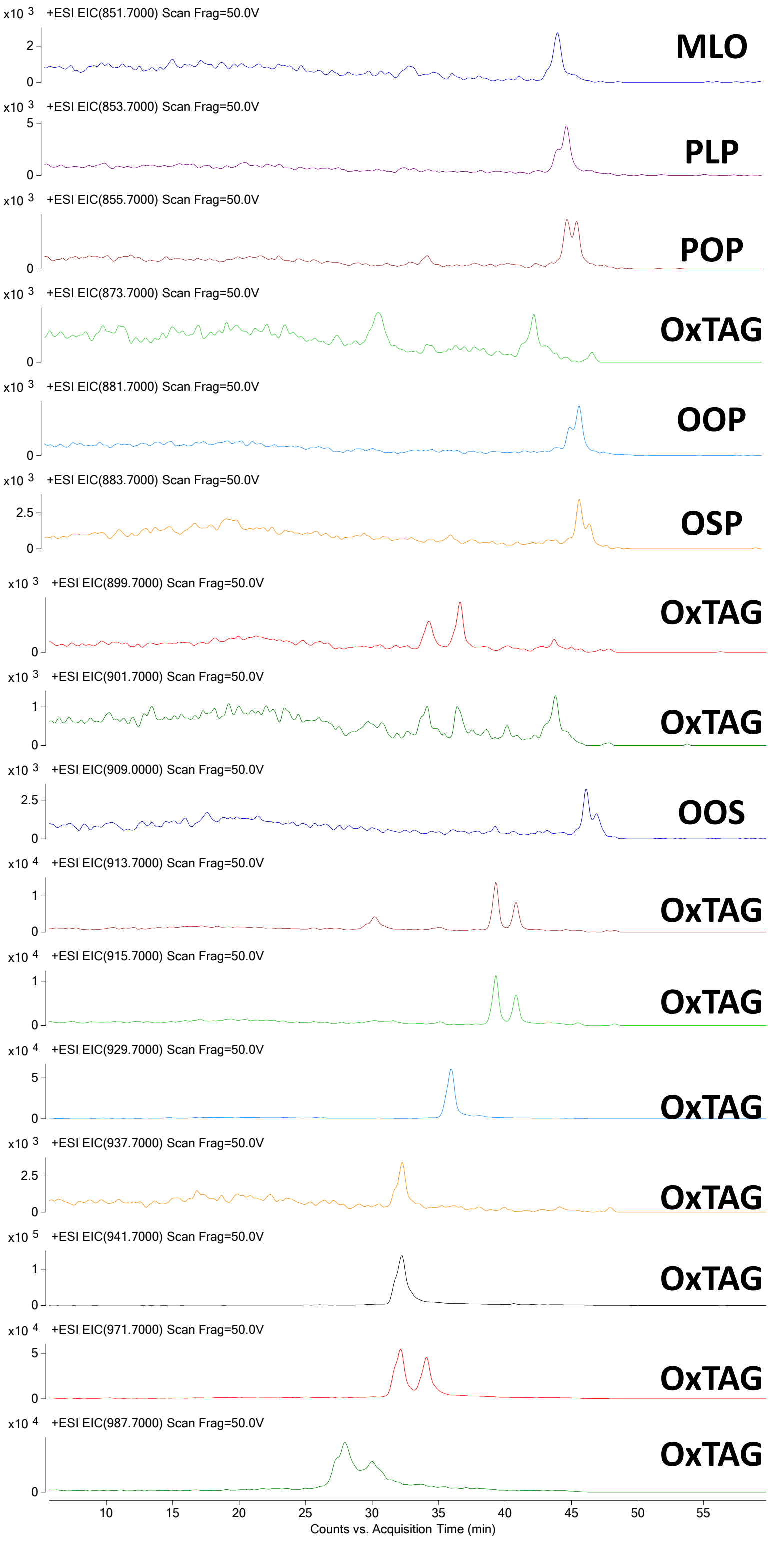


**Figure S.28** - HPLC-ESI-Q-ToF extract ion chromatograms of the of the extracts of sample 093. Abbreviation list: M: myristic acid, P: palmitic acid, L: linoleic acid; O: oleic acid, S:stearic acid; OxTAGs: m/z 897.7 ($C_{18:1,1OH}$OP, $[M+Na]^+$), m/z 899.7 ($C_{18:1,1OH}$SP, $[M+Na]^+$), m/z 901 ($C_{18,1OH}$SP, $[M+Na]^+$), m/z 913.7 ($C_{18:1,1OH}$ $C_{18:1,1OH}$P, $[M+Na]^+$), m/z 915.7 ($C_{18,1OH}$ $C_{18:1,1OH}$P, $[M+Na]^+$), m/z 929.7 ($C_{18:1,2OH}$ $C_{18:1,1OH}$P, $[M+Na]^+$), m/z 941.7 ($C_{18:1,1OH}$ $C_{18:1,1OH}$S, $[M+Na]^+$), m/z 971.7 ($C_{18:1,2OH}$ $C_{18:1,1OH}$ $C_{18:1,1OH}$, $[M+Na]^+$), m/z 987.7 ($C_{18:1,2OH}$ $C_{18:1,2OH}$ $C_{18:1,1OH}$, $[M+Na]^+$)

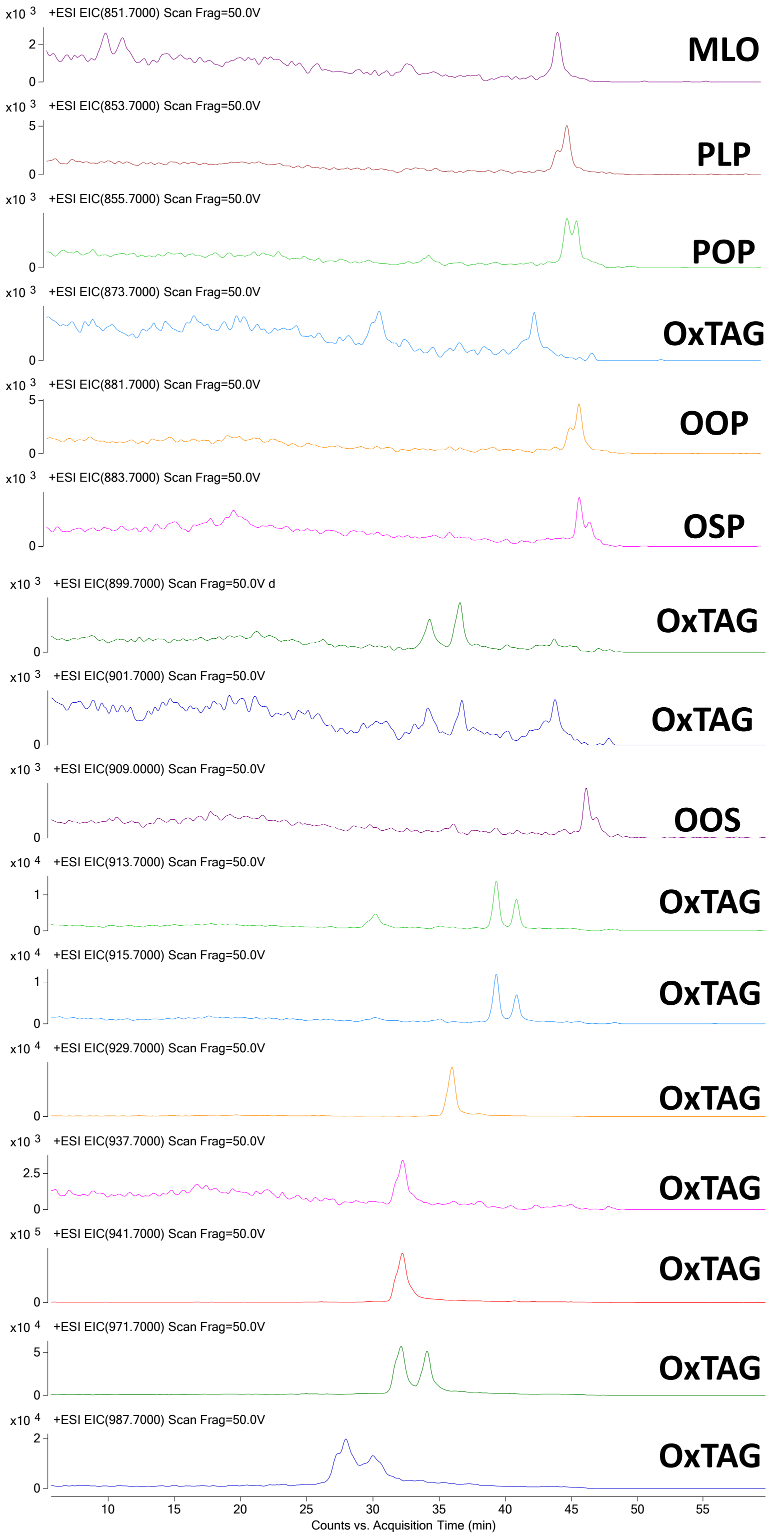


**Figure S.29** - HPLC-ESI-Q-ToF extract ion chromatograms of the of the extracts of sample 10. Abbreviation list: M: myristic acid, P: palmitic acid, L: linoleic acid; O: oleic acid, S:stearic acid; OxTAGs: m/z 897.7 ($C_{18:1,1OH}$OP, $[M+Na]^+$), m/z 899.7 ($C_{18:1,1OH}$SP, $[M+Na]^+$), m/z 901 ($C_{18,1OH}$SP, $[M+Na]^+$), m/z 913.7 ($C_{18:1,1OH}$ $C_{18:1,1OH}$P, $[M+Na]^+$), m/z 915.7 ($C_{18,1OH}$ $C_{18:1,1OH}$P, $[M+Na]^+$), m/z 929.7 ($C_{18:1,2OH}$ $C_{18:1,1OH}$P, $[M+Na]^+$), m/z 941.7 ($C_{18:1,1OH}$ $C_{18:1,1OH}$S, $[M+Na]^+$), m/z 971.7 ($C_{18:1,2OH}$ $C_{18:1,1OH}$ $C_{18:1,1OH}$, $[M+Na]^+$), m/z 987.7 ($C_{18:1,2OH}$ $C_{18:1,2OH}$ $C_{18:1,1OH}$, $[M+Na]^+$)

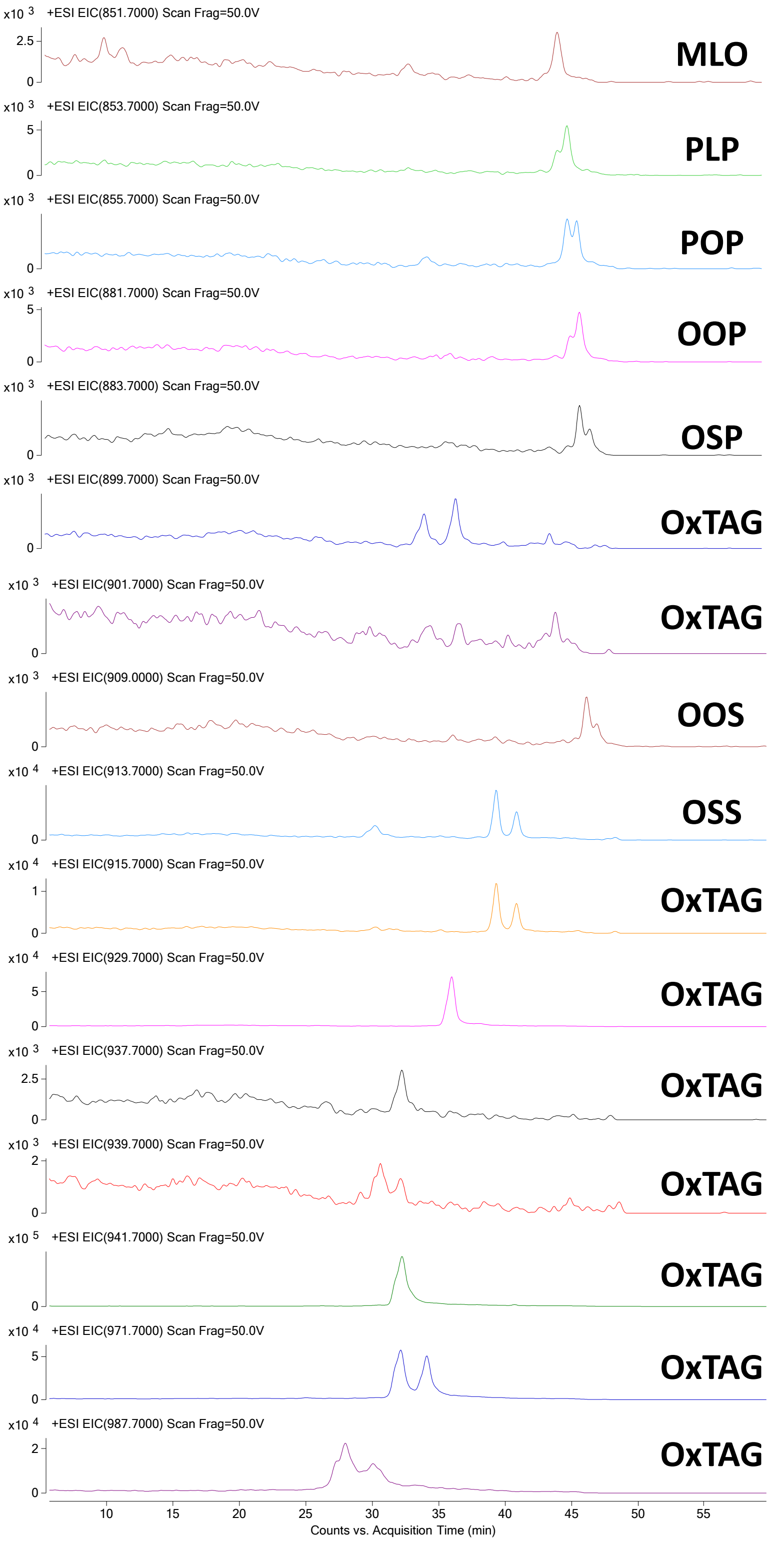


**Figure S.30** - HPLC-ESI-Q-ToF extract ion chromatograms of the of the extracts of sample 11. Abbreviation list: M: myristic acid, P: palmitic acid, L: linoleic acid; O: oleic acid, S:stearic acid; OxTAGs: m/z 897.7 ($C_{18:1,1OH}$OP, $[M+Na]^+$), m/z 899.7 ($C_{18:1,1OH}$SP, $[M+Na]^+$), m/z 901 ($C_{18,1OH}$SP, $[M+Na]^+$), m/z 913.7 ($C_{18:1,1OH}$ $C_{18:1,1OH}$P, $[M+Na]^+$), m/z 915.7 ($C_{18,1OH}$ $C_{18:1,1OH}$P, $[M+Na]^+$), m/z 929.7 ($C_{18:1,2OH}$ $C_{18:1,1OH}$P, $[M+Na]^+$), m/z 941.7 ($C_{18:1,1OH}$ $C_{18:1,1OH}$S, $[M+Na]^+$), m/z 971.7 ($C_{18:1,2OH}$ $C_{18:1,1OH}$ $C_{18:1,1OH}$, $[M+Na]^+$), m/z 987.7 ($C_{18:1,2OH}$ $C_{18:1,2OH}$ $C_{18:1,1OH}$, $[M+Na]^+$)

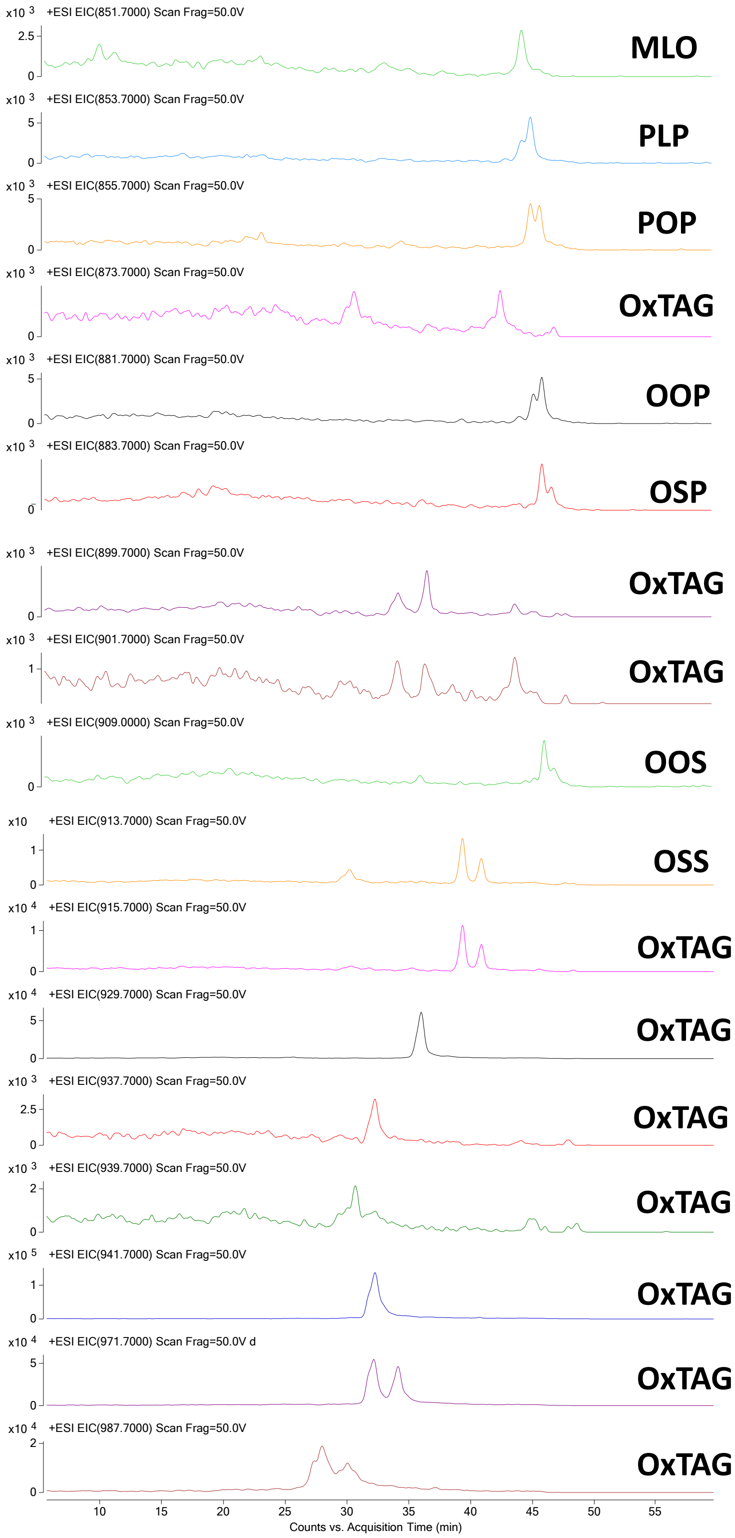


**Figure S.31** - HPLC-ESI-Q-ToF extract ion chromatograms of the of the extracts of sample 14. Abbreviation list: M: myristic acid, P: palmitic acid, L: linoleic acid; O: oleic acid, S:stearic acid; OxTAGs: m/z 897.7 ($C_{18:1,1OH}$OP, $[M+Na]^+$), m/z 899.7 ($C_{18:1,1OH}$SP, $[M+Na]^+$), m/z 901 ($C_{18,1OH}$SP, $[M+Na]^+$), m/z 913.7 ($C_{18:1,1OH}$ $C_{18:1,1OH}$P, $[M+Na]^+$), m/z 915.7 ($C_{18,1OH}$ $C_{18:1,1OH}$P, $[M+Na]^+$), m/z 929.7 ($C_{18:1,2OH}$ $C_{18:1,1OH}$P, $[M+Na]^+$), m/z 941.7 ($C_{18:1,1OH}$ $C_{18:1,1OH}$S, $[M+Na]^+$), m/z 971.7 ($C_{18:1,2OH}$ $C_{18:1,1OH}$ $C_{18:1,1OH}$, $[M+Na]^+$), m/z 987.7 ($C_{18:1,2OH}$ $C_{18:1,2OH}$ $C_{18:1,1OH}$, $[M+Na]^+$)

## S6. HPLC-ESI-Q-ToF and HPLC-DAD results for the dyes and organic pigments

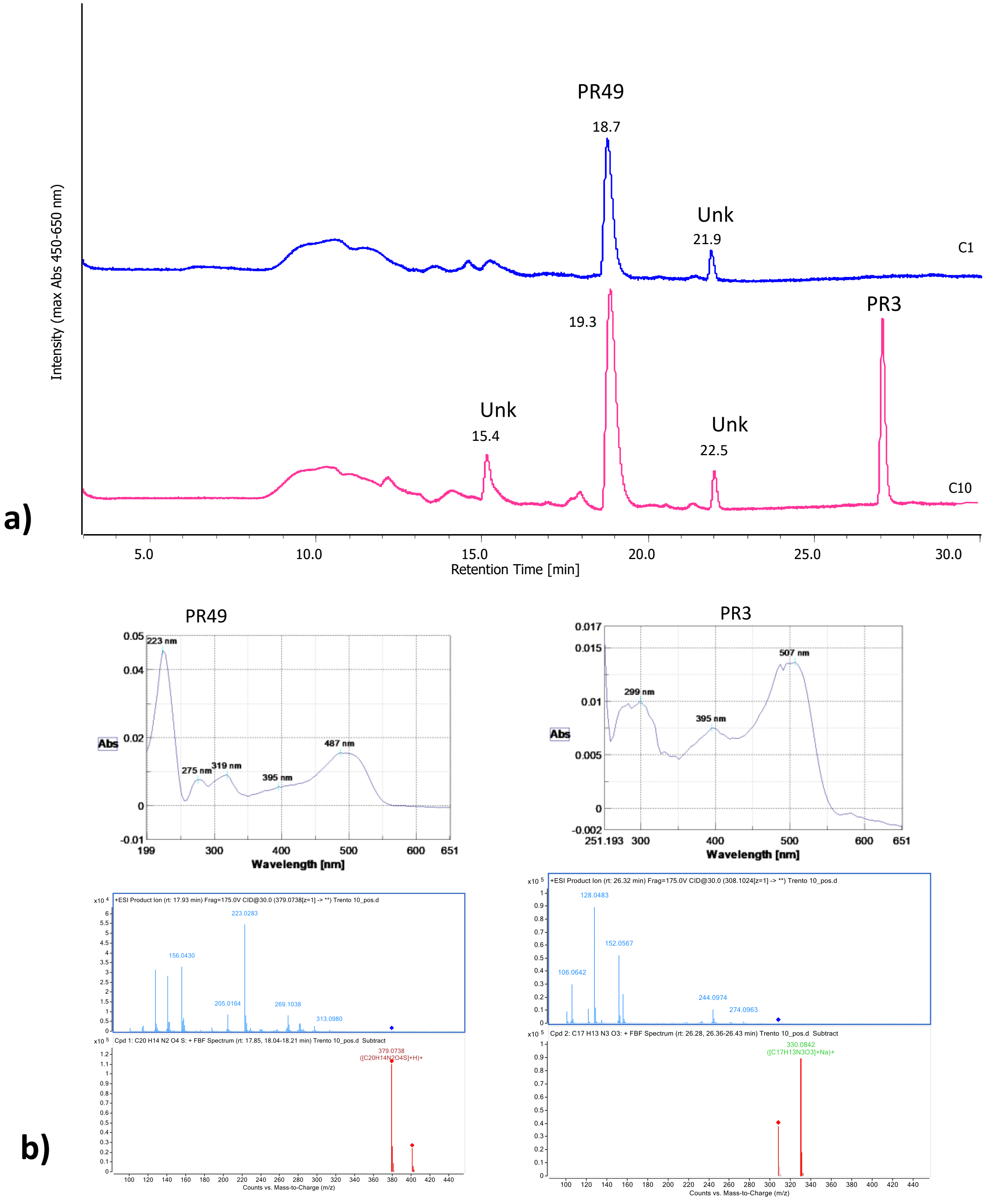


**Figure S.32** - HPLC-DAD chromatograms obtained for samples 1 and 10 (a); UV-Vis spectra and mass spectra obtained for the identified organic pigments (b)

S7. VOCs analysis sampling positions and results

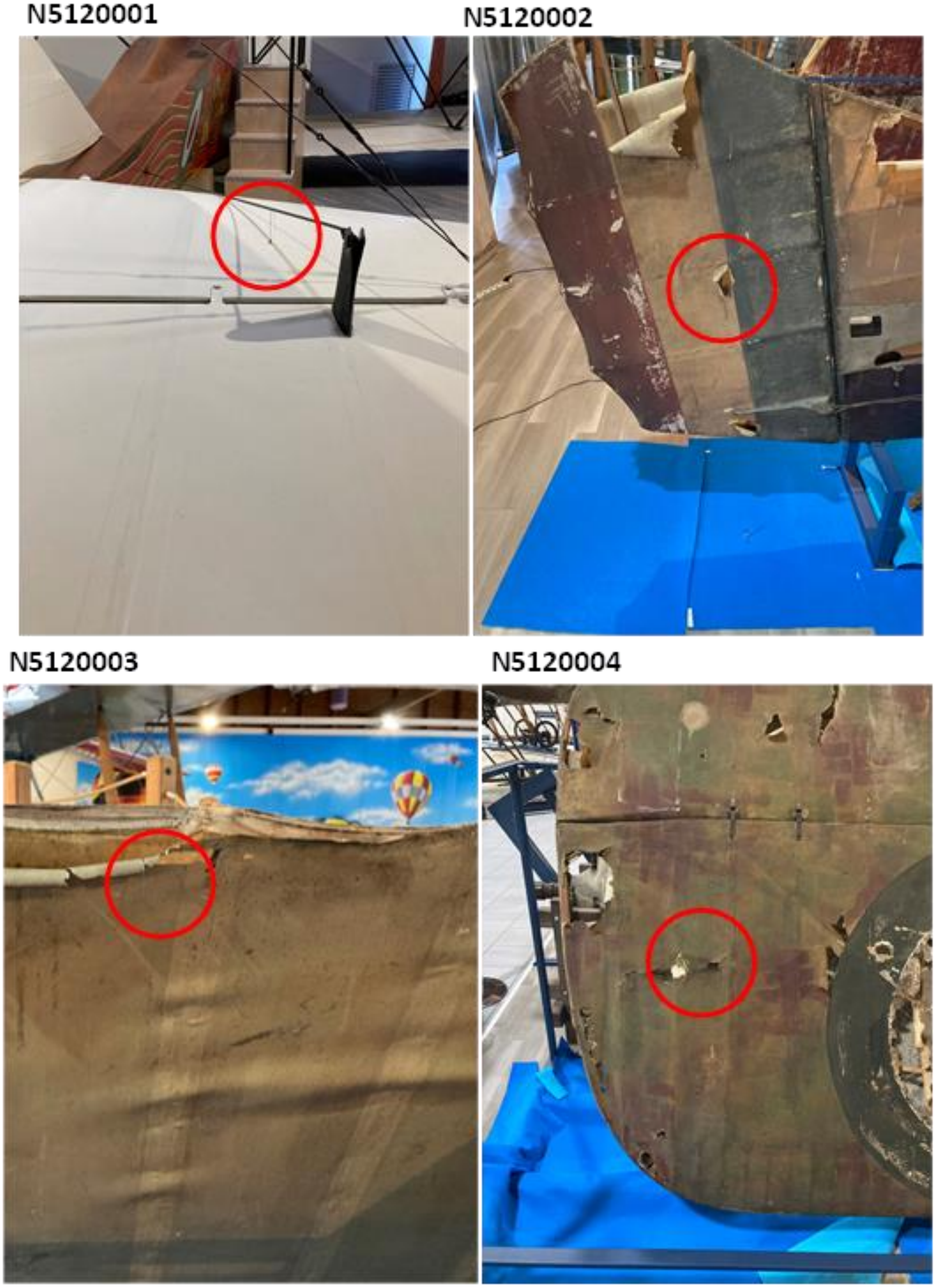


**Figure S.33** - Chemisorber sampling positions

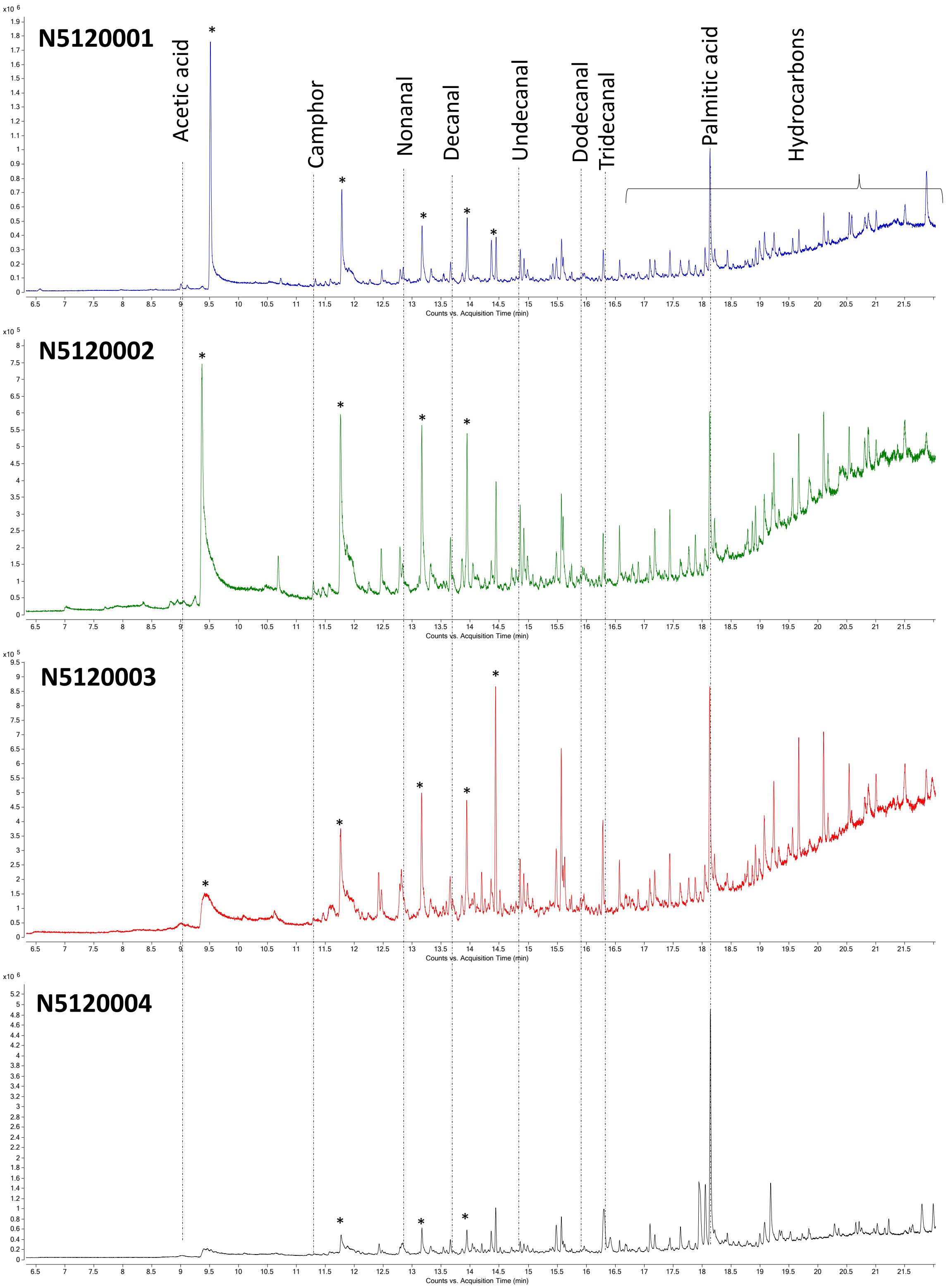


**Figure S.34** – Organic volatile compounds detected by Py-GC-MS using a passive sampling device in different sampling positions (Chemisorber)

## S8. Py-GC-MS chromatograms and sampling positions of the wood samples

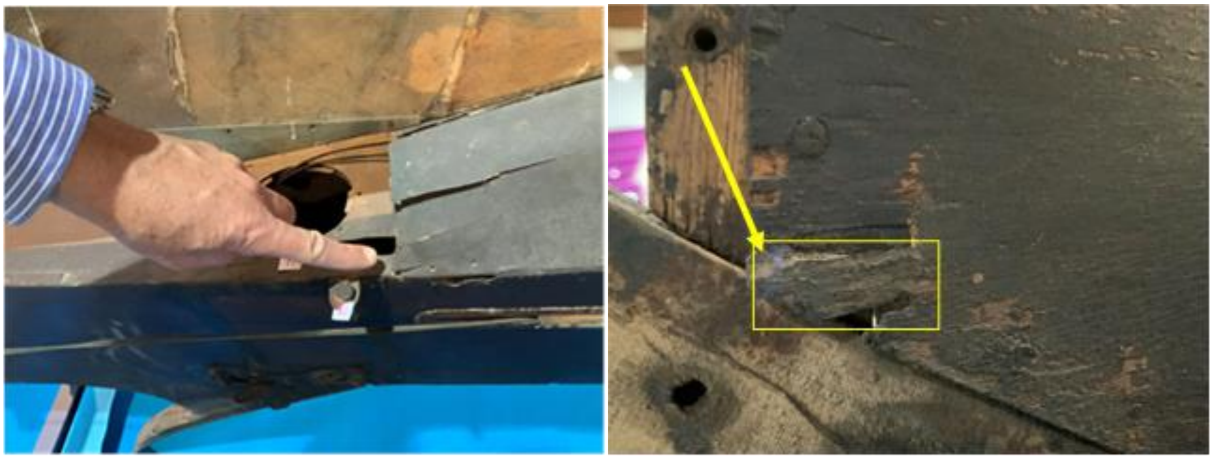

**Figure S.35** – Sampling positions of the wood samples (a: left; b: right)

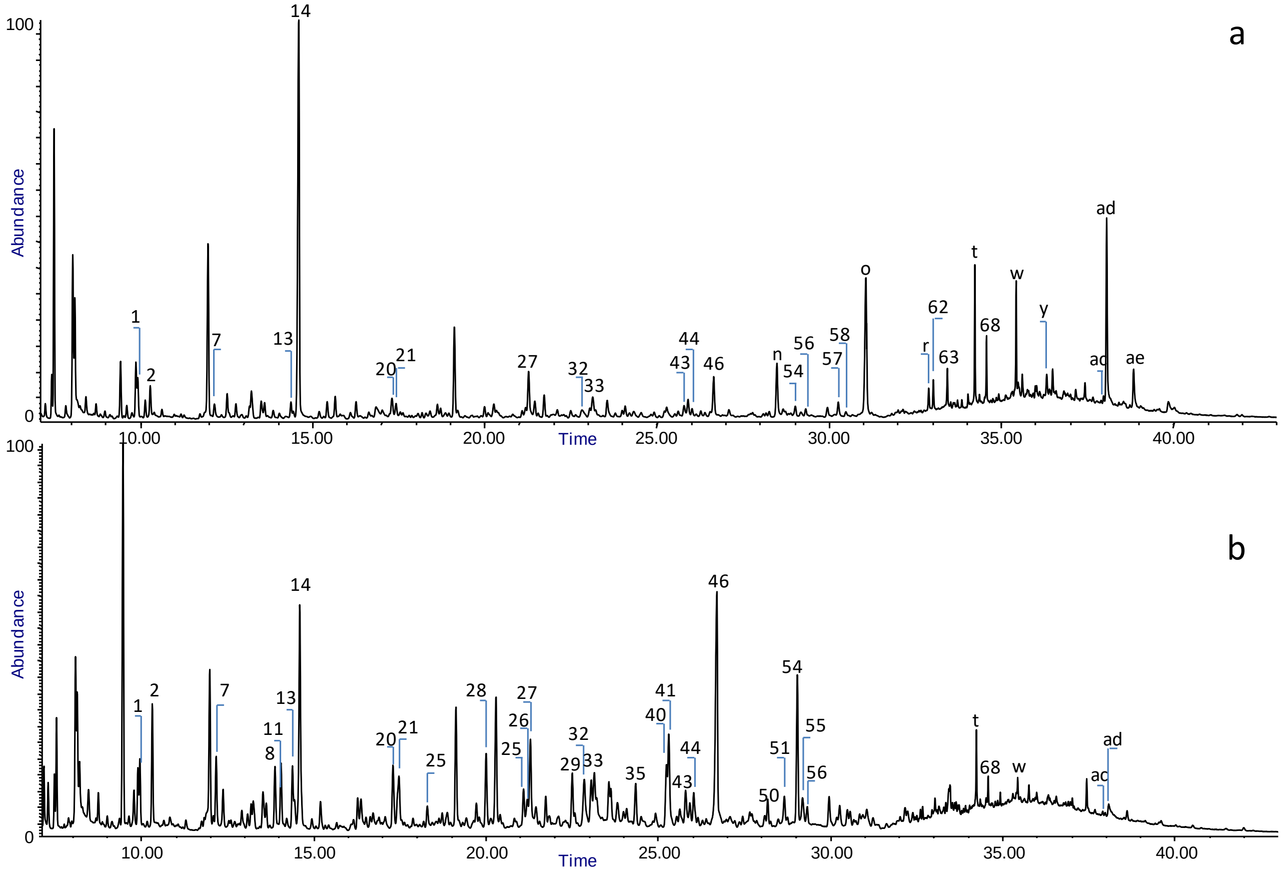


**Figure S.36** – Py-GC-MS chromatograms obtained for two wood samples; the complete list of pyrolysis products detected in the chromatogram is reported in Table S.

Table S.1 - Compounds identified in Py-GC-MS chromatograms of the wood samples (Figure S.36)

| n° | Compounds | Retention time | Sample A | Sample B |
|---|---|---|---|---|
| 1 | 2-hydroxypropanoic acid (2TMS) | 9.94 | x | x |
| 2 | 2-hydroxyacetic acid (2TMS) | 10.30 | x | x |
| 3 | 1-hydroxy-1-cyclopenten-3-one (TMS) | 11.28 | x | x |
| 4 | o-cresol (TMS) | 11.81 | x | x |
| 5 | 2-furancarboxylic acid (TMS) | 11.90 | x | |
| 6 | m-cresol (TMS) | 12.07 | x | x |
| 7 | 2-hydroxy-1-cyclopenten-3-one (TMS) | 12.15 | x | x |
| 8 | 3-hydroxy-(2H)-pyran-2-one (TMS) | 12.70 | x | x |
| 9 | E-4,5-dihydroxy-2-cyclopenten-1-one (TMS) | 13.55 | x | |
| 10 | 3-hydroxy-(4H)-pyran-4-one (TMS) | 13.86 | x | |
| 11 | 5-hydroxy-2H-pyran-4(3H)-one (TMS) | 14.04 | x | x |
| 12 | 2-methyl-3-hydroxymethyl-2-cyclopentenone (TMS) | 16.71 | x | x |
| 13 | 1-hydroxy-2-methyl-1- cyclopenten-3-one (TMS) | 14.43 | x | x |
| 14 | guaiacol (TMS) | 14.62 | x | x |
| 15 | benzoic acid TMS | 15.20 | x | x |
| 16 | 3-hydroxy-6-methyl-(2H)-pyran-2-one (TMS) | 16.07 | x | x |
| 17 | 2-hydroxymethyl-3-methy-2-cyclopentenone (TMS) | 16.08 | x | |
| 18 | vinyl phenol (TMS) | 16.15 | x | |
| 19 | 2-methyl-3-hydroxy-(4H)-pyran-4-one (TMS) | 16.51 | x | x |
| 20 | 5-hydroxymethyl-2-furaldehyde (TMS) | 17.29 | x | x |
| 21 | 4-methylguaiacol (TMS) | 17.42 | x | |
| 22 | 1,2-dihydroxybenzene (2TMS) | 17.47 | x | |
| 23 | p-hydroxy benzaldehyde TMS | 17.51 | x | x |
| 24 | 2-hydroxymethyl-2,3-dihydropyran-4-one (TMS) | 17.87 | x | |
| 25 | 4-hydroxy-5,6-dihydro-(2H)-pyran-4-one (TMS) | 18.29 | x | x |
| 26 | 4-methylcatechol (2TMS) | 19.71 | x | |
| 27 | 4-ethylguaiacol (TMS) | 19.80 | x | x |
| 28 | syringol (TMS) | 19.99 | x | x |
| 29 | resorcinol, 2TMS derivative | 20.01 | x | x |
| 30 | arabinofuranose (4TMS) | 20.28 | x | x |
| 31 | 4-vinylguaiacol (TMS) | 21.09 | x | x |
| 32 | 3-hydroxy-2-hydroxymethyl-2-cyclopentenone (2TMS) | 21.20 | x | x |
| 33 | E-4,5-dihydroxy-2-cyclopenten-1-one (2TMS) | 21.29 | x | x |
| 34 | 4-ethylcatechol (2TMS) | 21.85 | x | x |
| 35 | 4-methylsyringol (TMS) | 22.50 | x | x |
| 36 | 3-methoxy-1,2-benzenediol (2TMS) | 22.50 | x | |
| 37 | 3,5-dihydroxy-2-methyl-(4H)-pyran-4-one (2TMS) | 22.58 | x | |
| 38 | 1,6-anydro-beta-D-glucopyranose (TMS at position 4) | 22.85 | x | x |
| 39 | 1,6-anydro-beta-D-glucopyranose (TMS at position 2) | 23.14 | x | |
| 40 | vanillin (TMS) | 23.99 | x | |

| | | | | |
|---|---|---|---|---|
| 41 | 1,2,3-trihydroxybenzene (3TMS) | 24.34 | x | x |
| 42 | 5-methyl-3-methoxy-1,2-benzenediol (2TMS) | 24.35 | x | |
| 43 | 4-ethylsyringol (TMS) | 24.50 | x | |
| 44 | 4-hydroxy benzoic acid (2TMS) | 24.85 | x | x |
| 45 | Z-isoeugenol (TMS) | 24.92 | x | x |
| 46 | 1,4-anydro-D-galactopyranose (2TMS) | 25.24 | x | x |
| 47 | 1,6-anydro-D-galactopyranose (2TMS) | 25.31 | x | x |
| 48 | 2-hydroxymethyl-5-hydroxy-2,3-dihydro-(4H)-pyran-4-one (2TMS) | 25.62 | x | x |
| 49 | 4-vinylsyringol (TMS) | 25.79 | x | x |
| 50 | 1,2,4-trihydroxybenzene (3TMS) | 26.03 | x | x |
| 51 | acetovanillone (TMS) | 26.39 | x | |
| 52 | 1,6-anydro-beta-D-glucopyranose (2TMS at position 2 and 4) | 26.70 | x | x |
| 53 | 4-hydroxy-3.5-dimethoxy cinnamic acid methyl ester (TMS) | 27.45 | x | x |
| 54 | propenyl-syringol (TMS) | 27.85 | | x |
| 55 | 3,5-dihydroxy-2-methyl-(4H)-pyran-4-one (2TMS) | 27.91 | | x |
| 56 | 1,4-anydro-D-galactopyranose (3TMS) | 28.18 | x | x |
| 57 | 1,6-anydro-beta-D-glucofuranose (3TMS) | 28.66 | x | |
| 58 | syringaldehyde (TMS) | 28.67 | x | x |
| 59 | 2,3,5-trihydroxy-4H-pyran-4-one (3TMS) | 28.83 | | x |
| 60 | 1,6-anydro-beta-D-glucopyranose (3TMS) | 29.04 | x | x |
| 61 | 1,4-anhydro-D-glucopyranose (3TMS) | 29.19 | x | x |
| 62 | E-propenylsyringol (TMS) | 29.33 | x | x |
| 63 | vanillic acid (2TMS) | 30.27 | x | x |
| 64 | acetosyringone (TMS) | 30.49 | x | x |
| 65 | coumaryl alcohol (2 TMS) | 30.68 | x | x |
| 66 | Z-coniferyl alcohol (2 TMS) | 32.10 | | x |
| 67 | coniferylaldehyde (TMS) | 32.22 | | x |
| 68 | syringic acid (2TMS) | 33.04 | x | x |
| 69 | E-coniferyl alcohol( 2 TMS) | 33.44 | x | x |
| 70 | 3,4-dihydroxy-5-methoxy benzoic acid (3TMS) | 33.52 | | x |
| 71 | syringylpropanol (2TMS) | 33.62 | x | x |
| 72 | sinapylaldehyde (TMS) | 34.04 | | x |
| 73 | synapyl alcohol (TMS) | 34.52 | x | x |
| 74 | E-synapyl alcohol (2TMS) | 34.58 | x | x |

## S9. FEG-SEM data

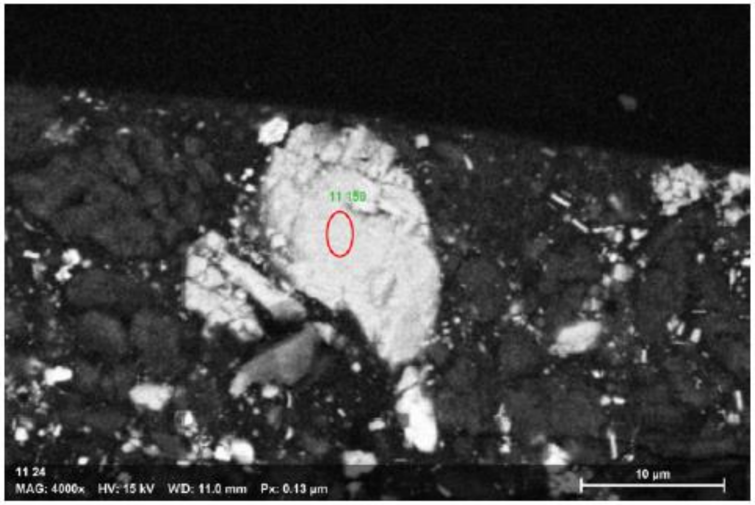


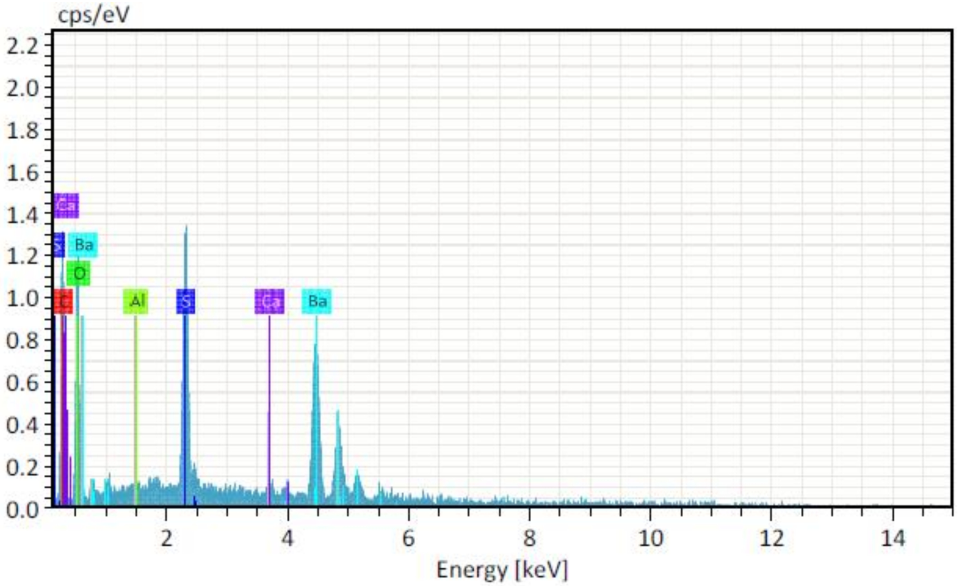


11 150

| Element | At. No. | Netto | Mass [%] | Mass Norm. [%] | Atom [%] | abs. error [%] (1 sigma) |
|---|---|---|---|---|---|---|
| Carbon | 6 | 1839 | 23.21 | 28.49 | 55.85 | 4.51 |
| Oxygen | 8 | 1801 | 15.55 | 19.09 | 28.09 | 3.05 |
| Aluminium | 13 | 116 | 0.40 | 0.49 | 0.42 | 0.07 |
| Sulfur | 16 | 3341 | 9.55 | 11.72 | 8.60 | 0.42 |
| Calcium | 20 | 82 | 0.28 | 0.35 | 0.20 | 0.06 |
| Barium | 56 | 4910 | 32.49 | 39.88 | 6.84 | 1.10 |
| | | **Sum** | **81.48** | **100.00** | **100.00** | |

**Figure S.37** – FEG-SEM elemental analysis results obtained for sample 11

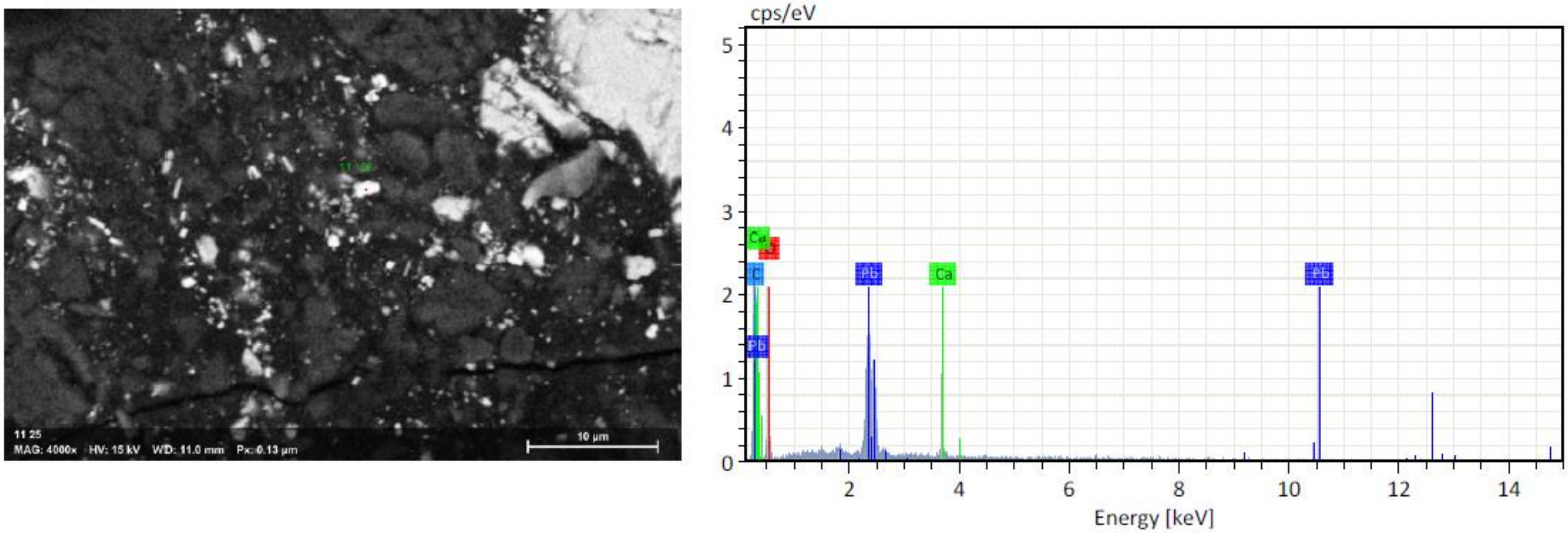


11 152

| Element | At. No. | Netto | Mass [%] | Mass Norm. [%] | Atom [%] | abs. error [%] (1 sigma) |
|---|---|---|---|---|---|---|
| Carbon | 6 | 3310 | 32.31 | 37.74 | 72.64 | 5.50 |
| Oxygen | 8 | 838 | 12.42 | 14.50 | 20.96 | 2.98 |
| Calcium | 20 | 380 | 1.97 | 2.31 | 1.33 | 0.15 |
| Lead | 82 | 6669 | 38.92 | 45.45 | 5.07 | 1.53 |
| | | **Sum** | **85.62** | **100.00** | **100.00** | |

Figure S.38 – FEG-SEM elemental analysis results obtained for sample 11

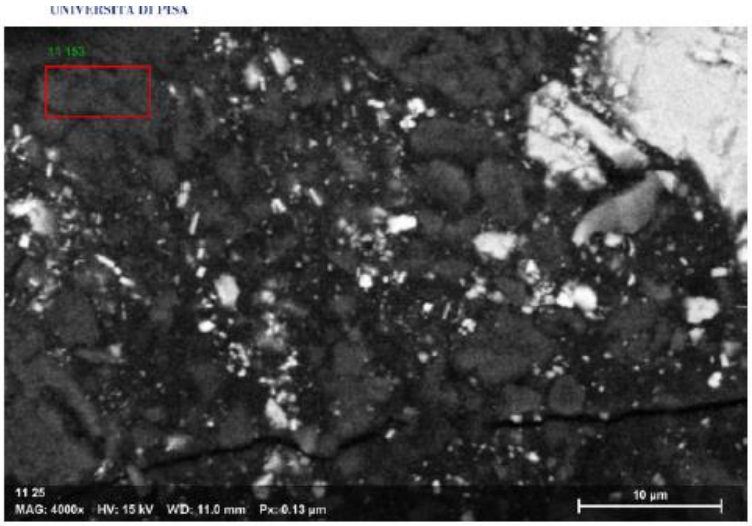


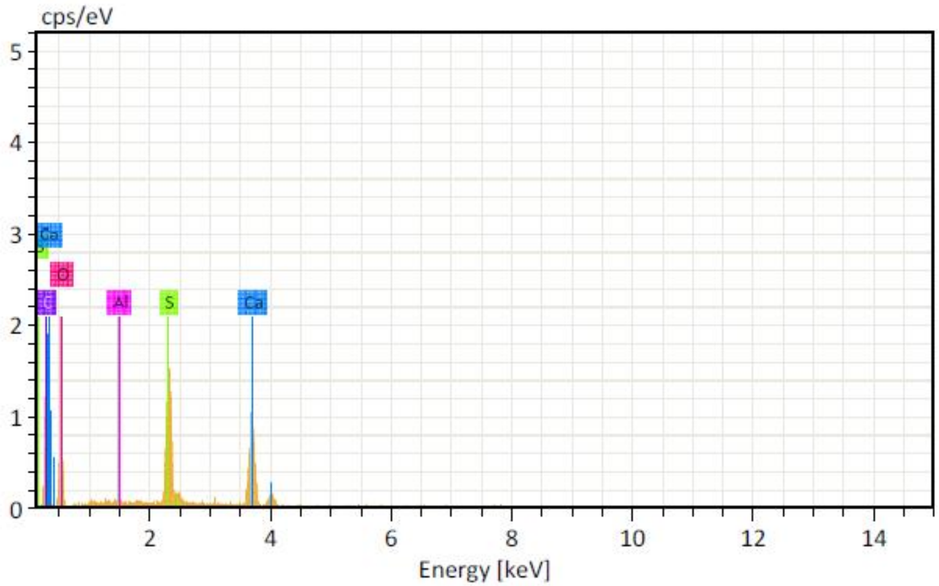


11 153

| Element | At. No. | Netto | Mass [%] | Mass Norm. [%] | Atom [%] | abs. error [%] (1 sigma) |
|---|---|---|---|---|---|---|
| Carbon | 6 | 1809 | 38.09 | 41.25 | 56.25 | 7.42 |
| Oxygen | 8 | 1246 | 27.67 | 29.96 | 30.67 | 5.93 |
| Aluminium | 13 | 86 | 0.26 | 0.29 | 0.17 | 0.06 |
| Sulfur | 16 | 3610 | 11.34 | 12.28 | 6.27 | 0.49 |
| Calcium | 20 | 2861 | 14.99 | 16.23 | 6.63 | 0.58 |
| | | **Sum** | **92.35** | **100.00** | **100.00** | |

**Figure S.39** – FEG-SEM elemental analysis results obtained for sample 11

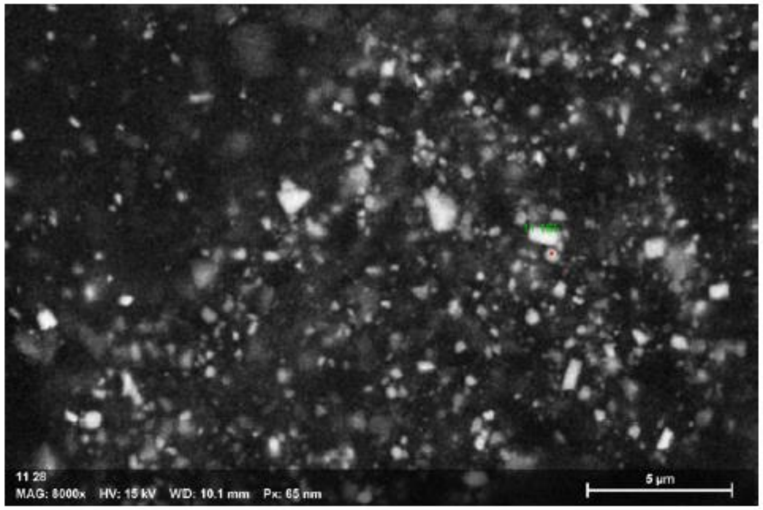


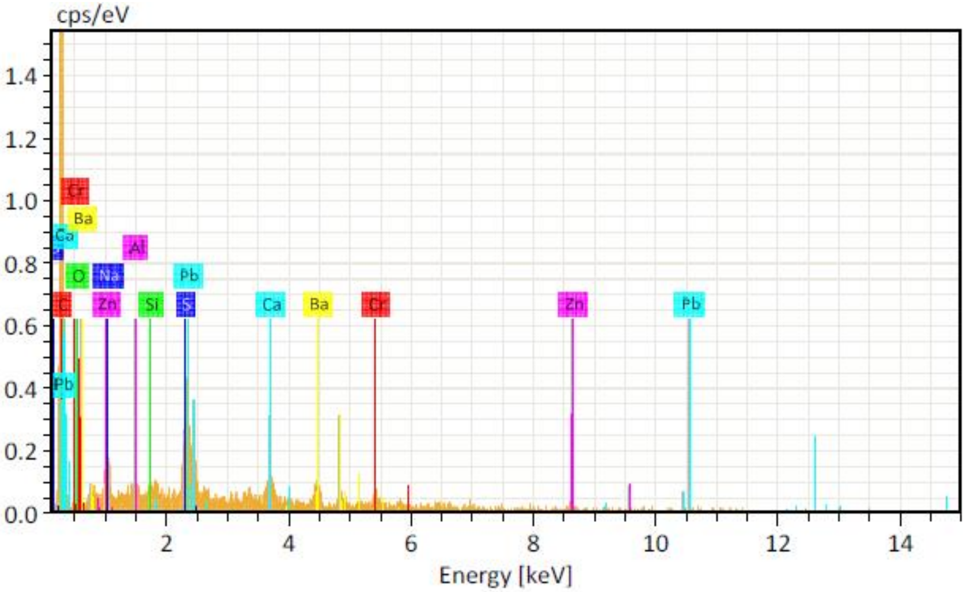


11 165

| Element | At. No. | Netto | Mass [%] | Mass Norm. [%] | Atom [%] | abs. error [%] (1 sigma) |
|---|---|---|---|---|---|---|
| Carbon | 6 | 3610 | 47.06 | 60.42 | 80.29 | 7.86 |
| Oxygen | 8 | 539 | 10.67 | 13.69 | 13.66 | 2.93 |
| Sodium | 11 | 116 | 0.60 | 0.77 | 0.53 | 0.11 |
| Aluminium | 13 | 83 | 0.32 | 0.41 | 0.24 | 0.07 |
| Silicon | 14 | 77 | 0.28 | 0.36 | 0.20 | 0.06 |
| Sulfur | 16 | 617 | 2.14 | 2.74 | 1.37 | 0.15 |
| Calcium | 20 | 326 | 1.78 | 2.28 | 0.91 | 0.15 |
| Chromium | 24 | 81 | 0.63 | 0.81 | 0.25 | 0.10 |
| Zinc | 30 | 79 | 4.23 | 5.43 | 1.33 | 0.56 |
| Barium | 56 | 432 | 4.17 | 5.36 | 0.62 | 0.28 |
| Lead | 82 | 838 | 6.01 | 7.71 | 0.59 | 0.36 |
| | | **Sum** | **77.89** | **100.00** | **100.00** | |

Figure S.40 – FEG-SEM elemental analysis results obtained for sample 11

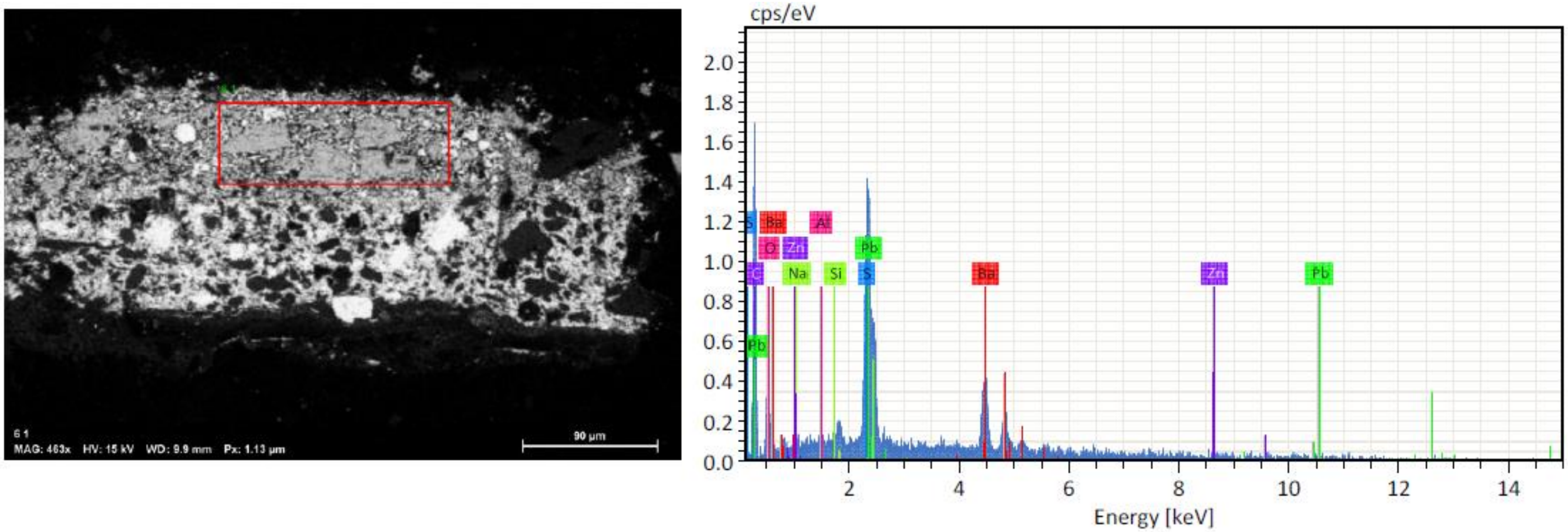


6 1

| Element | At. No. | Netto | Mass [%] | Mass Norm. [%] | Atom [%] | abs. error [%] (1 sigma) |
|---|---|---|---|---|---|---|
| Carbon | 6 | 1615 | 27.13 | 29.03 | 63.83 | 5.44 |
| Oxygen | 8 | 783 | 12.84 | 13.74 | 22.68 | 3.14 |
| Sodium | 11 | 40 | 0.33 | 0.35 | 0.40 | 0.09 |
| Aluminium | 13 | 58 | 0.27 | 0.29 | 0.29 | 0.07 |
| Silicon | 14 | 41 | 0.16 | 0.17 | 0.16 | 0.05 |
| Sulfur | 16 | 1159 | 4.57 | 4.89 | 4.02 | 0.26 |
| Zinc | 30 | 55 | 2.29 | 2.45 | 0.99 | 0.36 |
| Barium | 56 | 1848 | 19.68 | 21.06 | 4.05 | 0.80 |
| Lead | 82 | 3212 | 26.19 | 28.02 | 3.57 | 1.12 |
| | | **Sum** | **93.44** | **100.00** | **100.00** | |

**Figure S.41** – FEG-SEM elemental analysis results obtained for sample 6

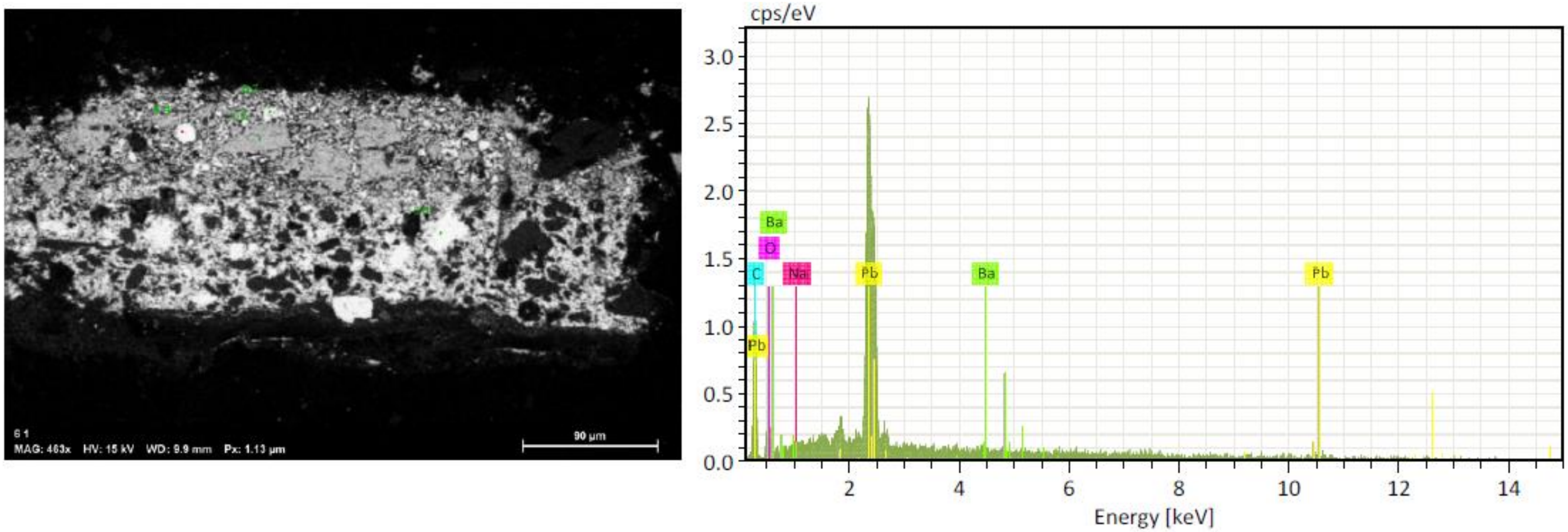


6 4

| Element | At. No. | Netto | Mass [%] | Mass Norm. [%] | Atom [%] | abs. error [%] (1 sigma) |
|---|---|---|---|---|---|---|
| Carbon | 6 | 1780 | 17.11 | 20.31 | 63.44 | 3.36 |
| Oxygen | 8 | 672 | 8.24 | 9.79 | 22.95 | 2.12 |
| Sodium | 11 | 84 | 0.43 | 0.51 | 0.83 | 0.09 |
| Barium | 56 | 235 | 2.03 | 2.41 | 0.66 | 0.18 |
| Lead | 82 | 11823 | 56.41 | 66.97 | 12.12 | 2.12 |
| | | **Sum** | **84.23** | **100.00** | **100.00** | |

Figure S.42 – FEG-SEM elemental analysis results obtained for sample 6

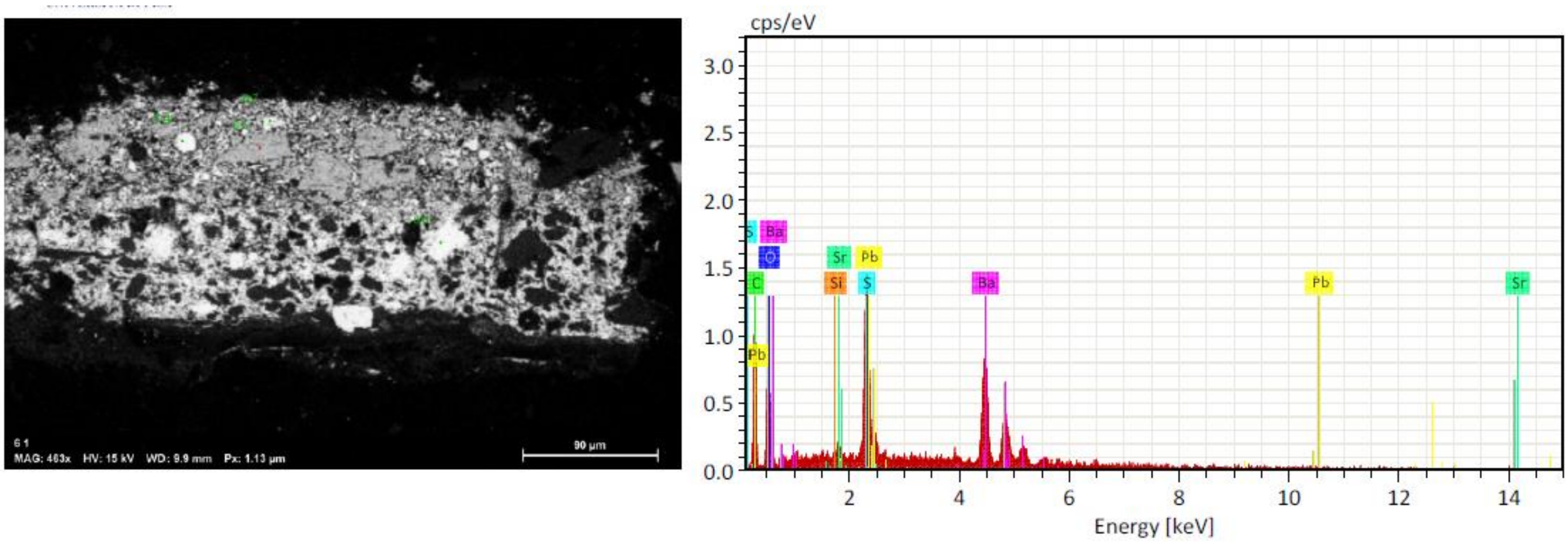


65

| Element | At. No. | Netto | Mass [%] | Mass Norm. [%] | Atom [%] | abs. error [%] (1 sigma) |
|---|---|---|---|---|---|---|
| Carbon | 6 | 1173 | 19.76 | 22.56 | 50.94 | 4.31 |
| Oxygen | 8 | 1373 | 15.44 | 17.63 | 29.89 | 3.24 |
| Silicon | 14 | 77 | 0.31 | 0.36 | 0.35 | 0.07 |
| Sulfur | 16 | 2655 | 10.12 | 11.55 | 9.77 | 0.46 |
| Strontium | 38 | 175 | 1.23 | 1.40 | 0.43 | 0.15 |
| Barium | 56 | 4043 | 33.38 | 38.10 | 7.53 | 1.16 |
| Lead | 82 | 955 | 7.37 | 8.41 | 1.10 | 0.42 |
| | | **Sum** | **87.61** | **100.00** | **100.00** | |

Figure S.43 – FEG-SEM elemental analysis results obtained for sample 6

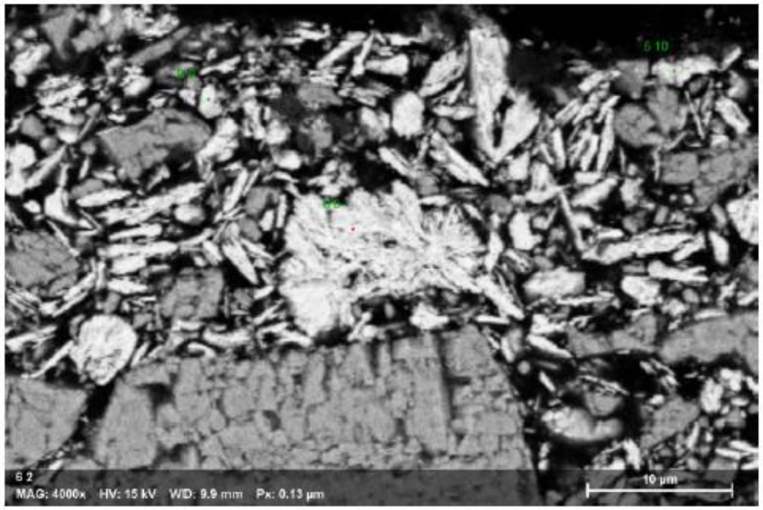


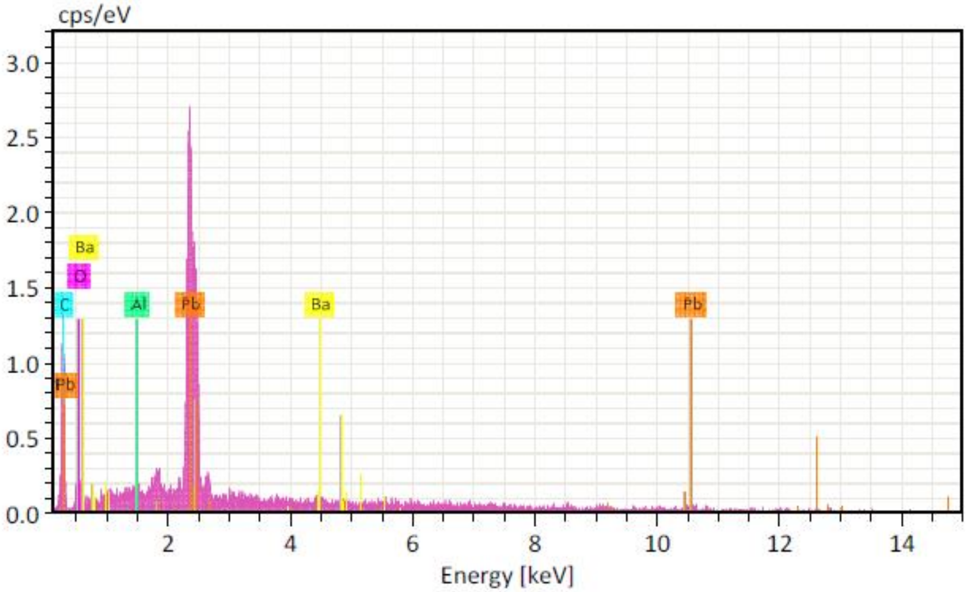


6 8

| Element | At. No. | Netto | Mass [%] | Mass Norm. [%] | Atom [%] | abs. error [%] (1 sigma) |
|---|---|---|---|---|---|---|
| Carbon | 6 | 1707 | 18.32 | 20.40 | 63.04 | 3.63 |
| Oxygen | 8 | 680 | 9.27 | 10.32 | 23.95 | 2.37 |
| Aluminium | 13 | 74 | 0.23 | 0.26 | 0.35 | 0.06 |
| Barium | 56 | 303 | 2.83 | 3.15 | 0.85 | 0.22 |
| Lead | 82 | 11042 | 59.16 | 65.87 | 11.80 | 2.23 |
| | | **Sum** | **89.81** | **100.00** | **100.00** | |

**Figure S.44** – FEG-SEM elemental analysis results obtained for sample 6

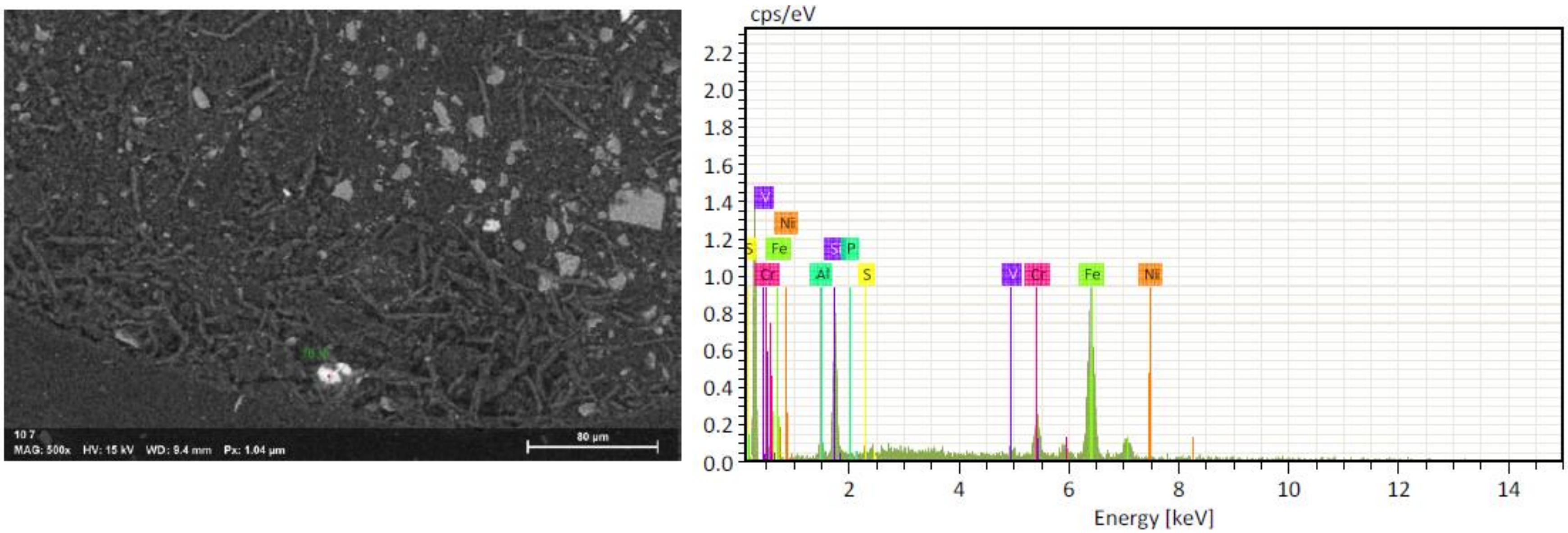


10 15

| Element | At. No. | Netto | Mass [%] | Mass Norm. [%] | Atom [%] | abs. error [%] (1 sigma) |
|---|---|---|---|---|---|---|
| Aluminium | 13 | 162 | 2.50 | 3.07 | 5.21 | 0.29 |
| Silicon | 14 | 1605 | 14.52 | 17.85 | 29.14 | 0.80 |
| Phosphorus | 15 | 18 | 0.13 | 0.16 | 0.23 | 0.06 |
| Sulfur | 16 | 9 | 0.05 | 0.06 | 0.08 | 0.02 |
| Vanadium | 23 | 82 | 0.76 | 0.93 | 0.84 | 0.12 |
| Chromium | 24 | 725 | 7.56 | 9.29 | 8.19 | 0.41 |
| Iron | 26 | 3076 | 54.89 | 67.46 | 55.38 | 2.01 |
| Nickel | 28 | 34 | 0.97 | 1.19 | 0.93 | 0.20 |
| | | **Sum** | **81.36** | **100.00** | **100.00** | |

**Figure S.45** – FEG-SEM elemental analysis results obtained for sample 10

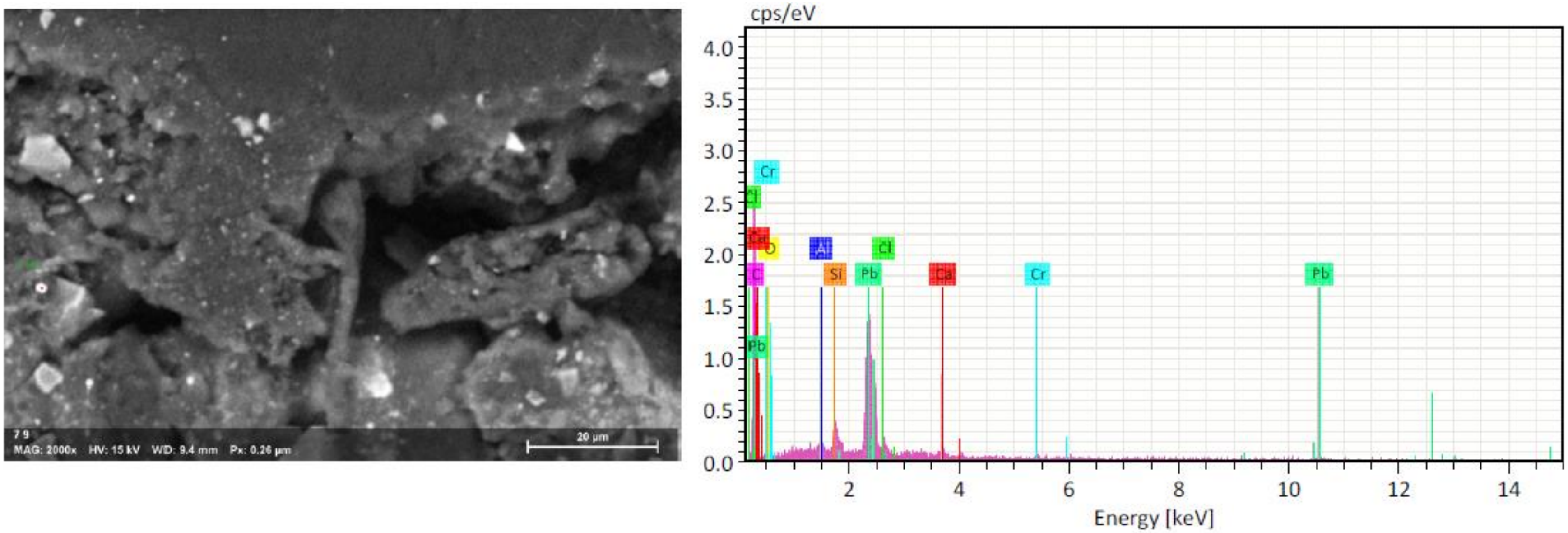


7 19

| Element | At. No. | Netto | Mass [%] | Mass Norm. [%] | Atom [%] | abs. error [%] (1 sigma) |
|---|---|---|---|---|---|---|
| Carbon | 6 | 2549 | 32.72 | 39.45 | 72.46 | 5.89 |
| Oxygen | 8 | 675 | 11.90 | 14.35 | 19.78 | 3.05 |
| Aluminium | 13 | 158 | 0.56 | 0.68 | 0.55 | 0.09 |
| Silicon | 14 | 445 | 1.45 | 1.74 | 1.37 | 0.13 |
| Chlorine | 17 | 136 | 0.65 | 0.78 | 0.49 | 0.09 |
| Calcium | 20 | 154 | 1.09 | 1.32 | 0.72 | 0.13 |
| Chromium | 24 | 36 | 0.48 | 0.58 | 0.25 | 0.11 |
| Lead | 82 | 4887 | 34.09 | 41.11 | 4.38 | 1.38 |
| | | **Sum** | **82.94** | **100.00** | **100.00** | |

**Figure S.46** – FEG-SEM elemental analysis results obtained for sample 7

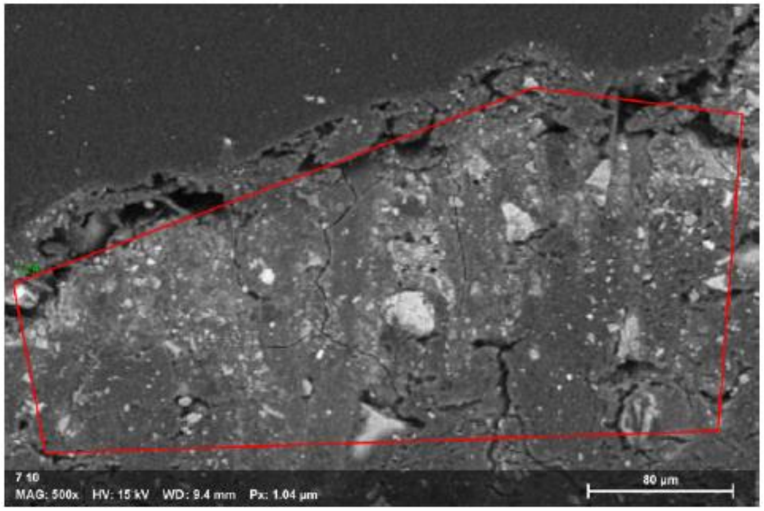


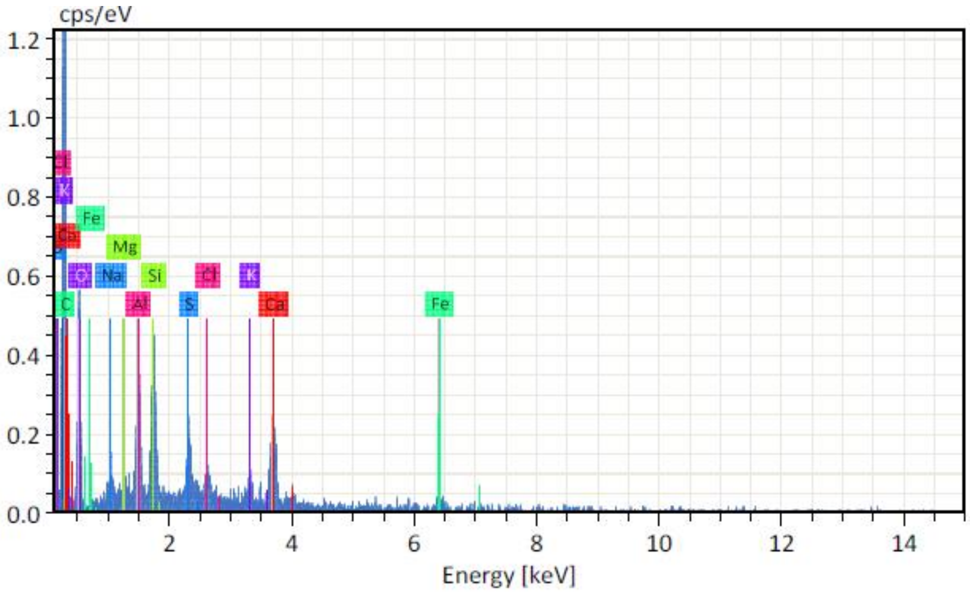


7 24

| Element | At. No. | Netto | Mass [%] | Mass Norm. [%] | Atom [%] | abs. error [%] (1 sigma) |
|---|---|---|---|---|---|---|
| Carbon | 6 | 3149 | 54.79 | 62.62 | 73.89 | 9.41 |
| Oxygen | 8 | 744 | 18.86 | 21.56 | 19.09 | 4.68 |
| Sodium | 11 | 125 | 0.81 | 0.93 | 0.57 | 0.13 |
| Magnesium | 12 | 96 | 0.43 | 0.49 | 0.28 | 0.08 |
| Aluminium | 13 | 655 | 2.34 | 2.68 | 1.41 | 0.19 |
| Silicon | 14 | 904 | 2.86 | 3.27 | 1.65 | 0.20 |
| Sulfur | 16 | 403 | 1.66 | 1.90 | 0.84 | 0.14 |
| Chlorine | 17 | 163 | 0.62 | 0.71 | 0.28 | 0.08 |
| Potassium | 19 | 132 | 0.67 | 0.77 | 0.28 | 0.09 |
| Calcium | 20 | 590 | 3.61 | 4.12 | 1.46 | 0.22 |
| Iron | 26 | 46 | 0.84 | 0.96 | 0.24 | 0.16 |
| | | **Sum** | **87.50** | **100.00** | **100.00** | |

**Figure S.47** – FEG-SEM elemental analysis results obtained for sample 7

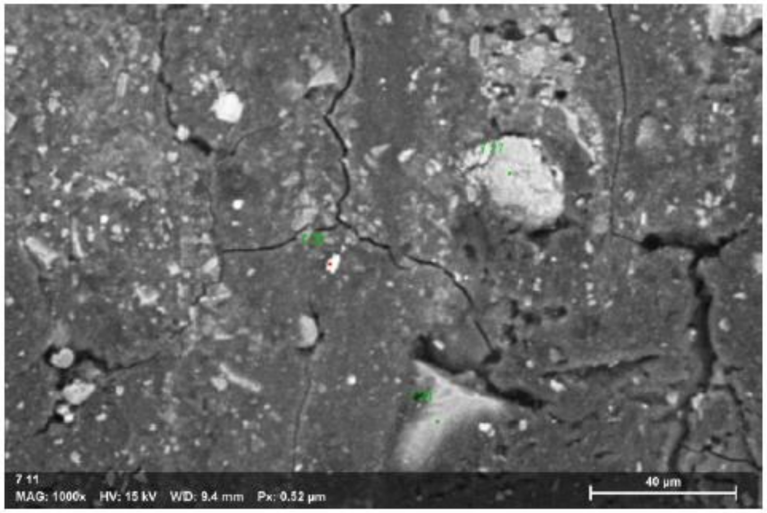


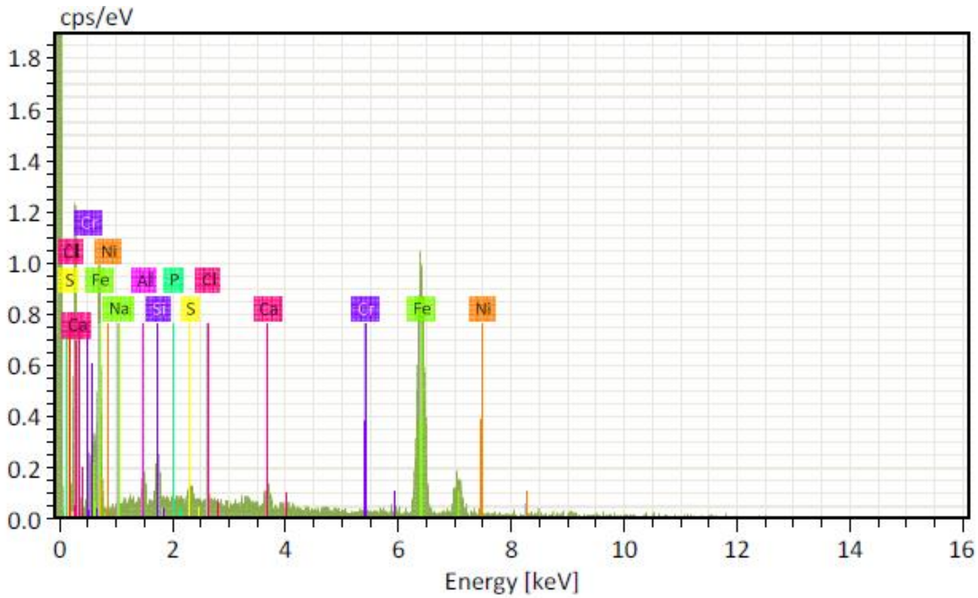


7 26

| Element | At. No. | Netto | Mass [%] | Mass Norm. [%] | Atom [%] | abs. error [%] (1 sigma) |
|---|---|---|---|---|---|---|
| Sodium | 11 | 35 | 0.39 | 0.48 | 1.09 | 0.11 |
| Aluminium | 13 | 205 | 1.21 | 1.47 | 2.88 | 0.15 |
| Silicon | 14 | 305 | 1.62 | 1.97 | 3.69 | 0.16 |
| Phosphorus | 15 | 27 | 0.15 | 0.18 | 0.30 | 0.06 |
| Sulfur | 16 | 161 | 0.90 | 1.09 | 1.80 | 0.11 |
| Chlorine | 17 | 65 | 0.43 | 0.52 | 0.78 | 0.08 |
| Calcium | 20 | 200 | 1.26 | 1.53 | 2.01 | 0.13 |
| Chromium | 24 | 20 | 0.25 | 0.31 | 0.31 | 0.08 |
| Iron | 26 | 3428 | 76.16 | 92.44 | 87.14 | 2.73 |
| Nickel | 28 | 0 | 0.00 | 0.00 | 0.00 | 0.00 |

**Figure S.48** – FEG-SEM elemental analysis results obtained for sample 7

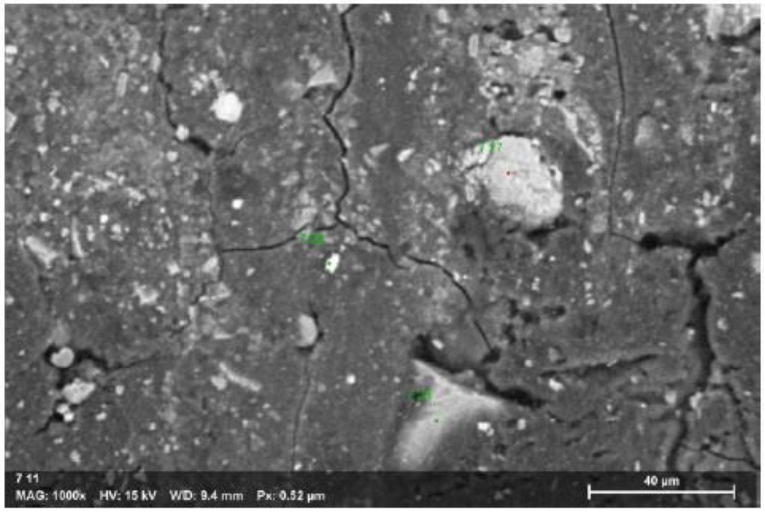


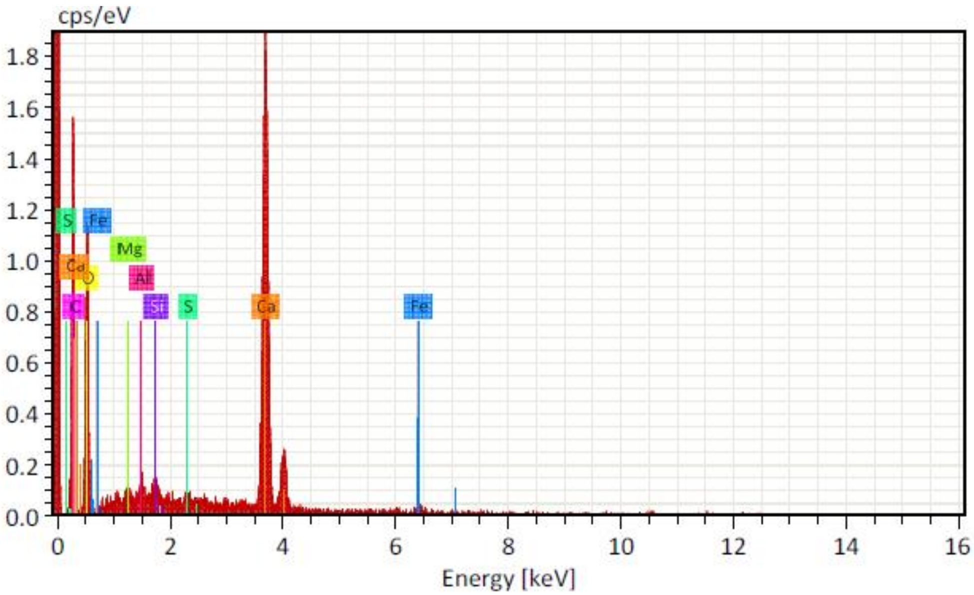


7 27

| Element | At. No. | Netto | Mass [%] | Mass Norm. [%] | Atom [%] | abs. error [%] (1 sigma) |
|---|---|---|---|---|---|---|
| Carbon | 6 | 1807 | 23.36 | 25.36 | 38.52 | 4.56 |
| Oxygen | 8 | 1488 | 36.72 | 39.86 | 45.45 | 7.51 |
| Magnesium | 12 | 69 | 0.26 | 0.28 | 0.21 | 0.06 |
| Aluminium | 13 | 110 | 0.35 | 0.38 | 0.26 | 0.07 |
| Silicon | 14 | 133 | 0.40 | 0.43 | 0.28 | 0.07 |
| Sulfur | 16 | 70 | 0.22 | 0.24 | 0.13 | 0.05 |
| Calcium | 20 | 5484 | 30.22 | 32.80 | 14.93 | 1.05 |
| Iron | 26 | 35 | 0.60 | 0.65 | 0.21 | 0.13 |
| | | **Sum** | **92.13** | **100.00** | **100.00** | |

**Figure S.49** – FEG-SEM elemental analysis results obtained for sample 7

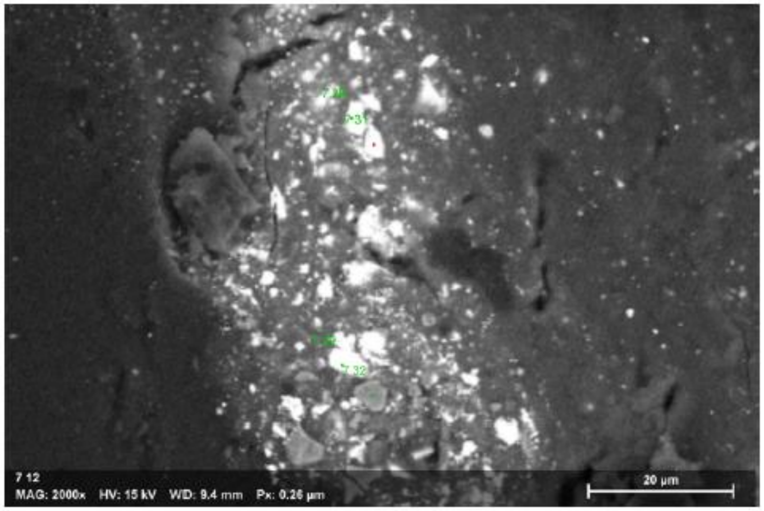


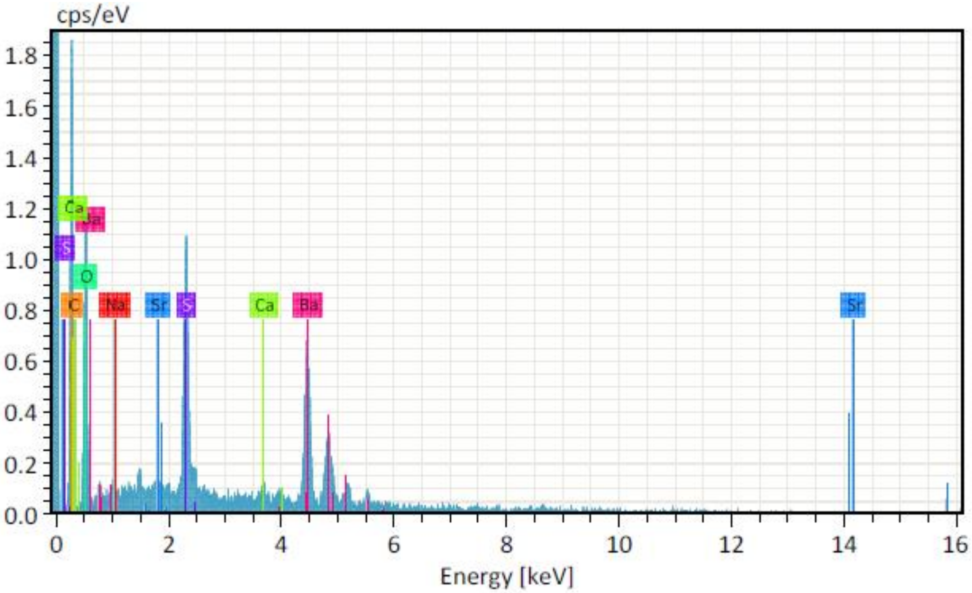


7 31

| Element | At. No. | Netto | Mass [%] | Mass Norm. [%] | Atom [%] | abs. error [%] (1 sigma) |
|---|---|---|---|---|---|---|
| Carbon | 6 | 2645 | 33.02 | 36.70 | 62.29 | 5.89 |
| Oxygen | 8 | 1793 | 18.86 | 20.96 | 26.72 | 3.69 |
| Sodium | 11 | 110 | 0.59 | 0.65 | 0.58 | 0.11 |
| Sulfur | 16 | 2677 | 7.09 | 7.88 | 5.01 | 0.33 |
| Calcium | 20 | 207 | 0.84 | 0.93 | 0.47 | 0.09 |
| Strontium | 38 | 125 | 0.49 | 0.55 | 0.13 | 0.08 |
| Barium | 56 | 3822 | 29.09 | 32.33 | 4.80 | 1.02 |
| | | **Sum** | **89.97** | **100.00** | **100.00** | |

Figure S.50 – FEG-SEM elemental analysis results obtained for sample 7

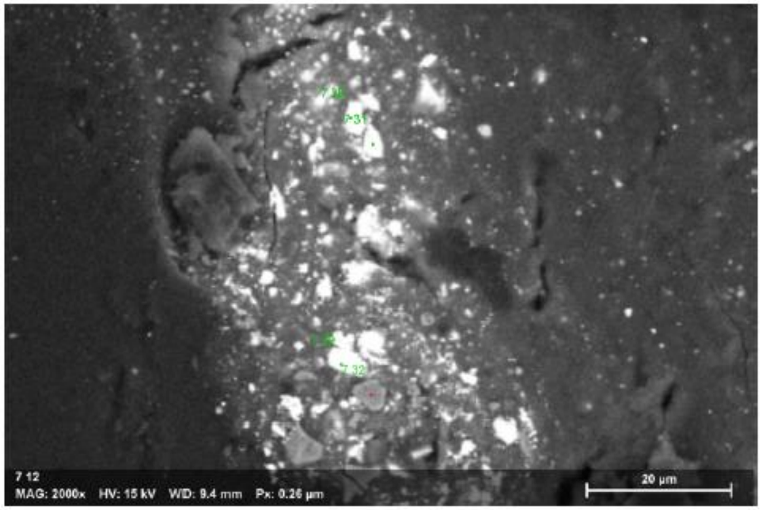


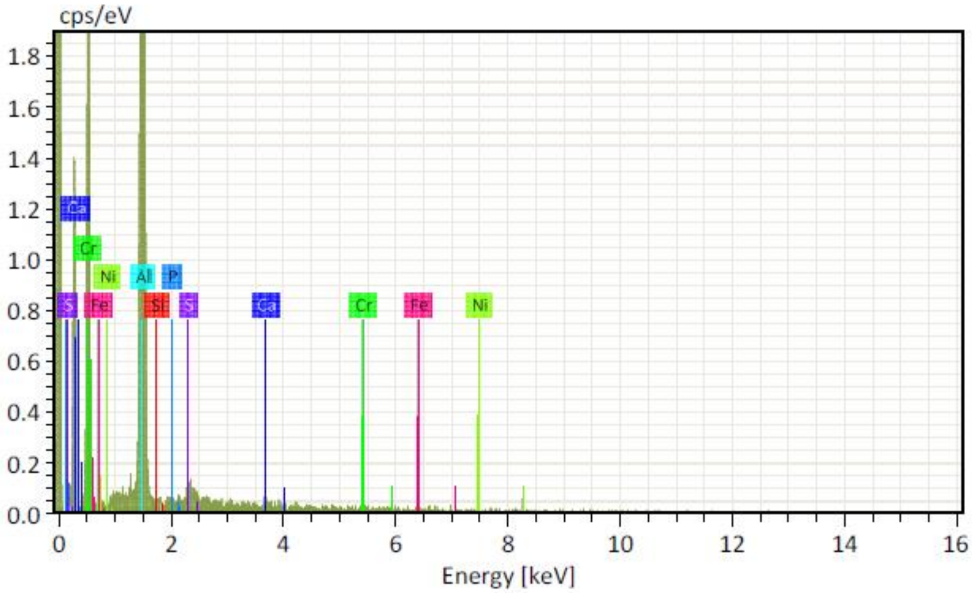


7 32

| Element | At. No. | Netto | Mass [%] | Mass Norm. [%] | Atom [%] | abs. error [%] (1 sigma) |
|---|---|---|---|---|---|---|
| Aluminium | 13 | 12745 | 29.96 | 96.41 | 97.34 | 1.47 |
| Silicon | 14 | 0 | 0.00 | 0.00 | 0.00 | 0.00 |
| Phosphorus | 15 | 10 | 0.04 | 0.12 | 0.11 | 0.01 |
| Sulfur | 16 | 140 | 0.48 | 1.56 | 1.32 | 0.07 |
| Calcium | 20 | 74 | 0.46 | 1.47 | 1.00 | 0.08 |
| Chromium | 24 | 13 | 0.14 | 0.44 | 0.23 | 0.06 |
| Iron | 26 | 0 | 0.00 | 0.00 | 0.00 | 0.00 |
| Nickel | 28 | 0 | 0.00 | 0.00 | 0.00 | 0.00 |
| | | **Sum** | **31.07** | **100.00** | **100.00** | |

**Figure S.51** – FEG-SEM elemental analysis results obtained for sample 7

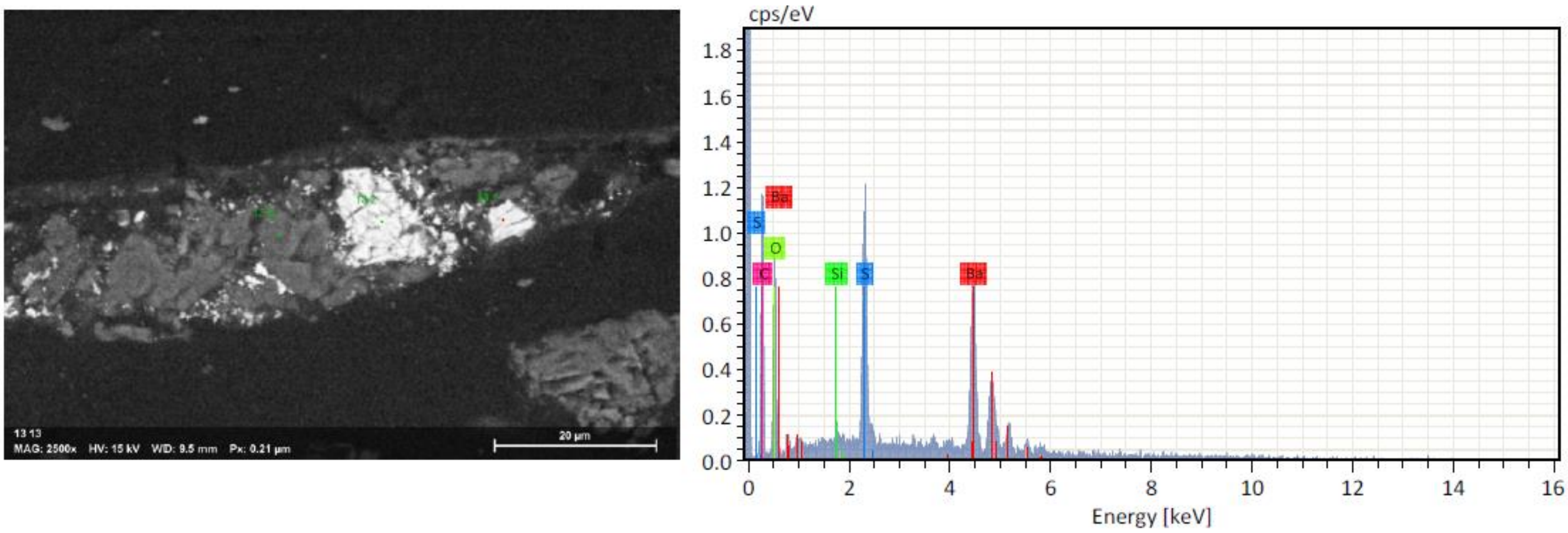


13 1

| Element | At. No. | Netto | Mass [%] | Mass Norm. [%] | Atom [%] | abs. error [%] (1 sigma) |
|---|---|---|---|---|---|---|
| Carbon | 6 | 1727 | 26.06 | 29.14 | 59.20 | 5.14 |
| Oxygen | 8 | 1379 | 14.36 | 16.05 | 24.49 | 3.01 |
| Silicon | 14 | 81 | 0.27 | 0.30 | 0.26 | 0.06 |
| Sulfur | 16 | 3038 | 9.76 | 10.91 | 8.30 | 0.44 |
| Barium | 56 | 4802 | 39.00 | 43.60 | 7.75 | 1.32 |
| | | **Sum** | **89.45** | **100.00** | **100.00** | |

Figure S.52 – FEG-SEM elemental analysis results obtained for sample 13

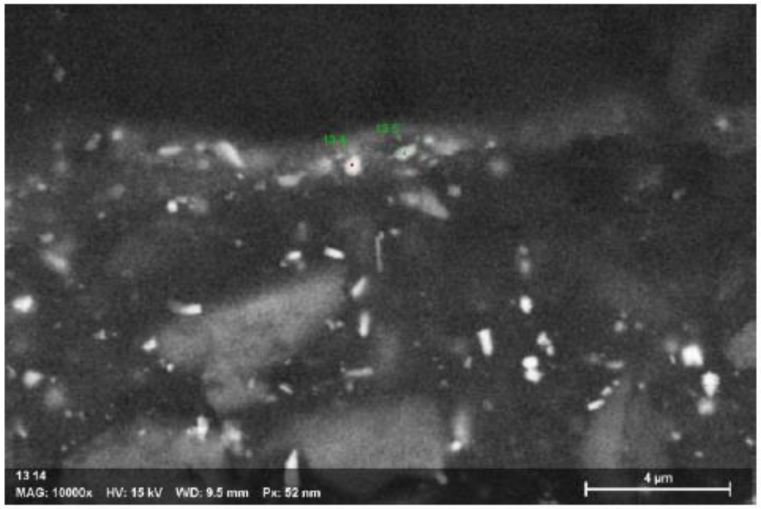


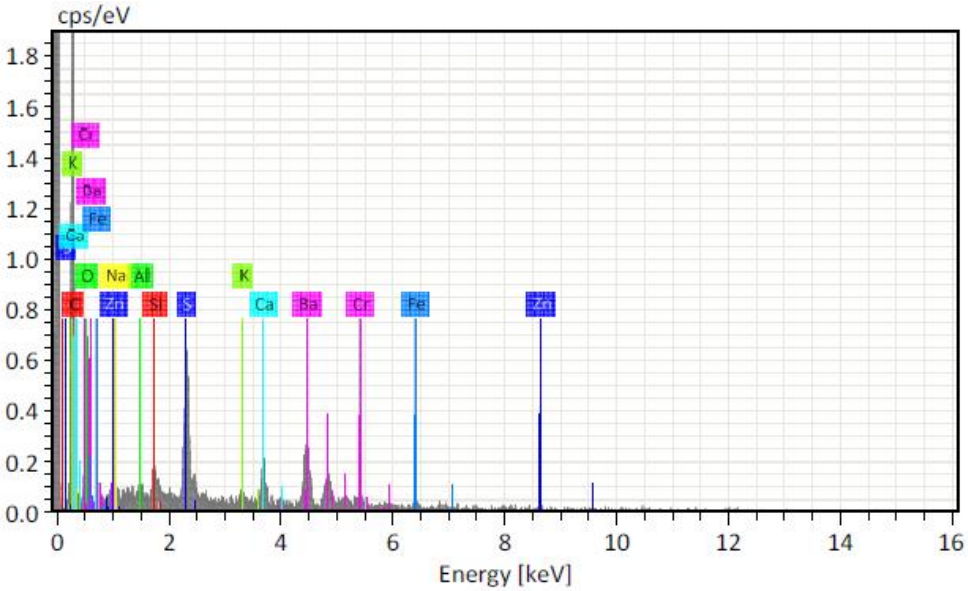


13 4

| Element | At. No. | Netto | Mass [%] | Mass Norm. [%] | Atom [%] | abs. error [%] (1 sigma) |
|---|---|---|---|---|---|---|
| Carbon | 6 | 3440 | 50.89 | 49.80 | 71.23 | 8.58 |
| Oxygen | 8 | 1171 | 19.96 | 19.53 | 20.97 | 4.35 |
| Sodium | 11 | 50 | 0.29 | 0.28 | 0.21 | 0.08 |
| Aluminium | 13 | 114 | 0.34 | 0.33 | 0.21 | 0.07 |
| Silicon | 14 | 229 | 0.60 | 0.58 | 0.36 | 0.08 |
| Sulfur | 16 | 1456 | 4.74 | 4.64 | 2.48 | 0.25 |
| Potassium | 19 | 141 | 0.64 | 0.63 | 0.28 | 0.09 |
| Calcium | 20 | 456 | 1.97 | 1.93 | 0.83 | 0.14 |
| Chromium | 24 | 178 | 1.64 | 1.61 | 0.53 | 0.17 |
| Iron | 26 | 44 | 0.52 | 0.51 | 0.16 | 0.11 |
| Zinc | 30 | 50 | 1.72 | 1.68 | 0.44 | 0.29 |
| Barium | 56 | 1665 | 18.89 | 18.48 | 2.31 | 0.79 |
| | | **Sum** | **102.19** | **100.00** | **100.00** | |

**Figure S.53** – FEG-SEM elemental analysis results obtained for sample 13

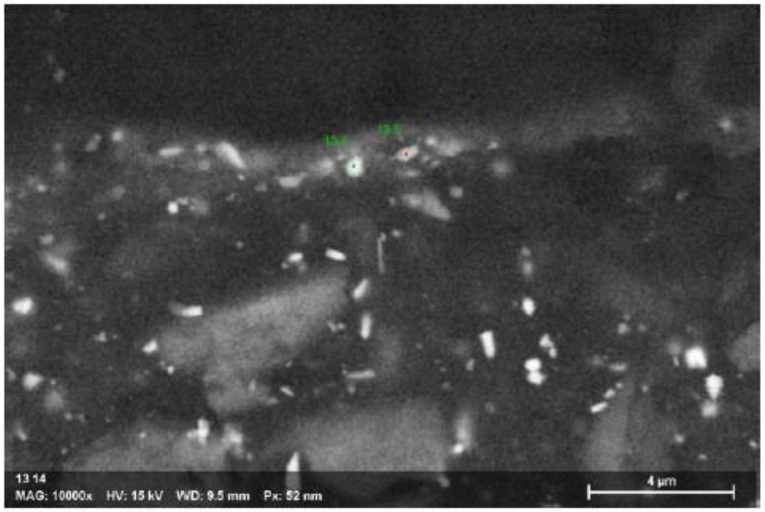


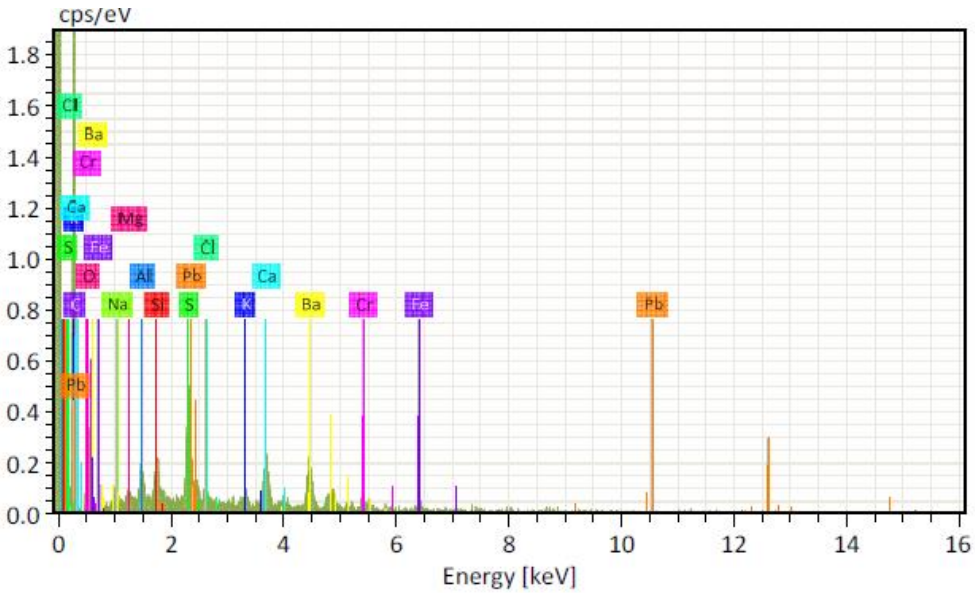


13 5

| Element | At. No. | Netto | Mass [%] | Mass Norm. [%] | Atom [%] | abs. error [%] (1 sigma) |
|---|---|---|---|---|---|---|
| Carbon | 6 | 2817 | 54.00 | 51.89 | 75.48 | 9.49 |
| Oxygen | 8 | 587 | 13.98 | 13.43 | 14.67 | 3.73 |
| Sodium | 11 | 124 | 1.24 | 1.19 | 0.90 | 0.19 |
| Magnesium | 12 | 64 | 0.42 | 0.41 | 0.29 | 0.09 |
| Aluminium | 13 | 310 | 1.52 | 1.46 | 0.95 | 0.16 |
| Silicon | 14 | 442 | 1.75 | 1.68 | 1.05 | 0.15 |
| Sulfur | 16 | 964 | 3.59 | 3.45 | 1.88 | 0.22 |
| Chlorine | 17 | 144 | 0.65 | 0.62 | 0.31 | 0.09 |
| Potassium | 19 | 100 | 0.41 | 0.40 | 0.18 | 0.07 |
| Calcium | 20 | 561 | 2.12 | 2.03 | 0.89 | 0.14 |
| Chromium | 24 | 143 | 1.96 | 1.88 | 0.63 | 0.21 |
| Iron | 26 | 58 | 1.08 | 1.04 | 0.32 | 0.18 |
| Barium | 56 | 1137 | 17.68 | 16.99 | 2.16 | 0.81 |
| Lead | 82 | 478 | 3.67 | 3.53 | 0.30 | 0.26 |

**Figure S.54** – FEG-SEM elemental analysis results obtained for sample 13

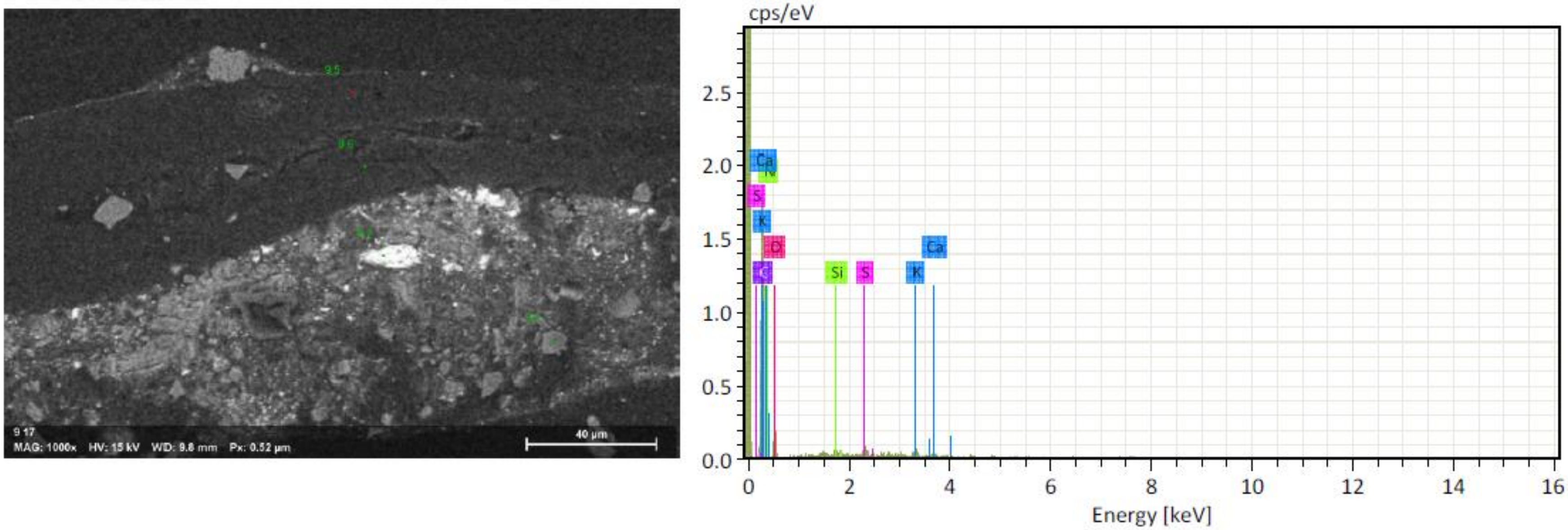


9 5

| Element | At. No. | Netto | Mass [%] | Mass Norm. [%] | Atom [%] | abs. error [%] (1 sigma) |
|---|---|---|---|---|---|---|
| Carbon | 6 | 2475 | 73.70 | 73.70 | 80.57 | 13.32 |
| Nitrogen | 7 | 0 | 0.00 | 0.00 | 0.00 | 0.00 |
| Oxygen | 8 | 307 | 21.47 | 21.47 | 17.62 | 7.07 |
| Silicon | 14 | 83 | 0.83 | 0.83 | 0.39 | 0.14 |
| Sulfur | 16 | 146 | 1.22 | 1.22 | 0.50 | 0.15 |
| Potassium | 19 | 131 | 1.76 | 1.76 | 0.59 | 0.20 |
| Calcium | 20 | 59 | 1.01 | 1.01 | 0.33 | 0.17 |
| | | **Sum** | **100.00** | **100.00** | **100.00** | |

**Figure S.55** – FEG-SEM elemental analysis results obtained for sample 9

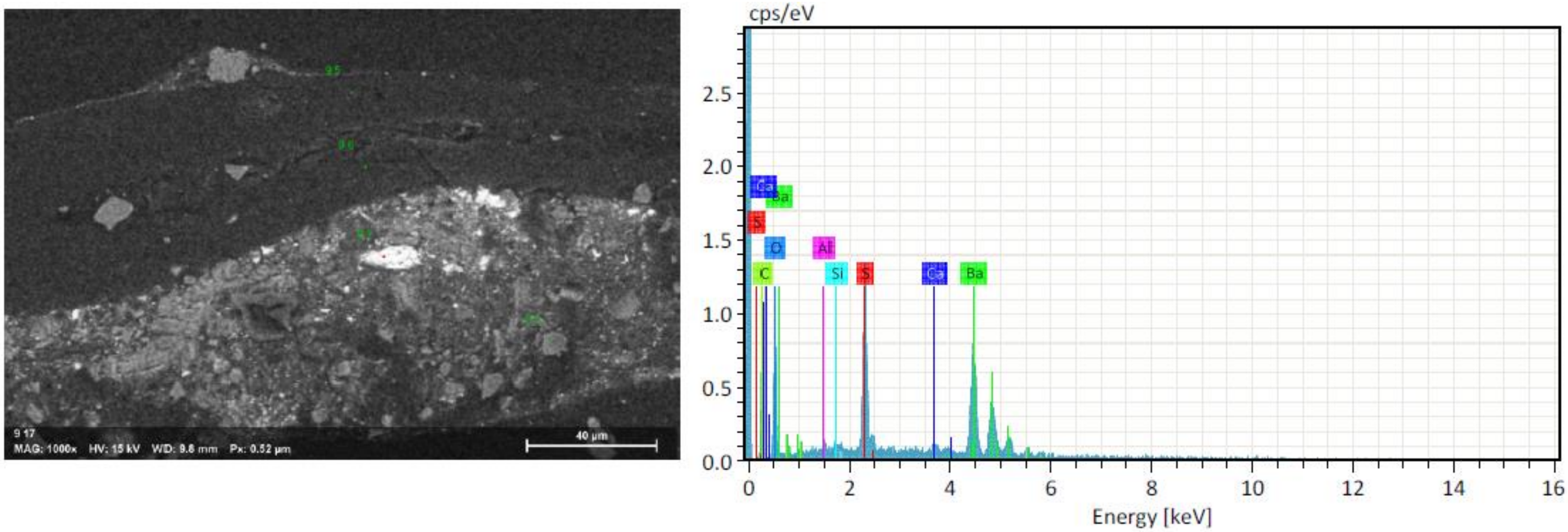


9 7

| Element | At. No. | Netto | Mass [%] | Mass Norm. [%] | Atom [%] | abs. error [%] (1 sigma) |
|---|---|---|---|---|---|---|
| Carbon | 6 | 1437 | 24.44 | 26.23 | 55.79 | 5.05 |
| Oxygen | 8 | 1289 | 14.48 | 15.53 | 24.81 | 3.09 |
| Aluminium | 13 | 74 | 0.35 | 0.38 | 0.36 | 0.08 |
| Silicon | 14 | 84 | 0.33 | 0.35 | 0.32 | 0.07 |
| Sulfur | 16 | 3076 | 11.81 | 12.67 | 10.09 | 0.52 |
| Calcium | 20 | 170 | 0.60 | 0.65 | 0.41 | 0.08 |
| Barium | 56 | 4797 | 41.19 | 44.20 | 8.22 | 1.40 |
| | | **Sum** | **93.20** | **100.00** | **100.00** | |

Figure S.56 – FEG-SEM elemental analysis results obtained for sample 9

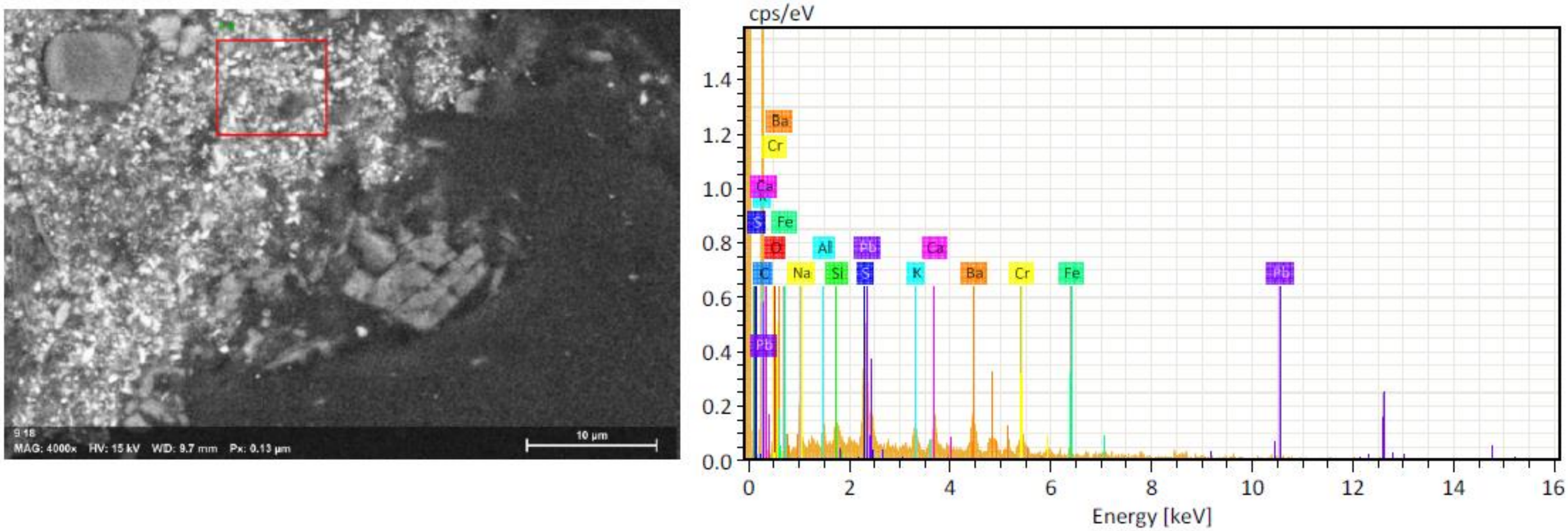


9 9

| Element | At. No. | Netto | Mass [%] | Mass Norm. [%] | Atom [%] | abs. error [%] (1 sigma) |
|---|---|---|---|---|---|---|
| Carbon | 6 | 2346 | 35.76 | 46.74 | 71.11 | 6.55 |
| Oxygen | 8 | 660 | 12.19 | 15.94 | 18.20 | 3.14 |
| Sodium | 11 | 247 | 1.57 | 2.06 | 1.64 | 0.20 |
| Aluminium | 13 | 159 | 0.61 | 0.80 | 0.54 | 0.09 |
| Silicon | 14 | 191 | 0.70 | 0.91 | 0.59 | 0.09 |
| Sulfur | 16 | 862 | 2.88 | 3.76 | 2.14 | 0.18 |
| Potassium | 19 | 205 | 0.89 | 1.16 | 0.54 | 0.10 |
| Calcium | 20 | 336 | 1.51 | 1.97 | 0.90 | 0.13 |
| Chromium | 24 | 271 | 2.90 | 3.79 | 1.33 | 0.24 |
| Iron | 26 | 80 | 1.14 | 1.49 | 0.49 | 0.17 |
| Barium | 56 | 846 | 10.74 | 14.04 | 1.87 | 0.55 |
| Lead | 82 | 813 | 5.62 | 7.34 | 0.65 | 0.34 |
| | | **Sum** | **76.52** | **100.00** | **100.00** | |

**Figure S.57** – FEG-SEM elemental analysis results obtained for sample 9

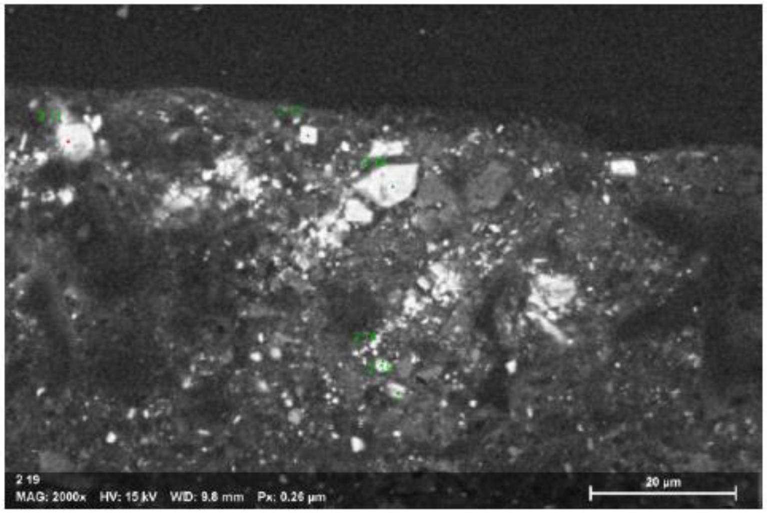


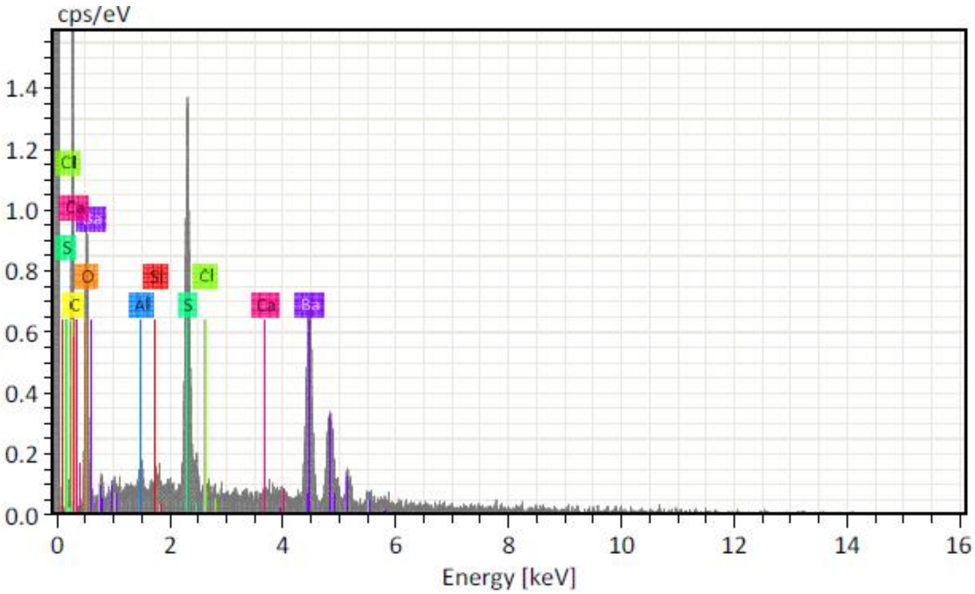


2 11

| Element | At. No. | Netto | Mass [%] | Mass Norm. [%] | Atom [%] | abs. error [%] (1 sigma) |
|---|---|---|---|---|---|---|
| Carbon | 6 | 2473 | 33.10 | 35.69 | 63.78 | 6.00 |
| Oxygen | 8 | 1548 | 16.19 | 17.46 | 23.42 | 3.29 |
| Aluminium | 13 | 119 | 0.33 | 0.35 | 0.28 | 0.06 |
| Silicon | 14 | 7 | 0.02 | 0.02 | 0.01 | 0.01 |
| Sulfur | 16 | 3318 | 8.90 | 9.60 | 6.43 | 0.40 |
| Chlorine | 17 | 0 | 0.00 | 0.00 | 0.00 | 0.00 |
| Calcium | 20 | 180 | 0.76 | 0.82 | 0.44 | 0.09 |
| Barium | 56 | 4385 | 33.43 | 36.05 | 5.63 | 1.15 |
| | | **Sum** | **92.72** | **100.00** | **100.00** | |

Figure S.58 – FEG-SEM elemental analysis results obtained for sample 2

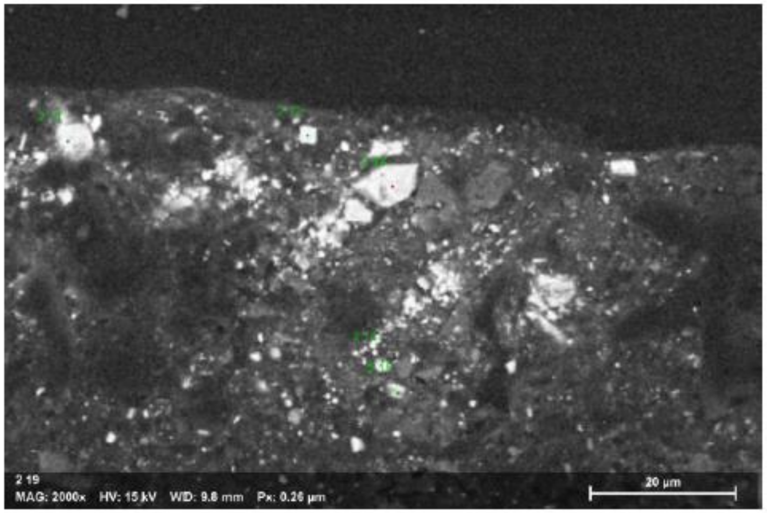


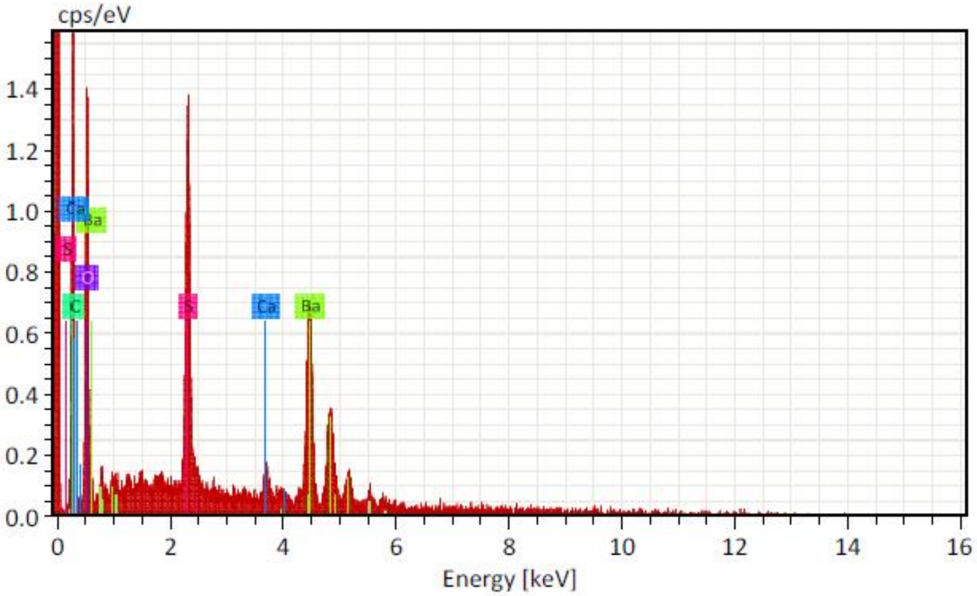


2 13

| Element | At. No. | Netto | Mass [%] | Mass Norm. [%] | Atom [%] | abs. error [%] (1 sigma) |
|---|---|---|---|---|---|---|
| Carbon | 6 | 2314 | 33.14 | 31.56 | 57.80 | 6.09 |
| Oxygen | 8 | 2168 | 23.14 | 22.03 | 30.29 | 4.33 |
| Sulfur | 16 | 3412 | 8.36 | 7.96 | 5.46 | 0.37 |
| Calcium | 20 | 248 | 0.75 | 0.72 | 0.39 | 0.08 |
| Barium | 56 | 4601 | 39.63 | 37.74 | 6.05 | 1.35 |
| | | **Sum** | **105.02** | **100.00** | **100.00** | |

Figure S.59 – FEG-SEM elemental analysis results obtained for sample 2

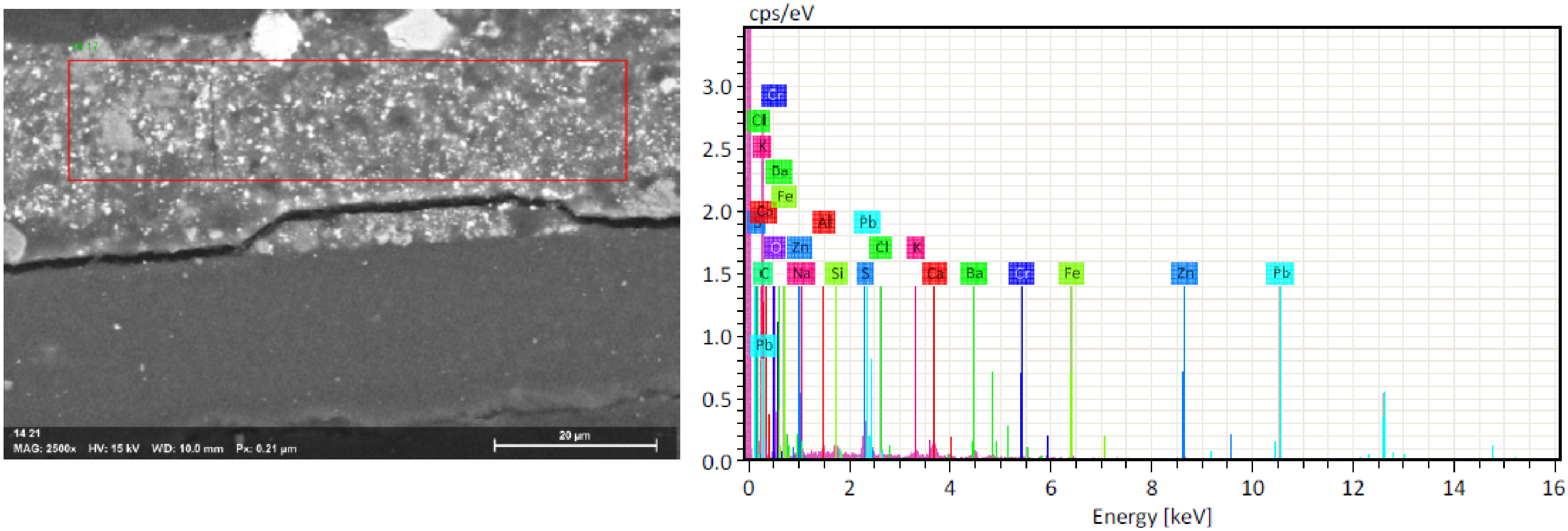


14 17

| Element | At. No. | Netto | Mass [%] | Mass Norm. [%] | Atom [%] | abs. error [%] (1 sigma) |
|---|---|---|---|---|---|---|
| Carbon | 6 | 3685 | 52.99 | 61.39 | 78.74 | 8.82 |
| Oxygen | 8 | 624 | 13.60 | 15.75 | 15.17 | 3.56 |
| Sodium | 11 | 185 | 0.95 | 1.10 | 0.74 | 0.14 |
| Aluminium | 13 | 119 | 0.42 | 0.49 | 0.28 | 0.08 |
| Silicon | 14 | 197 | 0.66 | 0.76 | 0.42 | 0.09 |
| Sulfur | 16 | 521 | 1.71 | 1.98 | 0.95 | 0.13 |
| Chlorine | 17 | 59 | 0.21 | 0.25 | 0.11 | 0.06 |
| Potassium | 19 | 135 | 0.66 | 0.77 | 0.30 | 0.09 |
| Calcium | 20 | 333 | 1.46 | 1.69 | 0.65 | 0.12 |
| Chromium | 24 | 69 | 0.74 | 0.86 | 0.26 | 0.12 |
| Iron | 26 | 56 | 0.89 | 1.04 | 0.29 | 0.15 |
| Zinc | 30 | 82 | 4.65 | 5.39 | 1.27 | 0.61 |
| Barium | 56 | 333 | 4.55 | 5.27 | 0.59 | 0.33 |
| [illegible] | [illegible] | [illegible] | [illegible] | [illegible] | [illegible] | [illegible] |

Figure S.60 – FEG-SEM elemental analysis results obtained for sample 14

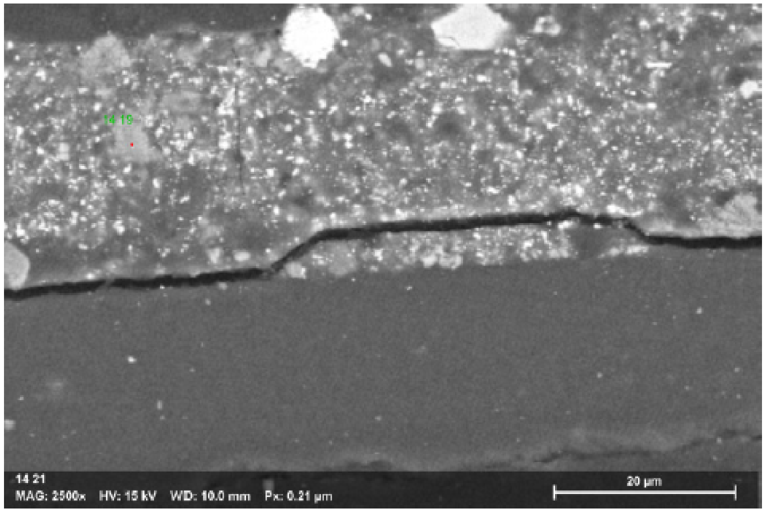


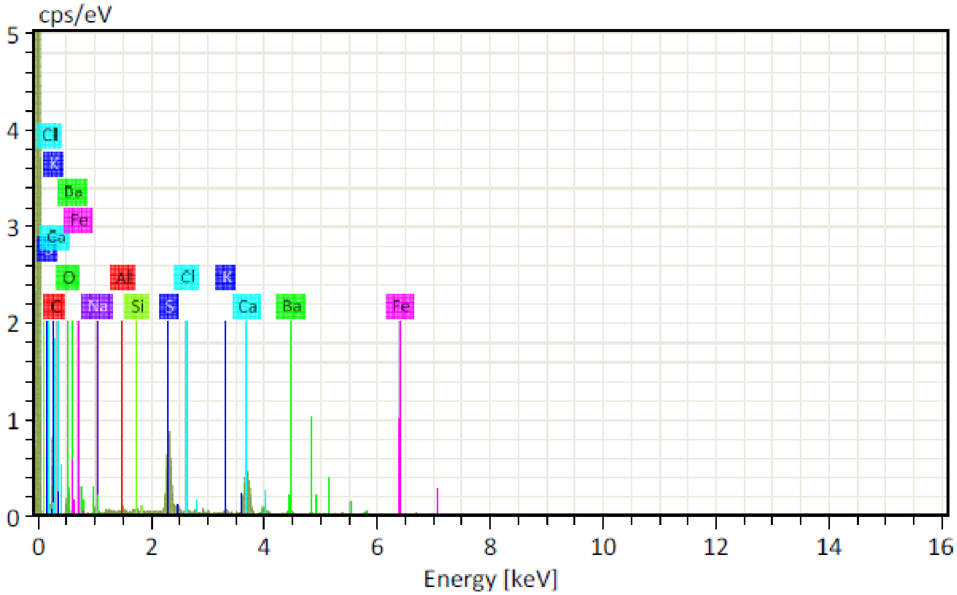


14 19

| Element | At. No. | Netto | Mass [%] | Mass Norm. [%] | Atom [%] | abs. error [%] (1 sigma) |
|---|---|---|---|---|---|---|
| Carbon | 6 | 2344 | 40.33 | 57.40 | 73.58 | 7.39 |
| Oxygen | 8 | 491 | 11.56 | 16.45 | 15.84 | 3.27 |
| Sodium | 11 | 124 | 0.88 | 1.25 | 0.84 | 0.14 |
| Aluminium | 13 | 54 | 0.20 | 0.28 | 0.16 | 0.06 |
| Silicon | 14 | 100 | 0.35 | 0.49 | 0.27 | 0.07 |
| Sulfur | 16 | 2096 | 5.66 | 8.05 | 3.87 | 0.28 |
| Chlorine | 17 | 14 | 0.04 | 0.06 | 0.03 | 0.01 |
| Potassium | 19 | 65 | 0.30 | 0.42 | 0.17 | 0.07 |
| Calcium | 20 | 1642 | 8.91 | 12.68 | 4.87 | 0.39 |
| Iron | 26 | 18 | 0.24 | 0.35 | 0.10 | 0.08 |
| Barium | 56 | 185 | 1.80 | 2.56 | 0.29 | 0.18 |
| | | **Sum** | **70.26** | **100.00** | **100.00** | |

**Figure S.61** – FEG-SEM elemental analysis results obtained for sample 14

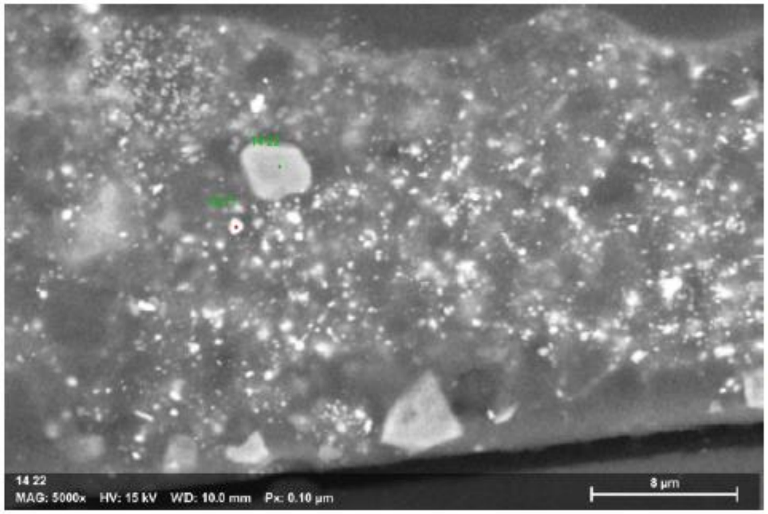


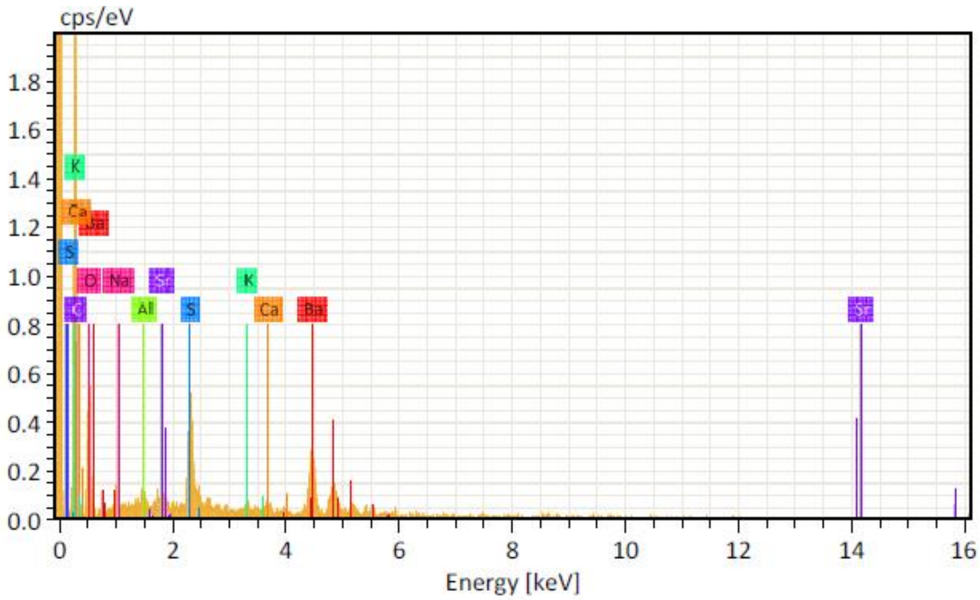


14 21

| Element | At. No. | Netto | Mass [%] | Mass Norm. [%] | Atom [%] | abs. error [%] (1 sigma) |
|---|---|---|---|---|---|---|
| Carbon | 6 | 3392 | 54.95 | 54.34 | 75.55 | 9.29 |
| Oxygen | 8 | 864 | 17.66 | 17.46 | 18.22 | 4.19 |
| Sodium | 11 | 167 | 1.06 | 1.05 | 0.76 | 0.15 |
| Aluminium | 13 | 116 | 0.49 | 0.48 | 0.30 | 0.08 |
| Sulfur | 16 | 1209 | 4.17 | 4.13 | 2.15 | 0.23 |
| Potassium | 19 | 65 | 0.28 | 0.27 | 0.12 | 0.06 |
| Calcium | 20 | 114 | 0.55 | 0.54 | 0.23 | 0.08 |
| Strontium | 38 | 89 | 0.53 | 0.52 | 0.10 | 0.09 |
| Barium | 56 | 1691 | 21.44 | 21.20 | 2.58 | 0.89 |
| | | **Sum** | **101.12** | **100.00** | **100.00** | |

**Figure S.62** – FEG-SEM elemental analysis results obtained for sample 14
